# P^+^ addition and transfer involving a tetraphosphenium ion†

**DOI:** 10.1039/d4sc06823h

**Published:** 2024-11-25

**Authors:** Roman Franz, Máté Bartek, Clemens Bruhn, Zsolt Kelemen, Rudolf Pietschnig

**Affiliations:** a Institute for Chemistry, CINSaT, University of Kassel Heinrich-Plett-Straße 40 34132 Kassel Germany pietschnig@uni-kassel.de https://www.uni-kassel.de/go/hybrid; b Department of Inorganic and Analytical Chemistry, Budapest University of Technology and Economics Műegyetem Rkp 3 1111 Budapest Hungary kelemen.zsolt@vbk.bme.hu

## Abstract

Triphospha[3]ferrocenophane Fe(C_5_H_4_-PTip)_2_PCl (Tip = 2,4,6-tri(isopropyl)phenyl) has been prepared and its suitability to generate the corresponding bisphosphanylphosphenium ion has been explored. By formal addition of P^+^ to the latter, an unprecedented tetraphosphenium ion forms which likewise is capable of P^+^ transfer and qualifies as Lewis superacid based on its computed fluoride ion affinity. As a solid, this species is stable and conveniently storable, featuring a remarkably long P–P bond (2.335(5) Å). From this tetraphosphenium ion, known and unprecedented triphosphenium ions have been generated *via* P^+^ transfer in solution, including a triphosphenium ion with P–H functionalities. Moreover, the latter has been obtained by tautomeric rearrangement from the corresponding hydrophosphane precursor. The bonding situation and details of the P^+^ transfer have been investigated by DFT calculations and experimental methods like multinuclear NMR spectroscopy and SC-XRD.

## Abstract 2

Formal addition of “P^+^” to an electrophilic bisphosphanylphosphenium ion, results in the formation of a dicationic tetraphosphenium ion featuring a remarkably long P–P bond between trivalent P-atoms and super Lewis acidic character.

## Graphical abstract

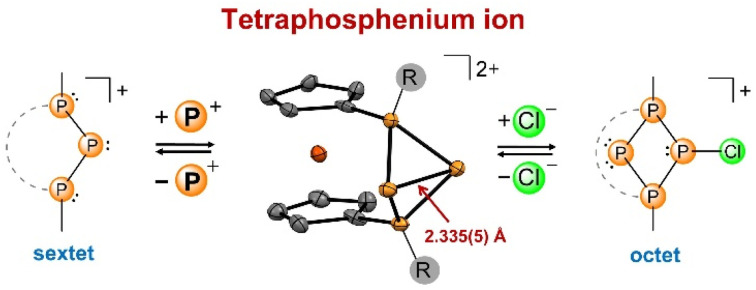

## Introduction

**Fig. 1:**
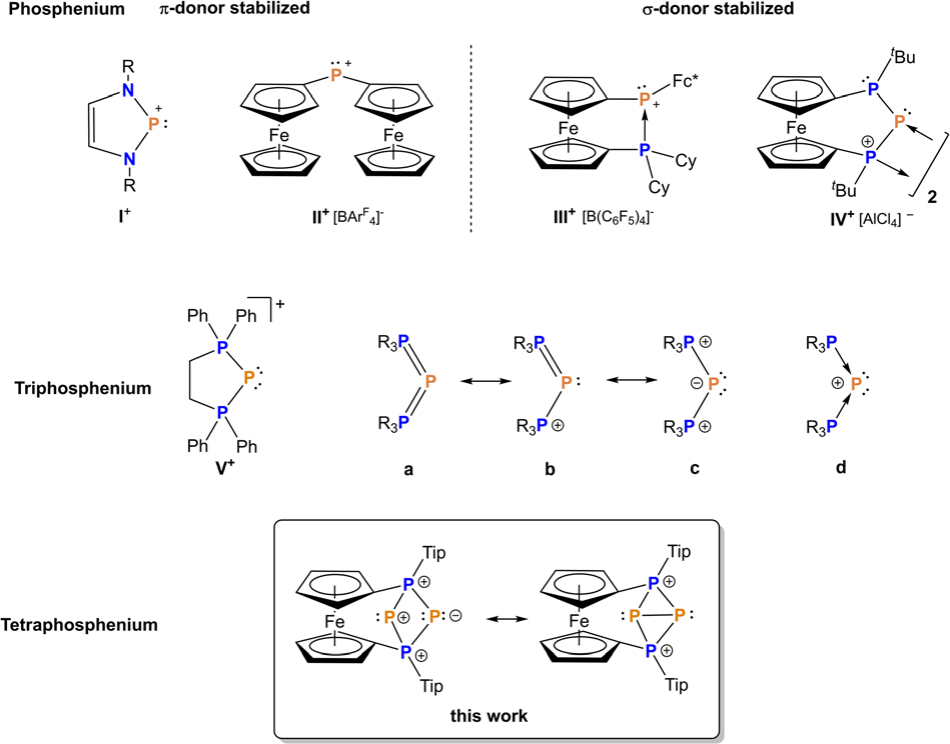
Comparison of phosphenium ions I^+^, II^+^,^50^III^+^,^42^ and IV^+^,^54^ (top) to triphosphenium ions V^+^ (middle) and a tetraphosphenium ion (bottom).

The transfer of single phosphorus atoms has emerged as a hot topic in recent years with a special focus on P(0) or P^−^ fragments.^1–14^ By contrast, P^+^ ion transfer is far less explored,^15–23^ and in addition needs to be distinguished from the transfer of substituted P(i) fragments with more than one atom, such as phosphinidenes. So called triphosphenium ions are established reagents for the transfer of formally unsubstituted P^+^ and have been carefully studied by Macdonald and co-workers in recent years.^15–24^ The first triphosphenium ions have been reported by Schmidpeter *et al.* in the 1980s as the first isolable P(i) compound in which the formal charge of P^+^ is also preserved as a net charge.^25,26^ It needs to be pointed out that – despite their similar name – the above mentioned triphosphenium ions are only distantly related to actual phosphenium ions [PR_2_]^+^ with the latter featuring an electron sextet, in contrast to triphosphenium ions with an electron octet at the central phosphorus atom (Fig. 1).^27^ Phosphenium ions are valence isoelectronic and isolobal to neutral carbene analogues such as [ER_2_] (E = C, Si, Ge, Sn, Pb).^28,29^ In addition, the positive charge imparts pronounced electrophilic character to phosphenium ions, comparable to Lewis acidic carbenium or silicenium ions [R_3_E]^+^ (E = C, Si) which in turn lack an electron lone pair, however.^30^ Consequently, phosphenium ions are highly reactive species which, for a long time, could only be detected in the gas phase until the development of thermodynamically stabilized examples,^31–42^ with the term NHP being in use for N-heterocyclic phosphenium ions of the type I^+^ (Fig. 1) in analogy to isolobal NHCs.^40,43–46^

Thermodynamic stabilization has been achieved for carbon substituted (nitrogen-free) phosphenium ions as well, using ylidic^47,48^ or electron-rich olefinic carbon atoms,^49^ or ferrocenyl substituted phosphenium ions such as II^+^ and III^+^ (Fig. 1).^42,50–52^ Moreover, Beckmann *et al.* succeeded in isolating a kinetically stabilized phosphenium ion recently.^53^ For increased reactivity, *in situ*-formed, non-stabilized phosphenium ions have been employed as versatile reaction partners to construct polyphosphorus compounds, activate small molecules and alkenes.^34,54–61^ In line with this, bisphosphanylphosphenium ions IV^+^, which could be referred to as P-heterocyclic phosphenium ions (PHPs) in analogy to the above mentioned NHPs, feature insufficient stabilization by adjacent phosphanyl units and consequently undergo either dimerization or fragmentation^54,62^ involving P–C bond activation, with the P_3_-unit remaining intact (Fig. 1).^54,62^

Using an *in situ* generated bisphosphanylphosphenium ion equipped with a suitable substitution pattern, we report here the first transfer of formally monoatomic P^+^ to a phosphenium cation, resulting in the formation of a characteristic tetraphosphorus dication which we refer to as tetraphosphenium ion. This contrasts other known examples of P^+^ transfer proceeding to nucleophilic substrates such amines, phosphanes, NHCs or carbonylmetallates to name just a few.^15–17,63^

## Results and discussion

**Scheme 1:**
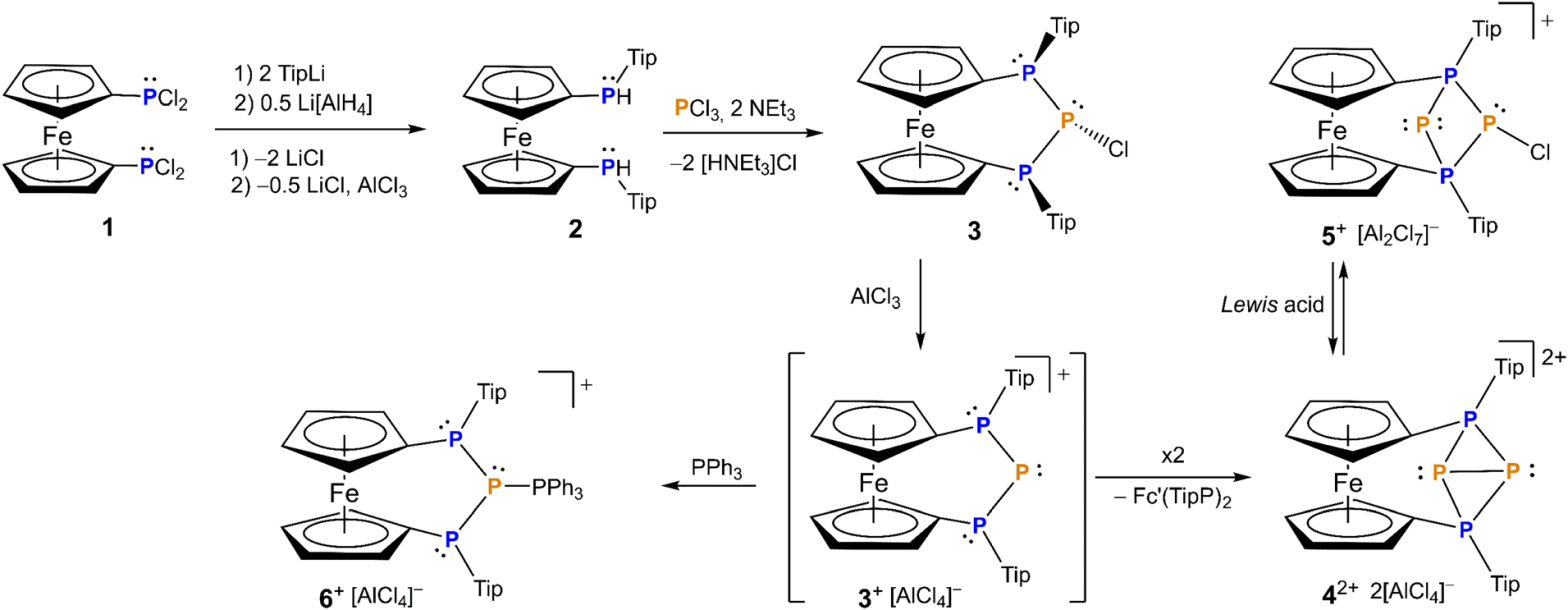
Synthetic pathway to phosphenium ion 3[AlCl_4_], stabilized phosphenium ion 6[AlCl_4_] and tetraphosphenium ion 4[AlCl_4_]_2_ with rearrangement to triphosphenium ion 5[Al_2_Cl_7_].

As a starting point, we introduced 2,4,6-tri(isopropyl)phenyl (Tip) as a so far unexplored substituent at the phosphorus atoms of the [3]ferrocenophane scaffold following a previous established substitution and condensation sequence (Scheme 1).^64^ We anticipated that the increased steric congestion would supply sufficient steric protection to prevent dimerization observed for the ^*t*^Bu substituted heterocarbenes.^54,65^

To this end 1,1-bis(dichlorophosphanyl)ferrocene^66^ is reacted with two equivalents of 2,4,6-tri(isopropyl)phenyl lithium (Tip–Li) then the resulting chlorophosphane is transformed into the secondary hydrophosphane 2 which subsequently condenses with PCl_3_ in the presence of base to form [3]ferrocenophane 3 (Scheme 1). Given the stereogenic nature of the phosphorus atoms in the terminal positions, it should be mentioned that prior to the ring closure the ferrocenophane precursor is a mixture of the corresponding *rac* and *meso* diastereomers of 2 for which *rac*2 epimerizes upon reaction to 3. The ferrocene bridged triphosphanyl scaffold limits the number of diastereomers originating from the presence of the *P*-stereogenic centers.^64^ Interestingly, for Tip-substituted 3 exclusive formation of the *meso-trans*-isomer is observed, where *trans* refers to the orientation of the chlorine with respect to the Tip substituents. The computed Gibbs free energy difference between the *cis* and its corresponding *trans* isomer is small (Δ*G* = 2.9 kcal mol^−1^ see Computational details in ESI†), comparable to the value obtained in case of the ^*t*^Bu-substituted counterpart, where both isomers were observed.^64^ Assuming that interconversion of the isomers is unlikely due to the high inversion barrier of the phosphorus centers, the selective formation of the *trans* isomer of 3 can be attributed to kinetic factors rather than to its thermodynamic stability.

The ^31^P NMR spectrum of [3]ferrocenophane 3 features an AX_2_ spin system, with the signal of the central phosphorus atom (*δ*(^31^P) = 91.2 ppm) split into a triplet, whereas the two outer phosphorus nuclei resonating at −28.1 ppm feature a doublet splitting. The low ^1^*J*_PP_ coupling constant of 177 Hz in 3 is in agreement with the formation of the *meso-trans* diastereomer since significantly higher values (*ca.* 350 Hz) are generally observed for *meso-cis*-triphospha[3]ferrocenophanes.^64^ Although we were able to grow small orange crystals of 3, these were not suitable for X-ray crystallography owing to extremely poor scattering. The identity and purity of 3 were further confirmed by ^1^H, ^13^C-NMR spectroscopy and elemental analysis. The ^31^P resonances of both diastereomers of 2 are located at −97.9 ppm (as two overlapping resonances) featuring ^1^*J*_PH_-coupling of 226 Hz. Identity and purity of 2 were further confirmed by ^1^H, ^13^C-NMR spectroscopy, mass spectrometry, elemental analysis and X-ray crystallography (Fig. S1†). The latter findings support significant steric congestion, with the angular sum at the phosphorus atoms (∑P1 = 332(4)° and ∑P2 = 325(3)°) in 2 being remarkably high and comparable to extremely bulky triarylphosphanes such as P(Mes)_3_ (330°)^67^ or P(Tip)_3_ (337°).^67^

**Fig. 2:**
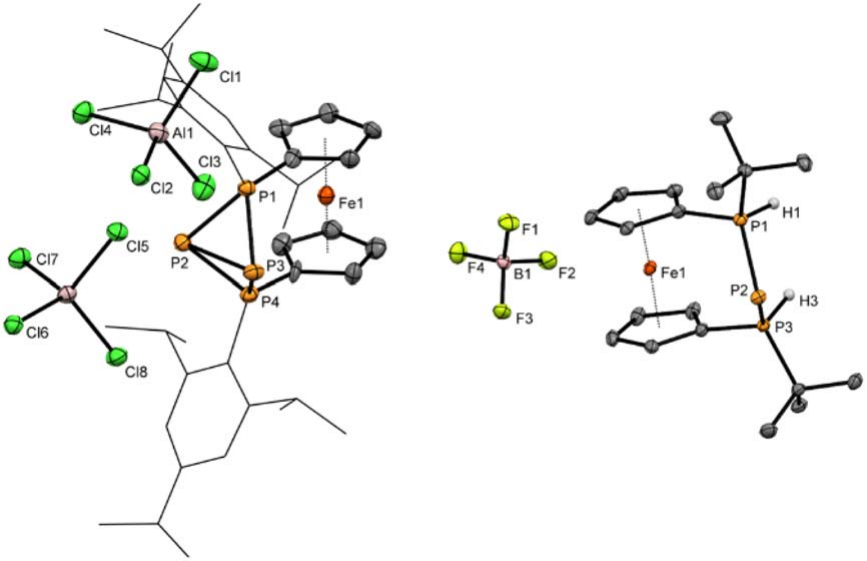
Molecular structures of 4[AlCl_4_]_2_ and 8c[BF_4_] in the solid state. Ellipsoids are shown at 30% probability. All hydrogen atoms except H1 and H3, which are bonded to phosphorus atoms are omitted for clarity. The Tip substituents at P1 and P4 in 4[AlCl_4_]_2_ are shown as wireframe. Selected bond lengths and angles in 4[AlCl_4_]_2_: P1–P2 2.184(5) Å, P1–P3 2.171(5) Å, P2–P3 2.335(5) Å, P2–P4 2.194(5) Å, P3–P4 2.173(5) Å; 8c[BF_4_]: P1–P2 2.1284(7) Å, P2–P3 2.1265(7) Å, P1–H1 1.28(2) Å, P2–H2 1.26(2) Å, PPP-angle 89.50(2)°.

With chlorophosphane 3 in hand, we set out to investigate chloride abstraction to the respective phosphonium ion. While with GaCl_3_ or Li[Al(OC(CF_3_)_3_)_4_] no selective reactivity was found, for AlCl_3_ complete and selective P^+^ transfer to dicationic 4^2+^ and diphosphane Fc'(TipP)_2_ was observed (Scheme 1). As intended the bulky Tip substituents successfully prevent dimerization of 3^+^, which had been observed for its ^*t*^Bu substituted analog IV^+^ (Fig. 1).^54^ However, stabilization of the flanking phosphanyl units is insufficient to isolate the free phosphenium ion 3^+^,^68^ resulting in formal elimination of “P^+^” which is transferred to a second unit of transient phosphenium cation 3^+^ forming the thermodynamically more stable dication 4^2+^. This hypothesis is thermodynamically plausible, as confirmed by the exergonic computed reaction Gibbs free energy for the “P^+^” transfer to cation 3^+^ (Δ*G* = −5.1 kcal mol^−1^, Computational details in ESI†). At this point, it is important to highlight that the formation of the corresponding bisphosphanylphosphenium dimer (analogue of (IV^+^)_2_) is thermodynamically favored (Δ*G* = −12.5 kcal mol^−1^) over the formation of 4^2+^ (Δ*G* = −5.1 kcal mol^−1^). The exergonic dimerization process, even in the presence of bulky groups, indicates the outstanding stability of this dimeric structural motif^54,69,70^ and implies that the formation of 4^2+^ is under kinetic control (proposed mechanism in Scheme S1 in the ESI†). Again, ^31^P NMR spectra are helpful to establish the connectivity in 4^2+^ featuring two triplet resonances centered at *δ* = −213.9 ppm (central phosphanyl units) and *δ* = 24.0 ppm (tetracoordinated phosphorus atoms) with a ^1^*J*_PP_ coupling constant of 304 Hz, which is consistent with a rigid tetraphosphabicyclobutane. The identity and purity of 4[AlCl_4_]_2_ was confirmed by ^1^H, ^13^C, ^27^Al NMR spectroscopy and elemental analysis. The constitution of 4[AlCl_4_]_2_ has been further corroborated by single crystal X-ray diffraction (Fig. 2).

The bisphosphonium ion 4^2+^ crystallizes without direct contact to its [AlCl_4_]^−^ counter anions and with almost plane-parallel arrangement of the Cp ligands (*α* = 2.7(2)°) indicating an unstrained ferrocenophane structure. The molecular structure in the solid state of 4[AlCl_4_]_2_ shows a P_4_ fold angle of 72.1(2)°, which is usually found for strongly puckered P_4_ rings in diaryltetraphosphabicyclobutanes.^71–73^ In line with this a strongly distorted trigonal pyramidal geometry is found at P2 and P3 with an angular sum of ΣP2 = 200.2(5)° and ΣP3 = 202.5(5)°. The P–P bond lengths [2.184(5) Å (P1–P2) and 2.171(5) Å (P1–P3)] between the tetrasubstituted outer and trisubstituted central phosphorus atoms are in the range of common P–P single bonds. Interestingly the bond between the two central phosphorus atoms is significantly elongated [2.335(5) Å P2–P3]. To the best of our knowledge, the tetraphosphenium salt 4[AlCl_4_]_2_ contains the longest documented P–P single bond between two tricoordinated phosphorus atoms in a functionalized “P_4_”. Closely related to 4^2+^ are P_4_-adducts with cations such as Ag^+^, NO^+^, or PPh_2_^+^ reported by Krossing, Weigand and Riedel or the edge protonated P_4_H^+^ reported by Müller and Riedel.^57,74–77^

The bonding situation of 4^2+^ was explored by DFT calculations. Topological analysis was performed by the means of Bader's atoms in molecules (AIM) theory, and a bond critical point with electron density of 0.092 au was found between P2 and P3. In agreement with the shorter bond length, the electron density is higher (0.122 a.u.) in the bond critical points between atoms P1 and P2 (or P1 and P3). By examining the dimensionless ratios defined by Bader^78^ and Espinosa^79^ and other computed parameters, the interaction can be classified as a covalent bond (Tables S2–S5†). The Laplacian of the electron density along the P2–P3 bond path (∇^2^(*r*) function) shows a high degree of symmetry, with a local maximum of −0.0314 au at the bond critical point (see Fig. S2†). The rather weak character of the P2–P3 bond is reflected by the computed quantum theory of atoms in molecules (QTAIM) data, Mayer bond, and delocalization indices (see Tables S2–S4 in the ESI†) and the gradient plot of the Laplacian plot (see Fig. S3 and S4 in the ESI†). Natural resonance theory analysis proposes only one leading resonance structure for tetraphosphenium ion 4^2+^ with 70% weight, which is represented by the Lewis structure depicted in Scheme 1 and in Fig. 4 (structure D) (more details in the ESI†).

**Fig. 3:**
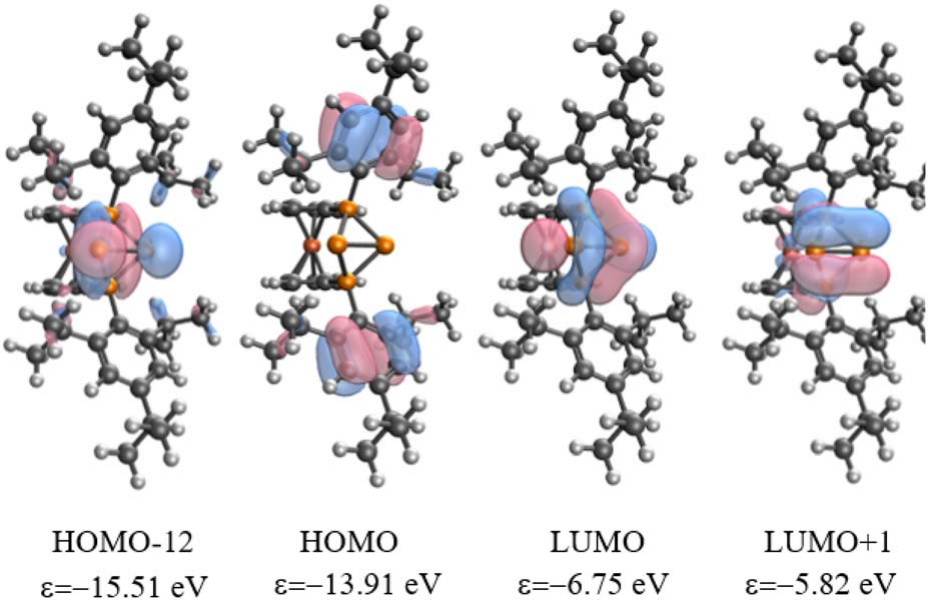
Selected Kohn–Sham molecular orbitals of 4^2+^ at ωB97X-D/def2-TZVP.

**Fig. 4:**
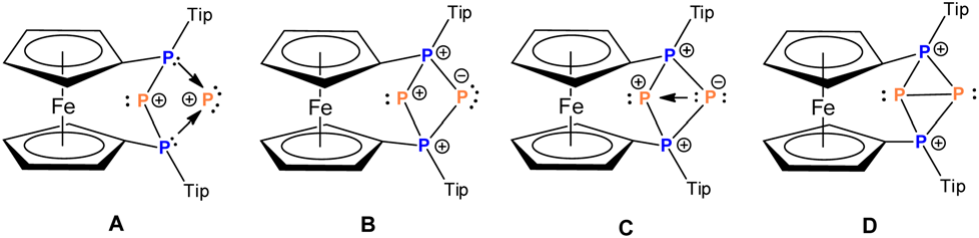
Alternative Lewis-type descriptions of 4^2+^.

**Scheme 2:**
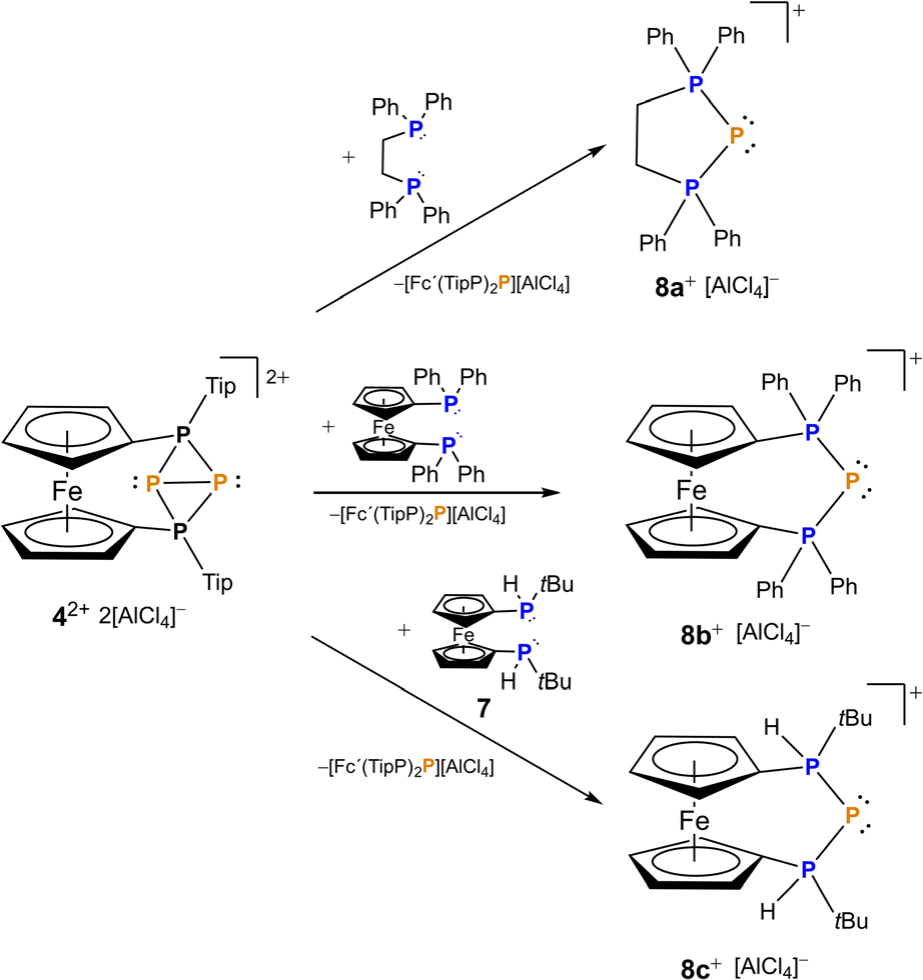
Synthesis of triphosphenium ions 8a–c*via* formal P^+^ transfer using 4[AlCl_4_]_2_ starting from the corresponding bisphosphane.

**Scheme 3:**
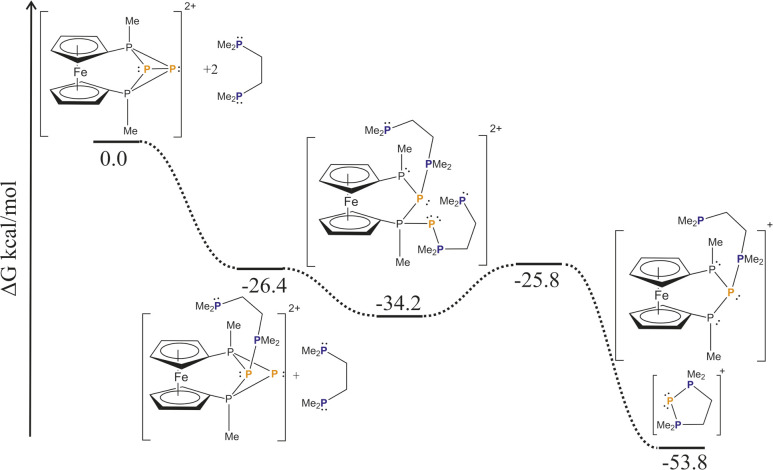
Calculated reaction mechanism of the P^+^ transfer reaction.

The analysis of Kohn–Sham orbitals shows that all four phosphorus atoms make significant contributions to the unoccupied orbitals (Fig. 3). The LUMO is basically the combination of the empty p_*z*_ orbitals at the central phosphorus atoms and the antibonding σ* of the P2–P3 bond, which foreshadows the reactivity of the central phosphorus atoms towards nucleophiles. The orbital-weighed Fukui function (which is a useful tool to identify the reactive site of a system)^80^ verified the most electrophilic character of the central phosphorus atoms as well (Fig. S2 and S3 in the ESI†). The computed natural charge at each of the central phosphorus atoms is 0.21 which is in close agreement with the charges computed within QTAIM atomic basins. In view of these data, a possible alternative description of 4^2+^ could be a phosphenium ion intramolecularly stabilized by a triphosphenium ion (*cf.*C in Fig. 4), where the triphosphenium part emerges from twofold coordination of P^+^ by the phosphanyl groups of former 3^+^ (*cf.*A and B in Fig. 4). However, the natural bond order in D, and QTAIM results indicate a purely covalent central P–P bond in line with the above-mentioned NRT results for 4^2+^. Nonetheless, polar structures B or C, whilst not representative of the bonding situation, are useful to understand the reactivity of 4^2+^ (Schemes 1–3).

In order to evaluate the Lewis acid strength of 4^2+^, its fluoride ion affinity (FIA) was computed (for single point energy calculations PCM = THF solvent model was applied), which is 87.2 kcal mol^−1^. This value is significantly higher than the average of 27 dicationic compounds (40.8 kcal mol^−1^) recently investigated by Greb *et al.*^81^ and suggests super Lewis acidic character. (The corresponding vacuum phase FIA is 239.0 kcal mol^−1^ for 4^2+^ and the average reported by Greb is 215 kcal mol^−1^). In order to get a more precise picture (applying the same computational protocol) we have calculated phosphorus containing dicationic systems, where the positive charge is not localized at one center similar to 4^2+^. Among the investigated cases (Table S6 in the ESI†) Dielmann's tricoordinate phosphorus dication [P(Nim)_3_]^2+^ (im = 1,3-diisopropyl-4,5-dimethylimidazolin-2-yliden) exhibits a very similar FIA value (87.9 kcal mol^−1^), which was considered as Lewis superacid as well.^82^

Tetraphosphenium salt 4[AlCl_4_]_2_ is poorly soluble in halogenated solvents like chloroform, dichloromethane or 1,2-difluorobenzene. Isolated 4[AlCl_4_]_2_ can be stored as solid under inert atmosphere for several weeks without decomposition but has a limited lifetime in solution. In solution, 4[AlCl_4_]_2_ transforms quantitatively within several hours to a new product, which could be assigned to its chloride adduct 5[Al_2_Cl_7_] representing the first bicyclic triphosphenium ion (Scheme 1). The calculated reaction Gibbs free energy of the process is only −0.1 kcal mol^−1^. Since the small negative Δ*G* value does not exclude the reversibility of the process, the quantitative back-reaction to tetraphosphenium ion 4^2+^ was tested and after the addition of further Lewis acid (AlCl_3_ or Li[Al(OC(CF_3_)_3_)_4_]) 4^2+^ was detected. The mono cation 5[Al_2_Cl_7_] shows characteristic signals in the ^31^P NMR spectrum (Table 1).

**Table 1:** Summary of ^31^P NMR data of compounds 3–6 and 8c (without consideration of ^1^H–^31^P coupling)

Compound	*δ*(^31^P) [ppm]	|*J*_PP_| [Hz]	Spin system
3	91.2(t), −28.1(d)	177 (^1^*J*_AX_)	AX_2_
4	24.0(t), −213.9(t)	304 (^1^*J*_AX_)	A_2_X_2_
5	19.5(dd), −39.8(dt), −130.7(td)	372 (^1^*J*_AX_), 337 (^1^*J*_MX_), 108 (^2^*J*_AM_)	AMX_2_
6	43.2(dd), 22.3(dt), 5.2(dt)	174 (^1^*J*_AX_), 72 (^2^*J*_MX_), 379 (^1^*J*_AM_)	AMX_2_

Thus, the resonance of the two chemically equivalent terminal phosphorus atoms at 19.5 ppm splits into a doublet of doublets owing to coupling with the central phosphanide center (^1^*J*_PP_ = 372 Hz) and the central P–Cl unit (^1^*J*_PP_ = 337 Hz). The same couplings are found for the signals of the central disubstituted phosphorus atom resonating at −130.7 ppm (^1^*J*_PP_ = 372 Hz, ^2^*J*_PP_ = 108 Hz) and the chlorine substituted phosphorus atom at −39.8 ppm (^1^*J*_PP_ = 337 Hz, ^2^*J*_PP_ = 108 Hz) which in turn split into doublets of triplets. The identity and purity of 5[Al_2_Cl_7_] was further confirmed by ^1^H, ^13^C NMR spectroscopy and elemental analysis. Since initially formed 3^+^ was not directly observed spectroscopically, the formation of 3^+^ as an intermediate was verified by trapping the phosphenium ion with the established Lewis base PPh_3_, leading to the formation of the expected triphenylphosphane-stabilized phosphenium ion 6[AlCl_4_] (Scheme 1). Interestingly, 6^+^ exhibits higher stability by 15.0 kcal mol^−1^ (Scheme S2 in ESI†), than its ^*t*^Bu substituted counterpart.^54^ The PPh_3_-adduct 6[AlCl_4_] shows three different resonances in the ^31^P NMR spectra with expected splitting patterns similar to a triphenylphosphane-stabilized phosphenium ion reported only recently.^54^ The two chemically equivalent outer phosphorus nuclei show a resonance at 43.2 ppm, split into a doublet of doublets due to the coupling to the central phosphorus atom (^1^*J*_PP_ = 174 Hz) and the PPh_3_ group (^2^*J*_PP_ = 72 Hz). The signal of the central phosphorus atom resonates at 5.2 ppm and splits into a doublet of triplets owing to coupling with the two chemically equivalent outer phosphanyl groups and the PPh_3_ fragment (^1^*J*_PP_ = 379 Hz). The signal of the PPh_3_ group in 6[AlCl_4_] is found at 22.3 ppm with the expected doublet of triplet pattern resulting from the P–P couplings described above. Identity and purity of 6[AlCl_4_] was further confirmed by ^1^H, ^13^C, ^27^Al NMR spectroscopy and elemental analysis.

The bonding situation along with the exceptionally long P–P bond in 4[AlCl_4_]_2_ sparked us to explore the reactivity of this unprecedented species. Since 4^2+^ originates from P^+^ transfer itself and its formation was not highly exergonic (Δ*G* = −5.1 kcal mol^−1^), we were wondering whether this P^+^ fragment may be further transferred to other substrates. Thus, we reacted 4[AlCl_4_]_2_ with different bisphosphanes such as dppe (1,2-bis(diphenylphosphino)ethane), dppf (1,1′-bis(diphenylphosphino)ferrocene) and prochiral 1,1′-bis(*tert*-butylphosphino)ferrocene 7. In all cases a formal P^+^ transfer was obtained leading to selective formation of the corresponding triphosphenium ions 8a–c (Scheme 2). As a by-product, the corresponding phosphenium cations stabilized by a second equivalent of bisphosphane were observed.

Our DFT calculations (Scheme 3, a model system with Me substituents at the phosphorus atoms was used, ωB97X-D/def2-SVP) reveal that the nucleophilic attack of the bisphosphane to one of the central phosphorus atoms (P2) is a barrierless process, resulting in the cleavage of the central P2–P3 bond. The addition of a second bisphosphane to the other central phosphorus atom (P3) leads to the breakage of the bond between the central and the outer phosphorus atoms (P1–P3) and similar to the first addition it proceeds without any significant barrier. In the next step, the P3–P4 bond cleaves, and at the same time, the second phosphane unit of the bisphosphane closes the ring with the originally P3 phosphorus atom. The overall process has a significant thermodynamic driving force (−53.8 kcal mol^−1^).

**Scheme 4:**
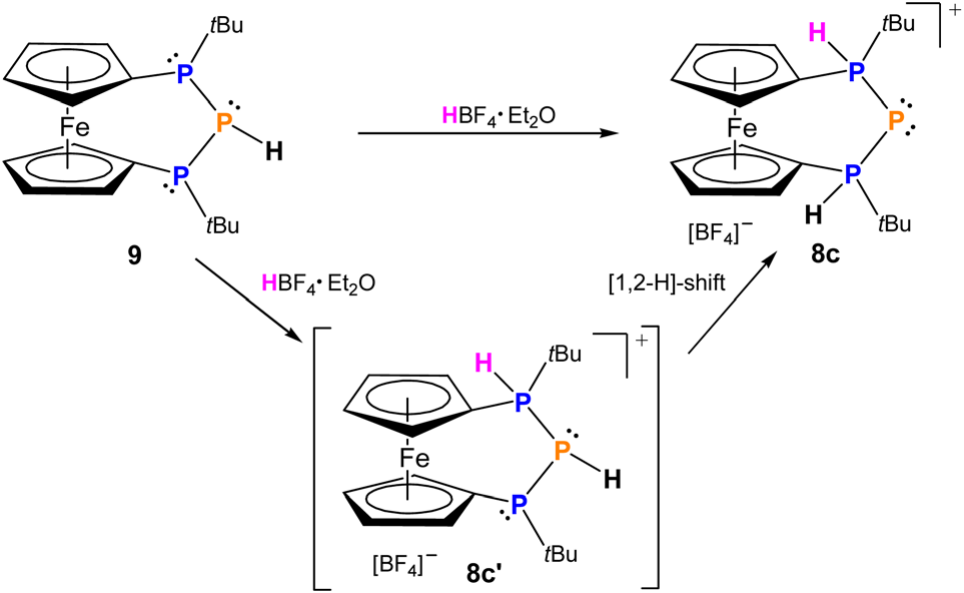
Synthesis of triphosphenium ion 8c*via* protonation of triphosphane 9 with HBF_4_.

While triphosphenium ions 8a,b are already known in the literature and their successful formation was verified by ^31^P-NMR spectroscopy,^83,84^8c represents the first triphosphenium ion carrying hydrogen substituents at both phosphonium centers, in addition to one *tert*-butyl group. Given the unique possibilities of attaching or abstracting protic hydrogen atoms we explored an alternative access to 8c starting from literature known 9. The calculated proton affinity of 9 is 270.7 kcal mol^−1^, and we found that HBF_4_ is able to protonate secondary triphosphane 9 inducing a [1,2-H] shift of the proton already present at the central phosphorus atom resulting in selective formation of 8c (Scheme 4), which is more stable by 1.9 kcal mol^−1^, than the originally formed intermediate (8c′). Despite its simplicity this straightforward approach to triphosphenium ions *via* protonation of a secondary triphosphane is unprecedented in the current literature. A clear advantage over previously published protocols emerges from the selectivity of the reaction, as no byproducts are formed and 8c can be isolated in good yields (65%) after first recrystallization. Regardless of whether 8c is formed *via* a formal “P^+^” transfer to bisphosphane 7 or protonation of triphospha[3]ferrocenophane 9 the formation proceeds stereoselectively and 8c is obtained in the *meso* form only, while there is no evidence for the appearance of its *rac* diastereomer neither in solution nor in solid state. In the ^31^P-NMR spectra, protonation of the outer phosphorus atoms entails a significant downfield shift of these nuclei by more than 50 ppm resulting in a chemical shift at 42.5 ppm in 8c. In turn, the central phosphorus atom encounters a drastic high field shift and resonates at −195.2 ppm in 8c and the AX_2_ spin system features a ^1^*J*_PP_-coupling constant of 456 Hz. These values are fully consistent with those of literature known triphosphenium ions reported by Schmidpeter or Macdonald.^16,26,83,85^ In addition the signal of the two outer phosphorus atoms in 8c splits into a doublet with a ^1^*J*_PH_ coupling constant of 468 Hz, which is typical for proton substituted phosphonium ions, as is the *δ*(^1^H) = 7.07 ppm. The identity and purity of isolated 8c was further corroborated by ^1^H-, ^11^B-, ^13^C, ^19^F-NMR, mass spectrometry and elemental analysis.

In the solid state the *meso* arrangement in 8c was confirmed by single crystal X-ray diffraction (Fig. 2). The ferrocenophane based triphosphenium ion 8c crystallizes in an almost ecliptic conformation of the ferrocene unit (*τ* = 4.58(8)°) and plane-parallel arrangement of the Cp rings (*α* = 1.24(8)°). For the central phosphorus atom P2 of the P_3_-bridge, symmetric contacts to the adjacent phosphonium centers P1 (2.1284(7) Å (P1–P2)) and P3 (2.1265(7) Å (P2–P3)) are found. The P–P bond lengths in 8c are somewhat shorter compared with other phosphorus-rich ferrocenophanes,^54,64,65^ indicative for delocalization of the negative charge across the *ansa*-bridge comparable to other cyclic triphosphenium ions.^24,26,27^8c was investigated computationally to get further insight into its bonding situation. As expected, the HOMO is localized mainly (72%) at the central phosphorus and polarized towards the two outer phosphorus atoms, similar to other triphosphenium systems (Fig. S7 in the ESI†). In agreement with the shorter P–P bond lengths second order perturbation analysis on NBO basis revealed backdonation (∼60 kcal mol^−1^) from the central phosphorus atom (Table S7 in the ESI†).

## Conclusion

To conclude, we demonstrated P^+^ transfer from and to a transient phosphenium ion. The dication 4^2+^ resulting from P^+^ addition to this phosphenium ion is stable and its salt 4[AlCl_4_]_2_ is storable in the solid state for prolonged time. The latter serves as P^+^ source itself and transforms various bisphosphanes to the corresponding triphosphenium ions, which was demonstrated for known as well as for unprecedented examples. In this context the first triphosphenium ion bearing hydrogen substituents at both phosphonium centers was obtained and a new synthetic pathway was demonstrated *via* simple protonation of a secondary triphosphane. In analogy to the existing class of triphosphenium ions we suggest the name tetraphosphenium ion for species 4^2+^. The latter features the longest structurally characterized P–P bond involving tricoordinate phosphorus atoms reported so far. Moreover, its FIA value suggests Lewis superacidic character. Based on these new insights we aim at transferring P^+^ ions to other cationic or electron deficient molecules and element fragments in future investigations. In terms of bonding situation, the here presented tetraphosphenium dication 4^2+^ is featuring a non-polar central P–P bond, whereas its reactivity (Schemes 1 and 3) involves heterolytic cleavage of the same bond with an alternative description assuming a polar nature of the electron configurations (sextet and octet) at the central phosphorus atoms in 4^2+^ (*cf.*C, Fig. 4). It is well established, that closed-shell divalent phosphorus compounds (*i.e.* PR_2_^+^ and PR_2_^−^) may be regarded as a continuum of species, where depending on the electronic properties of the substituents R the reactivity ranges from nucleophilic to electrophilic.^86,87^ In this picture, the substituents R would be the phosphonio groups of 4^2+^, concurrently allowing configurations with opposite polarity at the adjacent phosphorus atoms.

## Data availability

The datasets supporting this article have been uploaded as part of the ESI.† Crystallographic data for 2, 4[AlCl_4_]_2_ and 8c[BF_4_] have been deposited at the CCDC under 2328571–2328573 and can be obtained from https://www.ccdc.cam.ac.uk/structures.

## Author contributions

The manuscript was written through contributions of all authors. All authors have given approval to the final version of the manuscript.

## Conflicts of interest

There are no conflicts to declare.

## Supplementary Material

SC-OLF-D4SC06823H-s001

SC-OLF-D4SC06823H-s002
