# Supplementary material for: P+ addition and transfer involving a tetraphosphenium ion
Source: Chem Sci. 2024 Nov 25;16(1):139–46. doi: 10.1039/d4sc06823h (PMC11605702; doi:10.1039/d4sc06823h)
Supplement: SC-016-D4SC06823H-s001 [file SC-016-D4SC06823H-s001.pdf]

Supplementary Information (SI) for Chemical Science.
This journal is © The Royal Society of Chemistry 2024

Electronic Supporting Information

P^+ Addition and Transfer involving a Tetraphosphenium Ion

Roman Franz, Máté Bartek, Clemens Bruhn, Zsolt Kelemen* and Rudolf Pietschnig*

Table of Contents

Table of Contents S2
Experimental Procedures S3
Synthetic protocols and characterization S3
X-ray crystallography S5
Molecular structure of 2 in the solid state S6
Computational details S7
P^+ transfer and related reactions S15
NMR spectra of isolated new compounds S18
References S33
XYZ coordinates S34

Experimental Procedures

All reactions were carried out by means of standard Schlenk or glovebox techniques under inert gas atmosphere (argon). Solvents were dried over Na/K alloy before use and were freshly distilled under inert gas. Deuterated solvents for NMR-spectroscopy were dried and stored over molecular sieves. $[Fe(C_5H_4PCl_2)_2]$[1], $[Fe(C_5H_4P(tBu)H)_2]$[2] (**7**), $[Fe(C_5H_4PtBu)_2PH]$[3] (**9**) and TipLi[4] were prepared according to literature procedures while other reagents were used as received without further purification. ^{1}H-, ^{11}B-, ^{13}C-, ^{19}F-, ^{27}Al-, and ^{31}P-NMR-data was recorded on Jeol JNM-ECZL500, Varian VNMRS-500 MHz or MR-400 MHz spectrometers at 25°C. Chemical shifts were referenced to residual protic impurities in the solvent (^{1}H) or the deuterated solvent (^{13}C) and reported relative to external $SiMe_4$ (^{1}H, ^{13}C). NMR Spectra of heteronuclei were referenced using the Ξ-scale following IUPAC recommendations with $BF_3{\cdot}Et_2O$ (^{11}B), $CFCl_3$ (^{19}F), $Al(NO_3)_2$ (^{27}Al) or H_3PO_4 (85%) (^{31}P) as secondary references.[5] ESI-HR or APCI-DIP-HR mass determinations were performed on a Finnigan LCQ Deca (*ThermoQuest*). Elemental analyses were performed with a HEKAtech Euro EA CHNS elemental analyzer. Samples were prepared in a Sn cup and analyzed with added V_2O_5.
Crystallographic measurements were carried out on a *Stoe* IPDS2 or a *Stoe* StadiVari diffractometer with a STOE image plate detector and a Mo-Kα (λ = 0.71073 Å) monochromator or a *Stoe* StadiVari diffractometer with a Pilatus 200K image plate detector and Cu-Kα (λ = 1.54186 Å) radiation. Direct methods were used to solve the measurements and refined by "least-square" cycles (SHELXL-2017).[6] All non-hydrogen atoms were anisotropically refined without restriction. The evaluation of the data sets, as well as the graphical preparation of the structures was carried out using Olex2[7] and Mercury.[8] Details of the structure determinations and refinement are summarized in Table S1. The CCDC depositions 2328571-2328573 contain the supplementary crystallographic data for this paper, which can be obtained free of charge via emailing data_request@ccdc.cam.ac.uk, or by contacting The Cambridge Crystallographic Data Centre at 12 Union Road, Cambridge CB2 1EZ, UK; fax: +44 1223 336033.

Synthetic protocols and characterization

Synthesis of 2

To a solution of 1.939 g (5 mmol) **1** in 10 mL THF 20 mL of a freshly prepared TipLi solution (0.75 M in THF) was added dropwise at 0 °C. The mixture was warmed to room temperature and stirred overnight. A suspension of 200 mg lithium aluminum hydride in 10 mL THF was added at room temperature and the solution was stirred for another 30 min, before the excess of lithium aluminum hydride was quenched with water. The organic phase was extracted with diethyl ether. The diethyl ether extract was dried with $MgSO_4$ and evaporated to dryness yielding raw product. The raw product was subjected to flash column separation on silica using a mixture of pentane and methylene chloride as eluent which afforded 64% yield (2.092 g, 3.2 mmol) as yellow crystals.
The following NMR chemical shifts, marked with an asterisk, indicate two distinct signals due to *rac*/*meso* diastereomers.
^{1}H-NMR (400 MHz, C_6D_6): δ 7.13 (s, 4H, aryl-H), 5.67* and 5.64* (d, $^{1}J_{PH}$=226 Hz, 2H, P<u>H</u>), 4.39 (m, 1H, Cp), 4.33 (m, 1H, Cp), 4.28 (m, 1H, Cp), 4.22 (m, 1H, Cp), 4.15 – 4.13 (m, 1H, Cp), 4.13 – 4.09 (m, 2H, Cp), 4.13 – 4.09 (m, 4H, *o*-C<u>H</u>(CH$_3$)$_2$), 4.08 – 4.07 (m, 1H, Cp), 2.76 (sept, $^{3}J_{HH}$=6.9 Hz, 2H, *p*-C<u>H</u>(CH$_3$)$_2$), 1.36* and 1.36* (d, $^{3}J_{HH}$=6.8 Hz, 12H, CH$_3$), 1.23 (d, $^{3}J_{HH}$ = 6.8 Hz, 12H, CH$_3$), 1.21* and 1.20 (d, $^{3}J_{HH}$=6.9 Hz, 12H, CH$_3$). ^{13}C-NMR (101 MHz, C_6D_6): δ 152.9 – 152.6 (m, aryl-C), 149.9 (d, $^{n}J_{CP}$=7.1 Hz, aryl-C), 130.5 – 130.1 (m, aryl-C$_q$), 121.8 (d, $^{3}J_{CP}$=2.3 Hz, m aryl-C), 76.8 (psd, Cp), 76.2 (psd, Cp), 75.57 (psd, Cp), 75.5 (psd, Cp), 75.1 – 74.8 (m, Cp-C$_q$), 72.1 (s, Cp), 72.0 (s, Cp), 71.6 – 71.5 (m, Cp), 71.5 – 71.4 (m, Cp), 34.8 (s, *p*-<u>C</u>H(CH$_3$)), 33.3 – 33.1 (m, *o*-<u>C</u>H(CH$_3$)), 25.1 (s, *p*-CH(<u>C</u>H$_3$)), 24.5* and 24.4* (s, *o*-CH(<u>C</u>H$_3$)), 24.2* and 24.2* (s, *o*-CH(<u>C</u>H$_3$)). ^{31}P{^{1}H}-NMR (202 MHz, C_6D_6): –97.8* and –97.9* (s). MS (APCI-DIP-HR) [*m/z*]: 655.328012 ([M+H]$^+$ 10%), calculated for $[C_{40}H_{57}FeP_2]^+$ = 655.327943. Elemental analysis [%]: calculated: C 73.39; H 8.62, found: C 72.97, H 8.83.

Synthesis of 3

A solution of 650 mg (1 mmol) bisphosphane **2** in 20 mL diethylether was cooled to 0 °C. To the stirred solution 1 mL (4.5 mmol) triethylamine and 0.1 mL (157 mg; 1.1 mmol) trichlorophosphane were added dropwise and the mixture was warmed to room temperature. The solvent was removed under reduced pressure and the remaining residue was extracted with 2x20 mL *n*-pentane. The solid was removed and discarded. The product was crystallized from a concentrated *n*-pentane solution at −20 °C, which afforded 65% yield (469 mg, 0.65 mmol) as orange crystalline material.
^{1}H-NMR (400 MHz, C_6D_6): δ 7.07 (s, 4H, aryl-H), 4.80 (m, 2H, Cp), 4.64 (m, 2H, Cp), 4.48 (br, 4H, *o*-C<u>H</u>(CH$_3$)$_2$), 4.45 (m, 2H, Cp), 4.43 (m, 2H, Cp), 2.87 (m, 2H, *p*-C<u>H</u>(CH$_3$)$_2$), 1.32 (d, $^{3}J_{HH}$=6.7 Hz, 24H, *o*-CH(C<u>H</u>$_3$)$_2$), 1.25 (d, $^{3}J_{HH}$=6.9 Hz,12H, *p*- CH(C<u>H</u>$_3$)$_2$).^{13}C-NMR (101 MHz, C_6D_6): δ 156.2 (s, *p*-aryl-C), 151.5 (s, *o*-aryl-C), 127.7 (m, aryl-C$_q$), 122.8 (s, *m*-aryl-C), 80.1 (m, Cp), 73.6 (m, Cp), 73.3 (m, Cp), 72.6 (m, Cp), 70.3 (m, Cp-C$_q$), 34.6 (s, *p*-<u>C</u>H(CH$_3$)$_2$), 33.3 (m, *o*-<u>C</u>H(CH$_3$)$_2$), 24.8 (s, *p*- CH(C<u>H</u>$_3$)$_2$), 23.9 (m, *o*- CH(C<u>H</u>$_3$)$_2$). ^{31}P-NMR (202 MHz, C_6D_6): δ 91.2 (t, $^{1}J_{PP}$=177 Hz, *P*Cl), –28.1 (d, $^{1}J_{PP}$=177 Hz, <u>*P*</u>Tip).
Elemental analysis [%]: calculated: C 66.81, H 7.57, found: C 66.71, H 7.81.

Synthesis of 4[$AlCl_4$]$_2$

72 mg (0.1 mmol) **3** and 20 mg (0.15 mmol) aluminum(III) chloride were suspended in 1 mL 1,2-difluorobenzene and heated to 60 °C for 4 h in an Young-NMR tube. The product was formed as an orange solid which was separated and washed with 1 mL 1,2-difluorobenzene and 1 mL *n*-pentane. Evaporation of residual solvent results in analytically pure product as a yellow solid 20% yield (18 mg, 0.02 mmol). The product can be recrystallized from a concentrated 1,2-difluorobenzene or methylene chloride solution at room temperature.

^{1}H-NMR (400 MHz, DCM-d2): δ 7.33 (m, 4H, aryl-H), 5.63 (m, 4H, Cp), 5.06 (m, 4H, Cp), 3.68 (m, 4H, *o*-$\mathrm{C\underline{H}(CH_3)_2}$), 2.98 (m, 2H, *p*-$\mathrm{C\underline{H}(CH_3)_2}$), 1.62 (br, 12H, *o*-$\mathrm{CH(C\underline{H}_3)_2}$), 1.46 (br, 12H, *o*-$\mathrm{CH(C\underline{H}_3)_2}$), 1.24 (psd, *J*=6.9 Hz, 12H, *p*-$\mathrm{CH(C\underline{H}_3)_2}$). ^{13}C-NMR (101 MHz, DCM-d2): δ 161.1 (m, *p*-C_{Ph}), 153.9 (pst, J_{CP}=7 Hz, *o*-C_{Ph}), 126.3 (pst, *J*=6 Hz, *m*-C_{Ph}), 125.1 (m, aryl-C_q), 80.0 (m, Cp), 78.9 (m, Cp), 63.5 (m, Cp-C_q), 39.1 (s, *o*-$\mathrm{\underline{C}H(CH_3)_2}$), 35.2 (m, *p*-$\mathrm{\underline{C}H(CH_3)_2}$), 25.5 (br, *o*-$\mathrm{CH(\underline{C}H_3)_2}$), 25.0 (br, *o*-$\mathrm{CH(\underline{C}H_3)_2}$), 23.3 (s, *p*-$\mathrm{CH(\underline{C}H_3)_2}$). ^{27}Al-NMR (130 MHz, DCM-d2): δ 104.1 (FWHM=275 Hz). ^{31}P-NMR (202 MHz, DCM-d2): δ –22.9 (t, $^{1}J_{PP}$=306 Hz, $\underline{P}$Tip), –213.1 (t, $^{1}J_{PP}$=306 Hz, $\underline{P}P_3$). Elemental analysis [%]: calculated: C 45.66, H 5.17, found: C 45.88, H 5.29.

Synthesis of 5[Al_2Cl_7]

10 mg (0.01 mmol) **4**[$AlCl_4$]$_2$ were dissolved in 0.6 mL methylene chloride or chloroform. Quantitative formation of the product was observed within 48 h. Evaporation of the solvent leads to pure product as a yellow amorphous solid.

^{1}H-NMR (400 MHz, DCM-d2): δ 7.23 (m, 4H, aryl-H), 5.54 (m, 2H, Cp), 4.90 (m, 2H, Cp), 4.84 (m, 2H, Cp), 4.63 (m, 2H, Cp), 3.57 (m, 4H, *o*-$\mathrm{C\underline{H}(CH_3)_2}$), 2.96 (m, 2H, *p*-$\mathrm{C\underline{H}(CH_3)_2}$), 1.46 (psd, *J*=6.5 Hz, 12H, *o*-$\mathrm{CH(C\underline{H}_3)_2}$), 1.41 (psd, *J*=6.5 Hz, 12H, *o*-$\mathrm{CH(C\underline{H}_3)_2}$), 1.26 (psd, *J*=6.9 Hz, 12H, *p*-$\mathrm{CH(C\underline{H}_3)_2}$). ^{27}Al-NMR (130 MHz, DCM-d2): δ 103.9 (FWHM=150 Hz). ^{31}P-NMR (202 MHz, DCM-d2): δ 19.5 (dd, $^{1}J_{PP}$=372 Hz, $^{1}J_{PP}$=337 Hz, $\underline{P}$Tip), –39.8 (td, $^{1}J_{PP}$=337 Hz, $^{2}J_{PP}$=108 Hz, P($\underline{P}$Cl)P, –130.7 (td, $^{1}J_{PP}$=372 Hz, $^{2}J_{PP}$=108 Hz, P$\underline{P}$P). Elemental analysis [%]: calculated: C 45.66, H 5.17, found: C 45.62, H 5.18.

Synthesis of 6[$AlCl_4$]

To a suspension of 7 mg (0.05 mmol) aluminum(III) chloride 0.5 mL methylene chloride a mixture of 36 mg (0.05 mmol) **3** and 14 mg (0.05 mmol) triphenylphosphine in 0.5 mL methylene chloride was added at room temperature. Evaporation of the solvent results in analytically pure product as an amorphous orange solid in quantitative yield (57 mg, 0.05 mmol).

^{1}H-NMR (400 MHz, DCM-d2): δ 7.68 – 7.55 (m, 4H, aryl-H), 7.44 – 7.22 (m, 15H, -PPh_3), 4.69 (m, 2H, Cp), 4.49 (m, 4H, Cp), 4.45 (m, 2H, Cp), 4.06 – 3.95 (m, 2H, *o*-$\mathrm{C\underline{H}(CH_3)_2}$), 3.33 (dt, $^{3}J_{HH}$=12.4 Hz, 2H, *o*-$\mathrm{C\underline{H}(CH_3)_2}$), 2.94 (m, 2H, *p*-$\mathrm{C\underline{H}(CH_3)_2}$), 1.34 – 1.28 (m, 24H, *o*-$\mathrm{CH(C\underline{H}_3)_2}$), 0.87 (d, $^{3}J_{HH}$=6.9 Hz, 6H, *p*-$\mathrm{CH(C\underline{H}_3)_2}$), 0.75 (d, $^{3}J_{HH}$=6.7 Hz, 6H, *p*-$\mathrm{CH(C\underline{H}_3)_2}$). ^{13}C-NMR (101 MHz, DCM-d2): δ 155.9 (m, *o*-C_{Ph}), 155.2 (m, *o*-C_{Ph}), 153.3 (s, *p*-C_{Ph}), 134.9 (m (br), C_{Ph}), 134.4 (m (br), C_{Ph}), 134.1 (m, C_{Ph}), 130.1 (m, C_{Ph}), 130.0 (m, C_{Ph}), 129.1 (m (br), C_{Ph}), 129.0 (m (br), C_{Ph}), 125.6 (m, C_{Ph}), 124.6 (s, C_{Ph}), 122.8 (pst, C_{Ph}), 79.2 (m, Cp), 77.4 (pst, Cp), 74.0 (pst, Cp), 73.3 (pst, Cp), 69.8 (m, Cp-C_q), 34.7 (s, *p*-$\mathrm{\underline{C}H(CH_3)_2}$), 34.4 (m, *o*-$\mathrm{\underline{C}H(CH_3)_2}$), 32.7 (m, *o*-$\mathrm{\underline{C}H(CH_3)_2}$), 27.1 (s, *p*-$\mathrm{CH(\underline{C}H_3)_2}$), 26.5 (s, *p*-$\mathrm{CH(\underline{C}H_3)_2}$), 25.6 (psd, *o*-$\mathrm{CH(\underline{C}H_3)_2}$), 24.0 (s, *o*-$\mathrm{CH(\underline{C}H_3)_2}$), 23.9 (s, *o*-$\mathrm{CH(\underline{C}H_3)_2}$), 22.4 (s, *o*-$\mathrm{CH(\underline{C}H_3)_2}$). ^{27}Al-NMR (130 MHz, DCM-d2): δ 103.8 (FWHM=51 Hz). ^{31}P-NMR (202 MHz, THF-d8): δ 22.3 (dt, $^{1}J_{PP}$=379 Hz, $^{2}J_{PP}$=72 Hz, $\underline{P}Ph_3$), –5.2 (dt, $^{1}J_{PP}$=379 Hz, $^{2}J_{PP}$=174 Hz, $\underline{P}$-PPh_3), –43.2 (dd, $^{1}J_{PP}$=174 Hz, $^{2}J_{PP}$=72 Hz, $\underline{P}$Tip). Elemental analysis [%]: calculated: C 62.55, H 6.15, found: C 62.80, H 6.29.

Synthesis of 8c[BF_4]

To a solution of 196 mg (0.5 mmol) secondary triphosphane **9** in 20 mL diethylether 1.25 mL (0.5 mmol) of a freshly prepared $HBF_4\cdot Et_2O$ solution (0.4 M in Et_2O) was added at room temperature. The product was formed as a yellow solid within 10 min. The solvent was removed under reduced pressure. The remaining residue was washed with 2x20 mL *n*-pentane and extracted with 2x10 mL dichloromethane. The solid was removed and discarded. The product was crystallized from a concentrated toluene solution at −20 °C, which afforded 65% yield (156 mg, 0.33 mmol).

^{1}H-NMR (400 MHz, DCM-d2): δ 7.07 (m, $^{1}J_{PH}$=467.5 Hz, 2H, PH), 5.18 (m, 2H, Cp), 4.79 (m, 4H, Cp), 4.57 (m, 2H, Cp), 1.33 (d, $^{3}J_{PH}$=18.8 Hz, 18H, *t*Bu CH_3). ^{13}C-NMR (101 MHz, DCM-d2): δ 77.9 (m, Cp), 76.2 (m, Cp), 73.6 (m, Cp), 72.2 (m, Cp), 69.7 (m, Cp C_{ipso}), 34.1 – 32.7 (m, *t*Bu C_q), 27.1 (m, *t*Bu CH_3). ^{11}B-NMR (160 MHz, DCM-d2): δ 0.5 (br). ^{19}F-NMR (470 MHz, DCM-d2): δ 149.4 (q, $^{1}J_{BF}$=2 Hz). ^{31}P-NMR (202 MHz, DCM-d2): δ 45.2 (dd, $^{1}J_{PP}$=456 Hz, $^{1}J_{PH}$=468 Hz, $\underline{P}$H*t*Bu), –195.2 (t, $^{1}J_{PP}$=456 Hz, P$\underline{P}$P). MS (ESI-HR) [*m/z*]: 393.074792 ([M–BF_4]$^+$ 100%), calculated for [$C_{18}H_{28}FeP_3$]$^+$ = 393.074779. Elemental analysis [%]: calculated: C 45.04, H 5.88; found: C 45.19, H 5.92.

X-ray crystallography

Table S1: Summary of structure determinations and refinements for **2**, **4**[$AlCl_4$]$_2$ and **8c**[BF_4].

	**2**	**4**[$AlCl_4$]$_2$	**8c**[BF_4]
CCDC code	2328571	2328572	2328573
Empirical formula	$C_{40}H_{56}FeP_2$	$C_{40}H_{54}Al_2Cl_8FeP_4$ 2(CH_2Cl_2)	$C_{18}H_{28}BF_4FeP_3$
Formula weight [g/mol]	654.63	1221.97	479.97
Crystal system	monoclinic	triclinic	monoclinic
Space group	$P2_1$/n	$P\bar{1}$	$P2_1$/n
Unit cell dimensions:			
a [Å]	6.0446(3)	9.2572(9)	10.9377(5)
b [Å]	34.1672(12)	16.6386(16)	15.3369(5)
c [Å]	17.5241(8)	18.6790(18)	13.4763(6)
α [°]	90	96.852(8)	90
β [°]	96.228(4)	99.363(8)	107.121(3)
γ [°]	90	94.470(8)	90
Volume [Å^3]	3597.8(3)	2804.5(5)	2160.47(16)
Z	4	2	4
Calculated density [g/cm^3]	1.209	1.447	1.476
Absorption coefficient *μ* [mm^{-1}]	0.534	9.032	0.955
F(000)	1408	1252	992
Crystal size [mm]	0.20 × 0.09 × 0.02	0.35 × 0.13 × 0.02	0.34 × 0.16 × 0.02
Radiation and *λ* [Å]	Mo $K_α$ (λ = 0.71073)	Cu $K_α$ (λ = 1.54186)	Mo $K_α$ (λ = 0.71073)
Θ-Range for data collection [°]	2.38 – 51.38	5.38 – 143.09	4.13 – 53.54
Index ranges	$-7 \leq h \leq 6$	$-11 \leq h \leq 5$	$-13 \leq h \leq 13$
	$-41 \leq k \leq 36$	$-20 \leq k \leq 19$	$-19 \leq k \leq 17$
	$-21 \leq l \leq 21$	$-22 \leq l \leq 22$	$-16 \leq l \leq 17$
Refl. collected/unique	15032/6776	22080/10357	14617/4576
Data/restraints/parameters	6776/0/413	10357/0/562	4576/0/258
Goodness-of-fit on F^2	1.034	1.044	1.048
Final *R* indices [I>2$\delta(I)$]/ [wR_2]	0.0731/0.1798	0.1377/0.3584	0.0283/ 0.0689
R indices (all data)/ [wR_2]	0.1228/0.2146	0.1953/0.4064	0.0350/ 0.0724
Largest difference peak/hole [e Å^{-3}]	0.63/-0.84	2.26/-1.58	0.36/-0.27

Molecular structure of 2 in the solid state

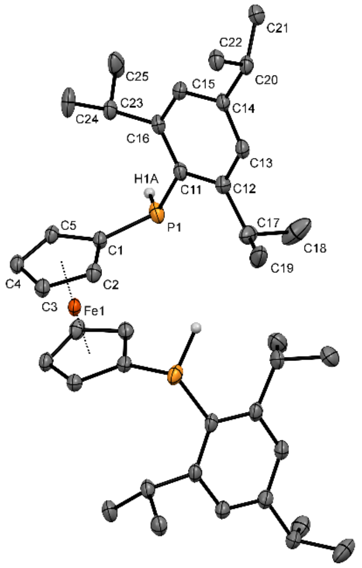

Figure S1: Molecular structures of **2** in the solid state. Ellipsoids are shown at 30% probability and all except the phosphorus-bonded hydrogen atoms are omitted. A disorder of the phosphorus-bonded hydrogen atoms over two positions is also not shown for clarity. Selected bond lengths in **2**: 1.21(13) Å (P1-H1A), 1.39(12) Å (P2-H2A).

Computational details

All DFT calculations were carried out with the Gaussian 16 suite of programs.[9] In our previous studies, we showed that the functional ωB97X-D describes properly similar systems.[10, 11] Therefore, geometry optimizations were performed at the ωB97X-D/def2-TZVP level of theory, with the sole exception of the mechanistic studies (formation of **4**$^{2+}$ and „P^{+}“ transfer reaction discussed in Scheme 3), where a smaller basis set (def2-SVP) was applied for the optimization. Harmonic vibrational analysis was obtained at the same level, and in the case of a minima, all eigenvalues of the Hessian matrix were positive. In order to consider the effect of solvents (in case of mechanistic study and FIA calculations), the PCM model was applied. Since 1,2-difluorobenzene was not defined in the G16 program package, the less polar THF solvent was used for these calculations. The energies of molecular ground states were then recomputed applying ωB97X-D/def2-TZVP and PCM solvent model. Gibbs free energies (at 298 K) were calculated using these electronic energies with TΔS contribution derived from frequency calculations at the ωB97X-D/def2-TZVP (or ωB97X-D/def2-SVP in case of systems described in Scheme 3 and Scheme S1) level of theory. For the determination of bond critical points, Mayer indices, analysis of the Fukui function and for orbital contribution analysis the Multiwfn program was used.[12] Atomic basin analysis was performed using a medium sized grid (with a grid spacing of 0.1 Bohr) in Multiwfn. The basin overlap matrix was computed using a mixed grid. For visualization of the molecular orbitals IQmol program was used.[13] For NBO calculations NBO 7.0 was used.[14]

Table S2: Derived bond descriptors from the values of representative real space functions at the corresponding bond critical points in **4**$^{2+}$ and **5**$^{+}$ [au].

Bader suggested to divide interactions in a dichotomous manner based on the sign of the Laplacian in the bond critical point (∇_b).[15] The symmetry of the valance shell charge concentration (VSCC) can be examined by plotting ∇(r) along the corresponding bond path. A large, negative Laplacian with symmetric VSCC is characteristic of covalent interactions. Further distinctions can be made[16] from the kinetic energy density (G_b) and the potential energy density (V_b) at the BCP, which are connected to the value of the Laplacian by the Virial theorems. The sum of V_b and G_b gives the total energy density H_b. The adimensional ratios $G_b/\rho_b < 1$, $H_b/\rho_b < 0$, $|V_b|/G_b > 2$ have been attributed to shared shell, covalent interactions.

	**4**$^{2+}$			**5**$^{+}$		
	**P1-P2**	**P2-P3**	**P1-P3**	**P1-P2**	**P2-P3**	**P1-P3**
$sgn\ \nabla_b^2$	-	-	-	-		-
G_b/ρ_b	0.25	0.35	0.25	0.20		0.20
$\lvert V_b\rvert/G_b$	3.32	2.24	3.32	3.82		3.82
H_b/ρ_b	-0.57	-0.43	-0.57	-0.55		-0.55

Table S3: Mayer bond indices and values of representative real space functions at the corresponding bond critical points in **4**$^{2+}$ and **5**$^{+}$ [au].

	**4**$^{2+}$			**5**$^{+}$		
	**P1-P2**	**P2-P3**	**P1-P3**	**P1-P2**	**P2-P3**	**P1-P3**
Mayer bond index	1.00	0.92	1.00	0.97	0.26	0.97
Electron density in BCP	0.122	0.093	0.122	0.123	-	0.123
$\nabla^2\rho_b$	-0.158	-0.0314	-0.158	-0.174	-	-0.174
G_b	0.03	0.0323	0.0300	0.0240	-	0.0240
V_b	-0.0996	-0.0725	-0.0996	-0.0916	-	-0.0916
H_b	-0.0695	-0.004	-.00695	-0.0676	-	-0.0676

Table S4: Delocalization indices (derived from QTAIM analysis) for $\mathbf{4}^{2+}$ and $\mathbf{5}^{+}$

		$\mathbf{4}^{2+}$				$\mathbf{5}^{+}$		
	**P1**	**P2**	**P3**	**P4**	**P1**	**P2**	**P3**	**P4**
**P1**	4.24	0.96	0.96	0.10	4.27	0.91	1.05	0.17
**P2**	0.96	3.45	0.93	0.96	0.91	3.51	0.28	0.91
**P3**	0.96	0.93	3.45	0.96	1.05	0.28	3.17	1.05
**P4**	0.10	0.96	0.96	4.24	0.17	0.91	1.05	4.28

Table S5: Partial natural charges, and QTAIM charges of the phosphorus atoms in $\mathbf{4}^{2+}$.

	$\mathbf{4}^{2+}$			
	**P1**	**P2**	**P3**	**P4**
Natural charges	1.00	0.21	0.21	1.00
QTAIM charges	1.41	0.24	0.24	1.41

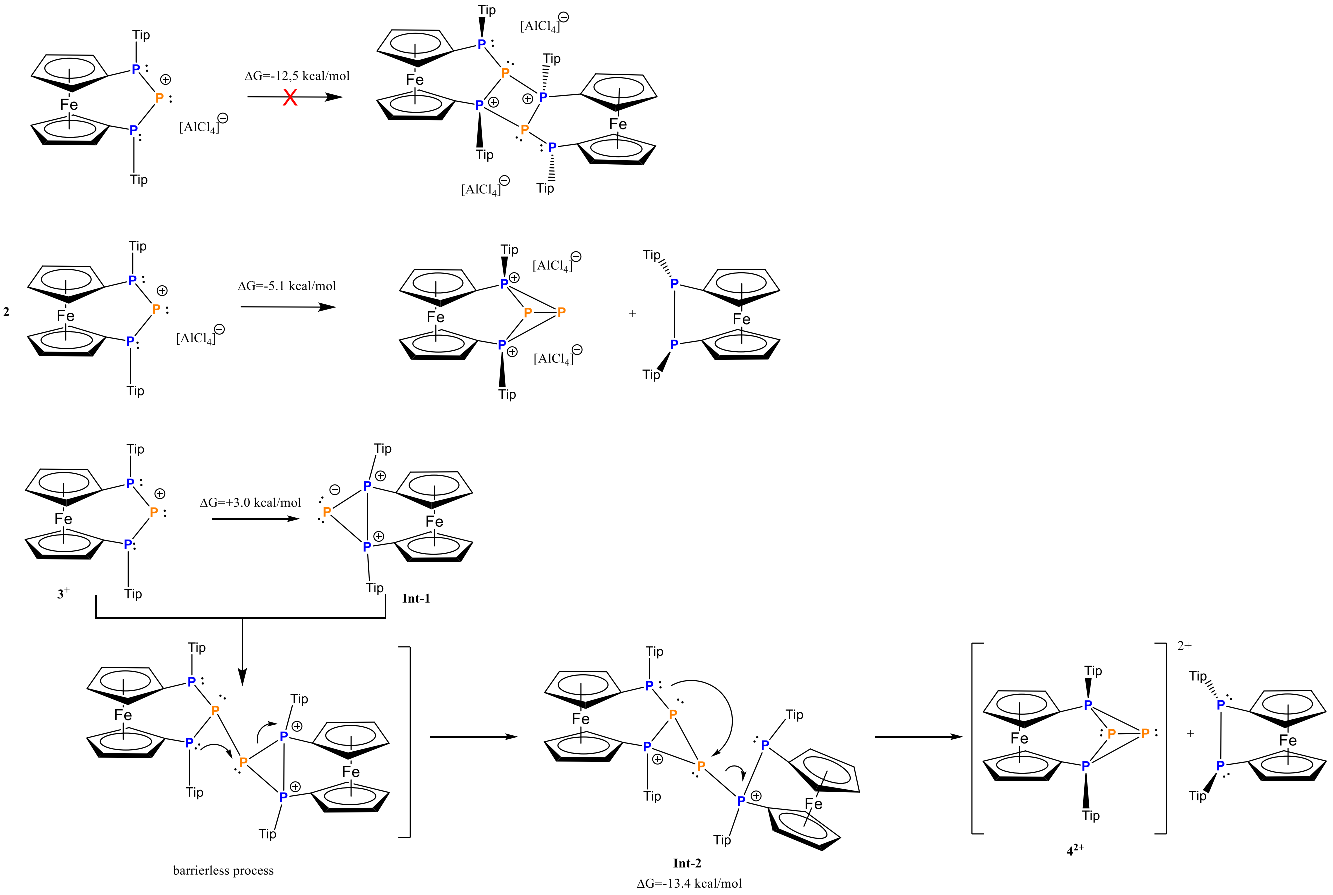

Scheme S1 Proposed mechanism of the formation of $\mathbf{4}^{2+}$. During our DFT investigations, we successfully optimized the structure of **Int-1**. The relative stability of **Int-1** is similar to that of the starting $\mathbf{3}^{+}$ (ΔG=+3 kcal/mol) and the central phosphorus in **Int-1** is inversely polarized compared to $\mathbf{3}^{+}$, which may allow P-P bond formation between the two compounds without significant barrier. During the geometry optimization of the proposed P-P bonded complex of **3+** and **Int-1**, we obtained **Int-2**, which is more stable by 13.4 kcal/mol than the van der Waals complex of $\mathbf{3}^{+}$ and **Int-1**. The next step involves the elimination of diphosphane. It is important to note that a direct comparison of the different processes in terms of Gibbs free energy is limited due to the different overall charges of the investigated systems (cationic systems with or without counter ions). (Geometry optimizations were performed at ωB97X-D/def2-SVP. The energies of molecular ground states were then recomputed applying ωB97X-D/def2-TZVP and PCM solvent model. Gibbs free energies (at 298 K) were calculated using these electronic energies with TΔS contribution derived from frequency calculations at the ωB97X-D/def2-SVP level of theory.)

Table S6: Comparison of the FIA values (in kcal/mol) of **4**$^{2+}$ with different dicationic systems reported by Dielmann[15] (recomputed with Me substituents at the imidazolium units), Weigand&Burford[17] and Alder.[18]

Compound	FIA_{gas}	$FIA_{PCM=THF}$
**4**$^{2+}$	239.0	87.2
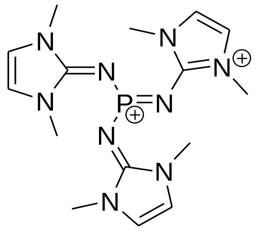	246.0	87.9
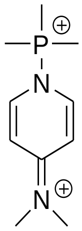	245.2	68.5
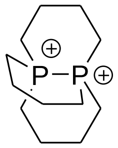	243.2	64.1

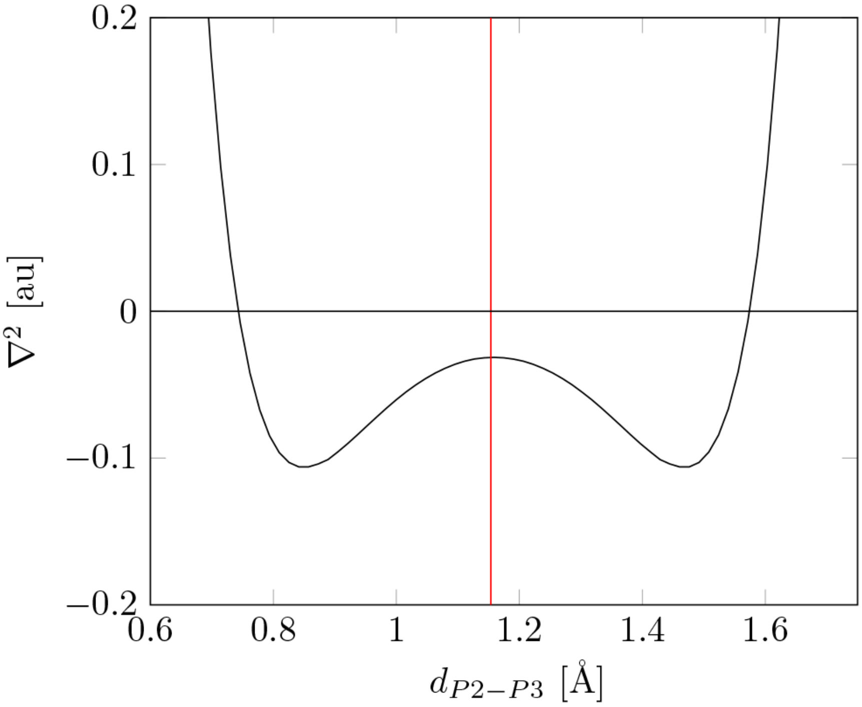

Figure S2: Value of the Laplacian along the P2-P3 bond path in **4**$^{2+}$. The position of the BCP is indicated as a red line.

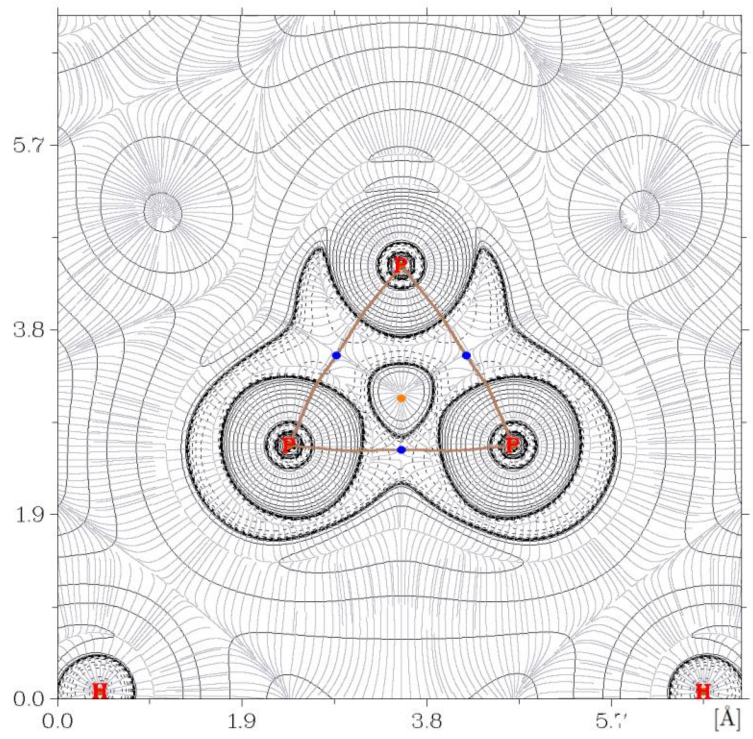

Figure S3: The Laplacian gradient (grey) and contour (dotted) plot for $\mathbf{4}^{2+}$ in the P1-P2-P3 plane. Bond critical points are indicated as blue, and ring critical points with orange dots.

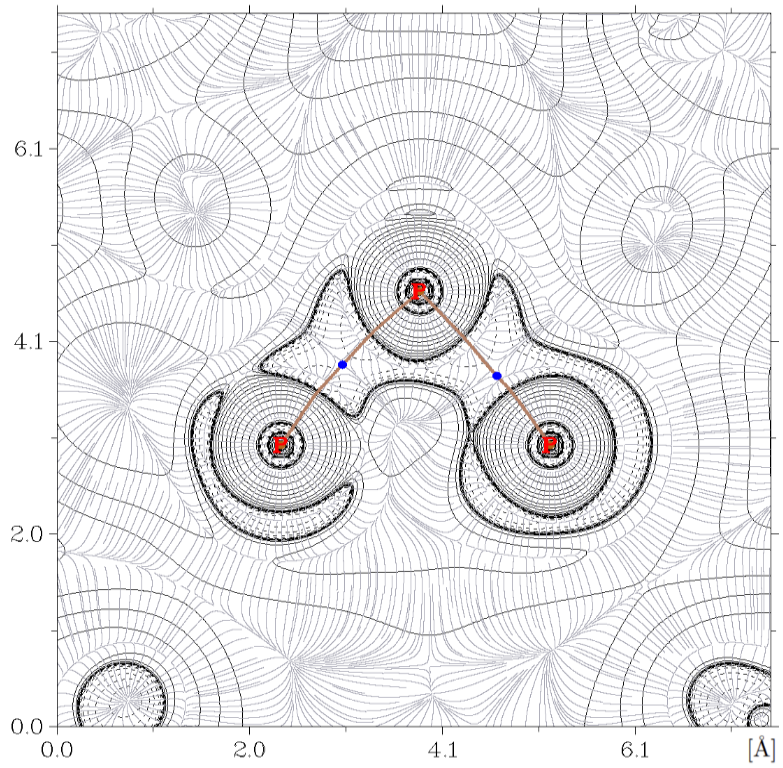

Figure S4: The Laplacian gradient (grey) and contour (dotted) plot for $\mathbf{5}^{+}$ in the P1-P2-P3 plane. Bond critical points are indicated as blue dots

Table S7: Orbital contribution of each phosphorus atom to the HOMO, and natural charges of **8a, 8b** and **8c**. Backdonation was computed from the second order perturbation theory analysis on the Fock matrix by the sum of the corresponding E(2) energies involving the lone pairs of the central phosphorus atoms and the antibonding orbitals of neighboring phosphorus atoms.

	**8a**			**8b**			**8c**		
	**P1**	**P2**	**P3**	**P1**	**P2**	**P3**	**P1**	**P2**	**P3**
Natural Charge	1.26	-0.30	1.26	1.31	-0.35	1.30	1.02	-0.34	1.02
Atomic contribution to the HOMO [%]	6.5	70.5	8.6	5.99	70.5	6.4	5.9	72.1	7.3
Back donation [kcal/mol]	62.0			56.7			58.6		

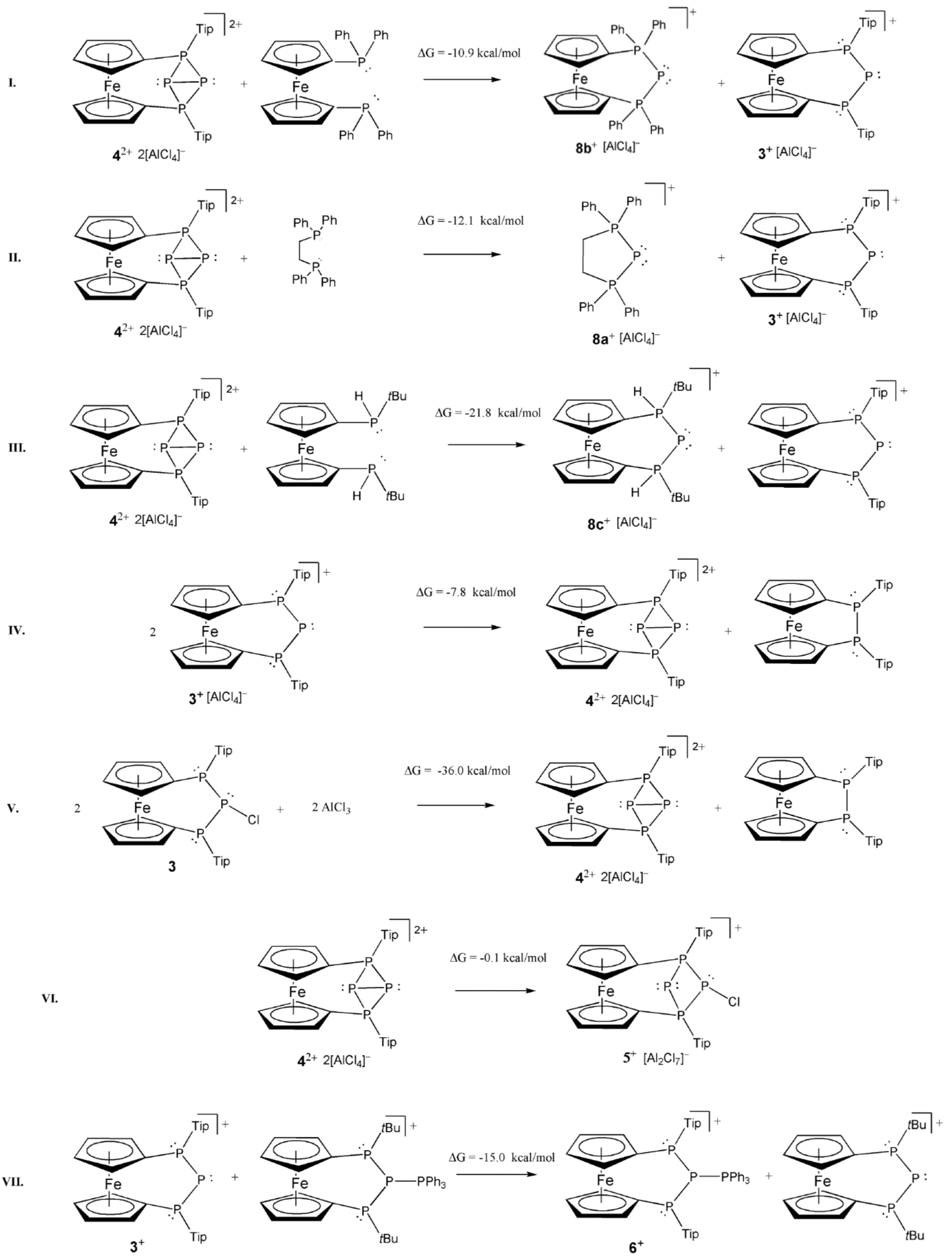

Scheme S2: Calculated reactions and their reaction Gibbs free energies. (Geometry optimizations were performed at ωB97X-D/def2-TZVP. The energies of molecular ground states were then recomputed applying the PCM solvent model. Gibbs free energies (at 298 K) were calculated using these electronic energies with TΔS contribution derived from frequency calculations at the ωB97X-D/def2-TZVP level of theory.)

Table S8: Comparison of the reaction Gibbs free energies derived from the gas phase calculations ($\Delta G_{gas\ phase}$) and after considering solvent effect ($\Delta G_{solvent}$). (Geometry optimizations were performed at ωB97X-D/def2-TZVP. The energies of molecular ground states were then recomputed applying the PCM solvent model. Gibbs free energies (at 298 K) were calculated using these electronic energies with TΔS contribution derived from frequency calculations at the ωB97X-D/def2-TZVP level of theory.)

	$\Delta G_{gas\ phase}$ [kcal/mol]	$\Delta G_{solv.corr}$ [kcal/mol]
**I.**	-19.3	-10.9
**II.**	-22.6	-12.1
**III.**	-34.8	-21.8
**IV.**	1.8	-7.8
**V.**	-13.2	-36.0
**VI.**	-22.7	-0.1
**VII.**	-13.7	-15.0

Further discussion of the bonding situation of 4^{2+}

During the NBO analysis discussed in this section a local subset was defined and used, where only the P_4 unit and the directly connected carbon atoms were considered. These calculations were carried out at the ωB97X-D/def2-SVP// ωB97X-D/def2-TZVP level of theory. The second order perturbation analysis of the Fock matrix (on NBO basis) revealed strong donor-acceptor interactions between the P-P bonds and neighboring antibonding orbitals. The P2-P3 bond exhibits a stronger donating ability (22.0 kcal/mol), than the others (P1-P2: 11.4 kcal/mol, P1-P3: 12.3 kcal/mol). This effect can also be observed in NHO Directionality analysis, where the deviation of the hybrid direction from the line of centers between the two nuclei (deviation angle [°]) is indeed larger (P1-P2, P1-P3: 16.5° P2-P3: 19.9°). The direction of this donor-acceptor interaction is mainly towards the antibonding orbitals between the pillar atoms of the cage and the *ipso*-carbon atoms, which aligns with the shapes of the Kohn-Sham orbitals computed. Additionally, natural resonance theory (NRT) was utilized to compute the natural bond orders, and resonance structures. The natural bond order reveals that the bonds between the adjoining phosphorus atoms of the cage is mainly (90%) covalent. The central bond can be considered purely covalent (~100%). NRT analysis showed that only one leading resonance structure exist with 70%, which is identical with the Lewis structure depicted in Scheme 1.

To highlight the reactive sites of $\mathbf{4}^{2+}$, the orbital-weighed Fukui function was used to compute the dual descriptor (Δf_w) values of possible sites susceptible to a nucleophilic attack. Several maxima of the Δf_w have been identified on the corresponding isosurface near the phosphorus cage. Based on these values, it can be concluded that $\mathbf{4}^{2+}$ is prone to attack on both sides of the central P atoms.

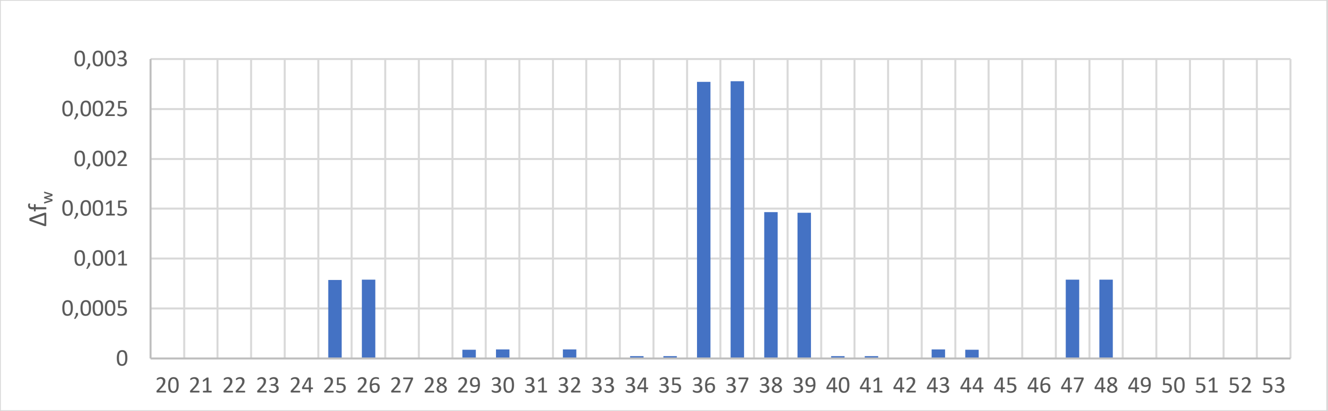

Figure S5: Δf_w values for the identified maximas on the Fukui function for $\mathbf{4}^{2+}$ (ωB97X-D/def2-TZVP).

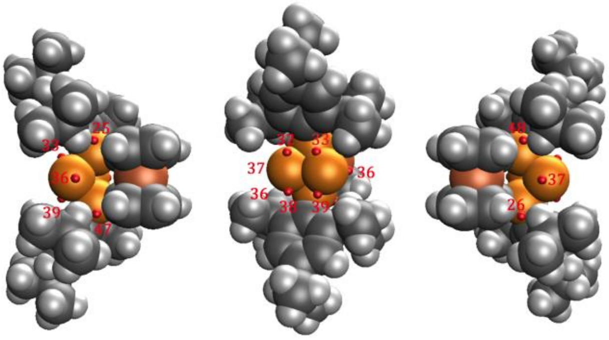

Figure S6: Maxima sites (Fukui function) represented on the space filling scheme of $\mathbf{4^{2+}}$ (ωB97X-D/def2-TZVP).

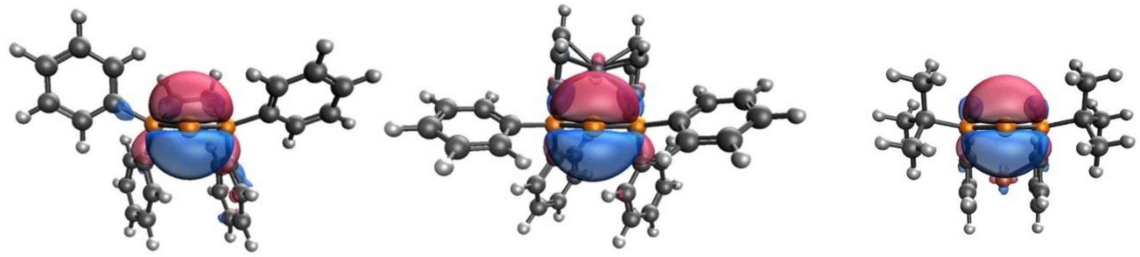

8a⁺ HOMO ε = - 10.80 eV | **8b⁺** HOMO ε = - 10.46 eV | **8c⁺** HOMO ε = - 11.03 eV

Figure S7: The HOMO of $\mathbf{8a^{+}}$, $\mathbf{8b^{+}}$ and $\mathbf{8c^{+}}$ at ωB97X-D/def2-TZVP (isovalue=0.05).

P^+ transfer and related reactions

Products and by-products of the P^+ transfer reactions have been identified on an NMR scale based on their characteristic spectra.

<u>*General procedure P^+ transfer*</u>

To a solution of 5 mg **4**[$AlCl_4$]$_2$ in 0.5 ml dichloromethane 20 mg of neat dppe were added at room temperature in an NMR tube. The NMR tube was closed and the mixture gently shaken three times. The formation of literature known **8a** is confirmed by ^{31}P NMR spectroscopy.[19] Replacing dppe by dppf or **7** affords **8b** and **8c** in a similar manner, out of which **8b** has been described before.[20] A superior procedure to **8c** has been described in the synthetic protocol section earlier in this supporting information.

$^{31}P\{^1H\}$-NMR spectrum of **8a**[$AlCl_4$] obtained via P^+ transfer (signals marked with asterisks refer to the byproduct as further outlined below):

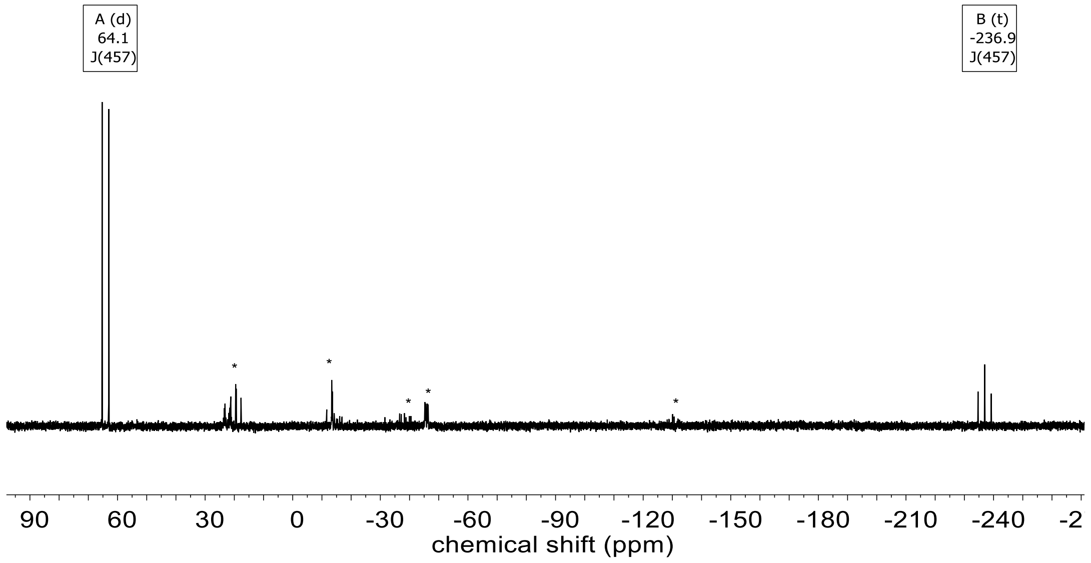

$^{31}P\{^1H\}$-NMR spectrum of **8b**[$AlCl_4$] obtained via P^+ transfer (signals marked with asterisks refer to the byproduct as further outlined below):

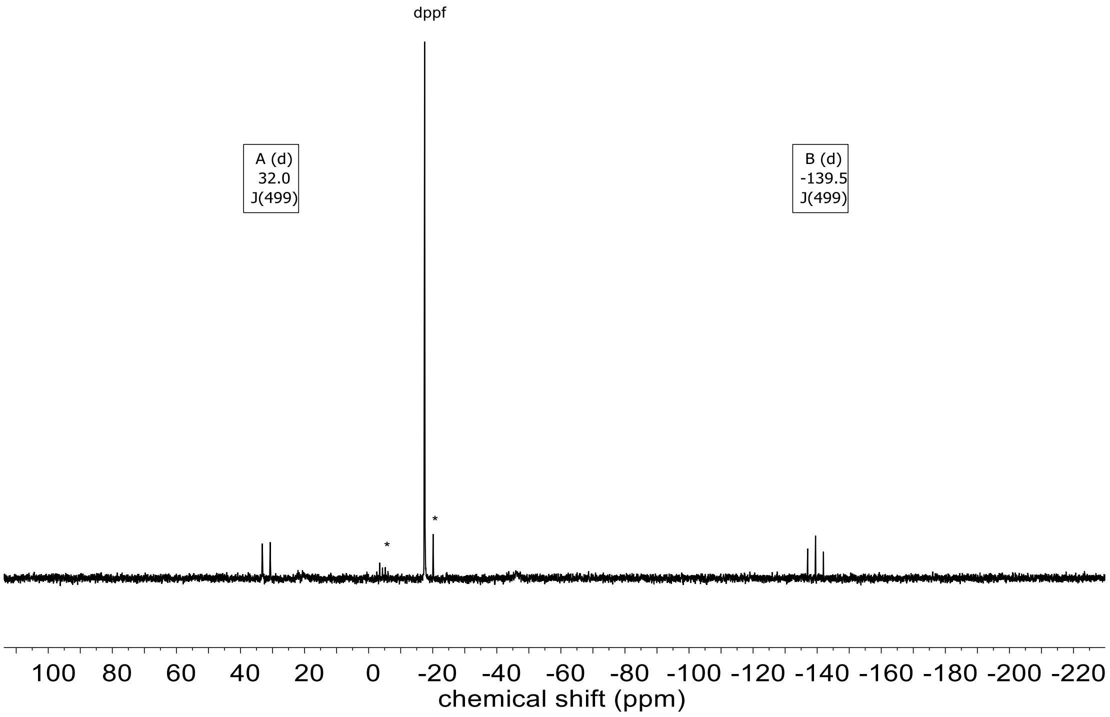

$^{31}P\{^{1}H\}$-NMR of **8c**[AlCl$_4$] obtained via P$^+$ transfer (signals marked with asterisks refer to the byproduct as further outlined below):

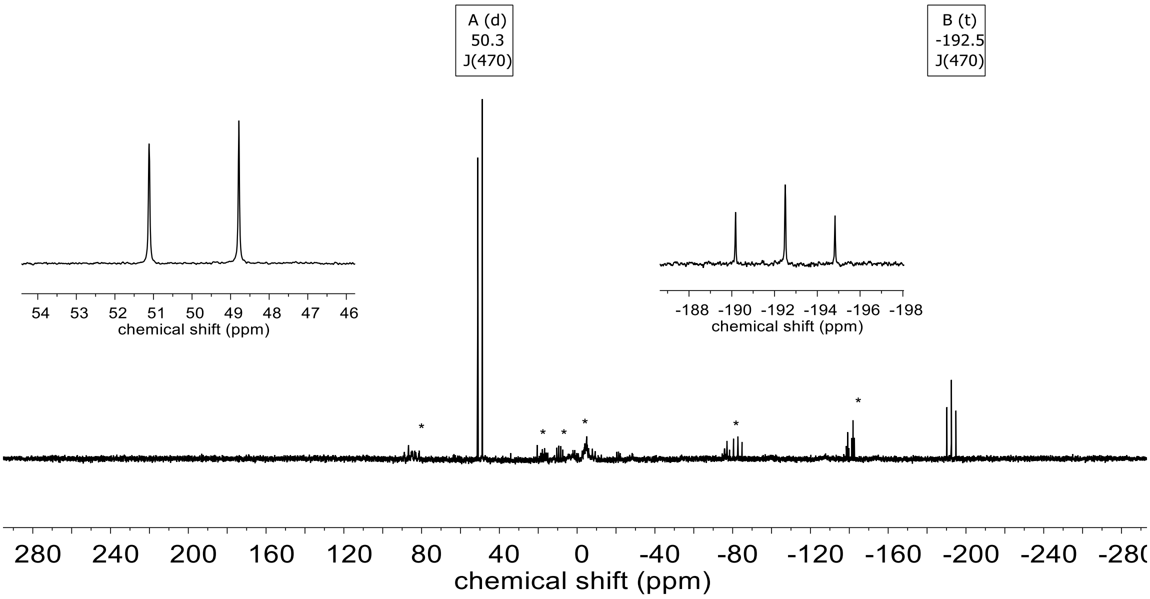

Targeted formation of the by-product observed during P$^+$ transfer

As outlined in the main manuscript and summarized below in Scheme S3, the initial side product of the P$^+$ transfer is the free phosphenium ion **3**$^+$, which is not stable and reacts further, e.g. with the bisphosphanes added as P$^+$ acceptors. We were able to identify the follow-up product of the free phosphenium ion **3**$^+$ as the corresponding mono phosphane adduct of the phosphenium ion (Scheme S3 and Figure S8 and S9) comparable to the PPh$_3$ adduct **6**$^+$ (see Scheme 1). Furthermore, we have investigated the targeted formation of the by-product via *in situ* phosphenium ion generation starting from **3** with AlCl$_3$ in the presence of dppf and dppe (Scheme S3), which leads to the formation of the same products, now as main products, in support of the above-mentioned assignment for the by-product observed during P$^+$ transfer.

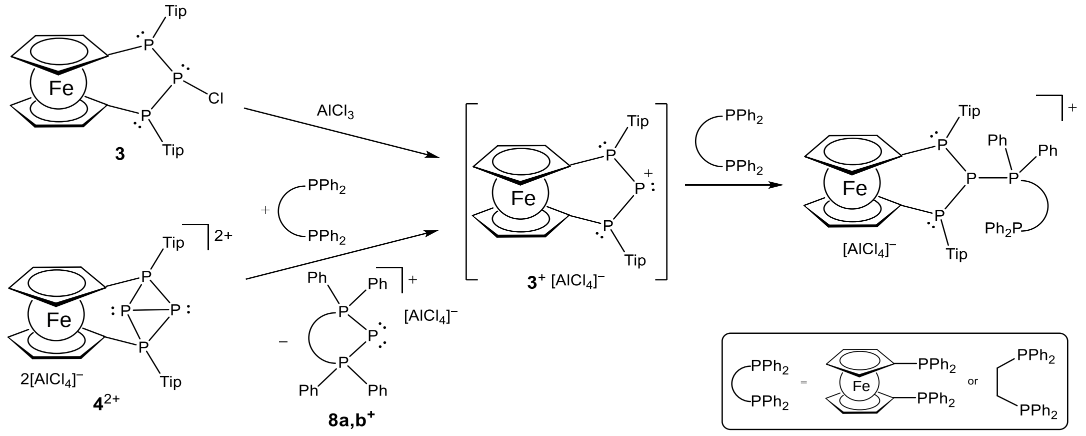

Scheme S3: Illustration of the by-product formed during P$^+$-transfer from **4**$^{2+}$ (lower path) *vs.* targeted formation of this by product via the *in situ* formed phosphenium ion (**3**$^+$) obtained by chloride abstraction starting from **3** (higher path) with dppf or dppe.

$^{31}P\{^1H\}$-NMR spectrum of *in situ* reaction of phosphenium ion (**3**$^+$) with dppe in CD_2Cl_2 at room temperature.

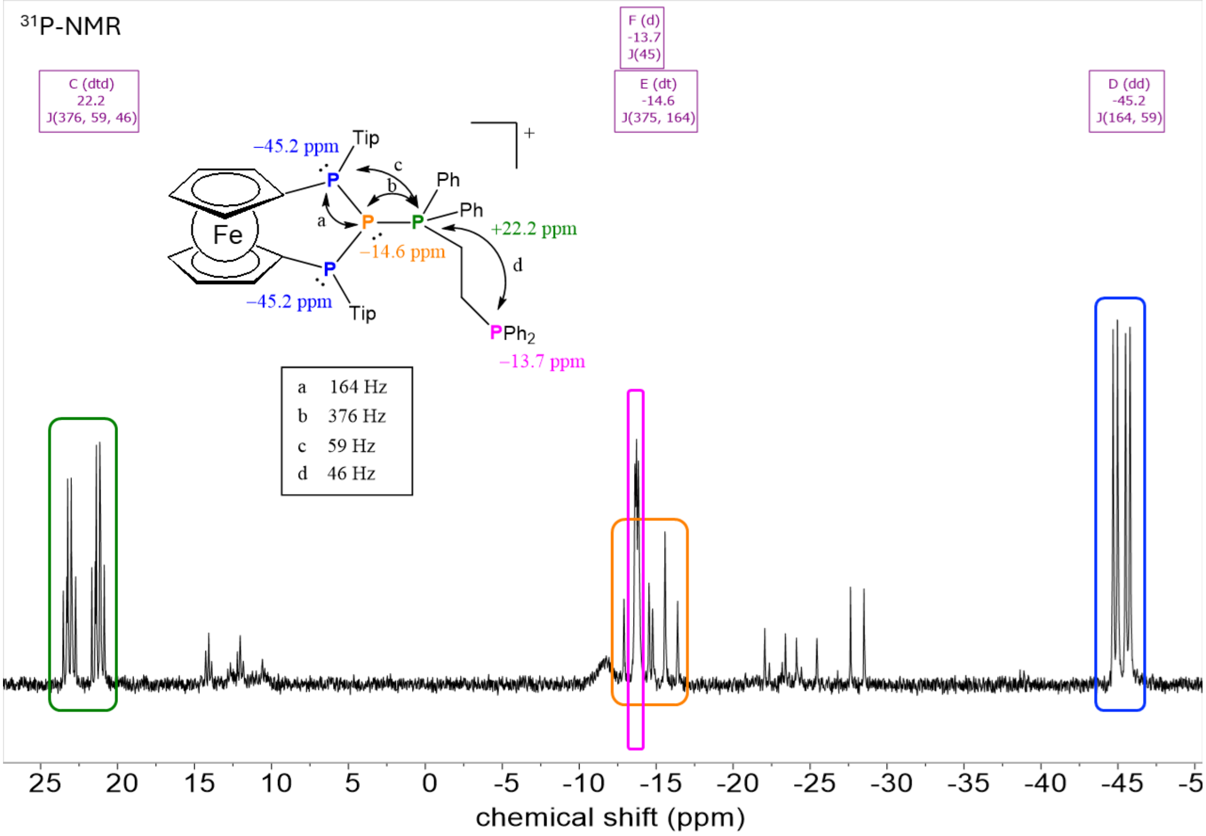

$^{31}P\{^1H\}$-NMR spectrum of *in situ* reaction of phosphenium ion (**3**$^+$) with dppf in CD_2Cl_2 at room temperature:

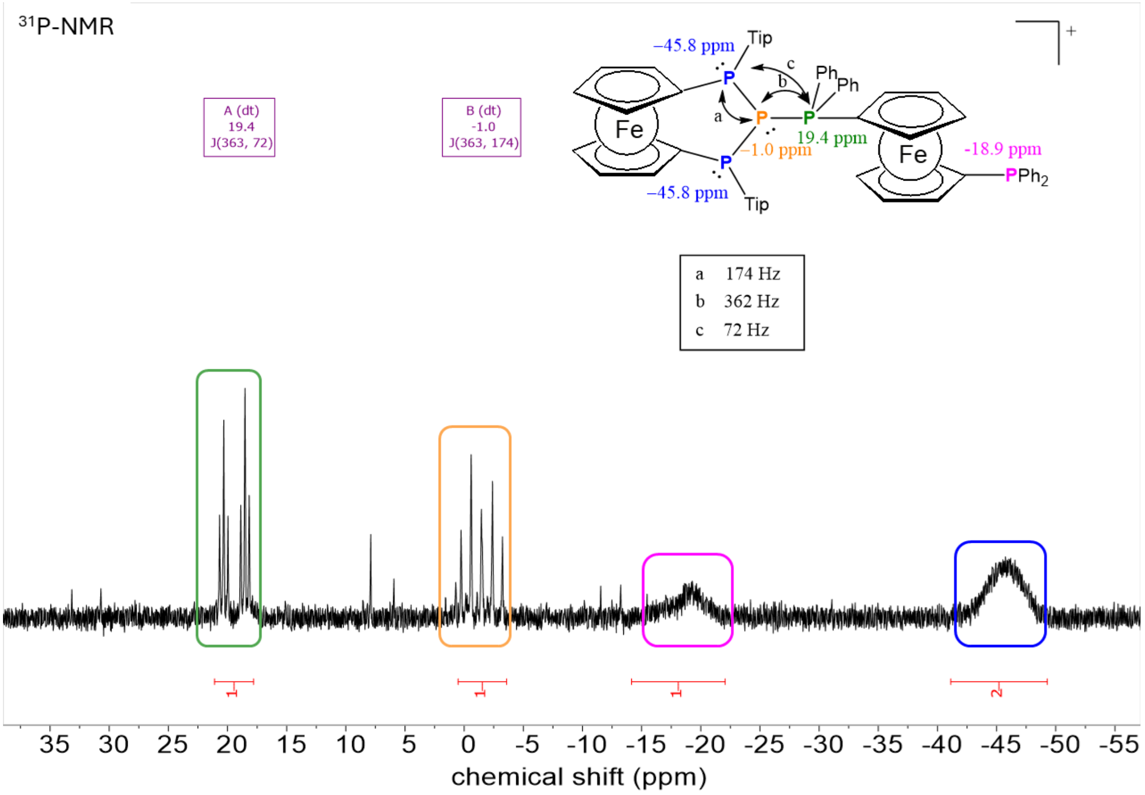

NMR spectra of isolated new compounds

On the following pages the NMR-spectra of compounds **2**, **3**, **4**[$AlCl_4$]$_2$, **5**[Al_2Cl_7], **6**[$AlCl_4$] and **8c**[BF_4] are depicted and sorted by compound:

NMR-spectra of **2**:
$^{31}P\{^{1}H\}$-NMR

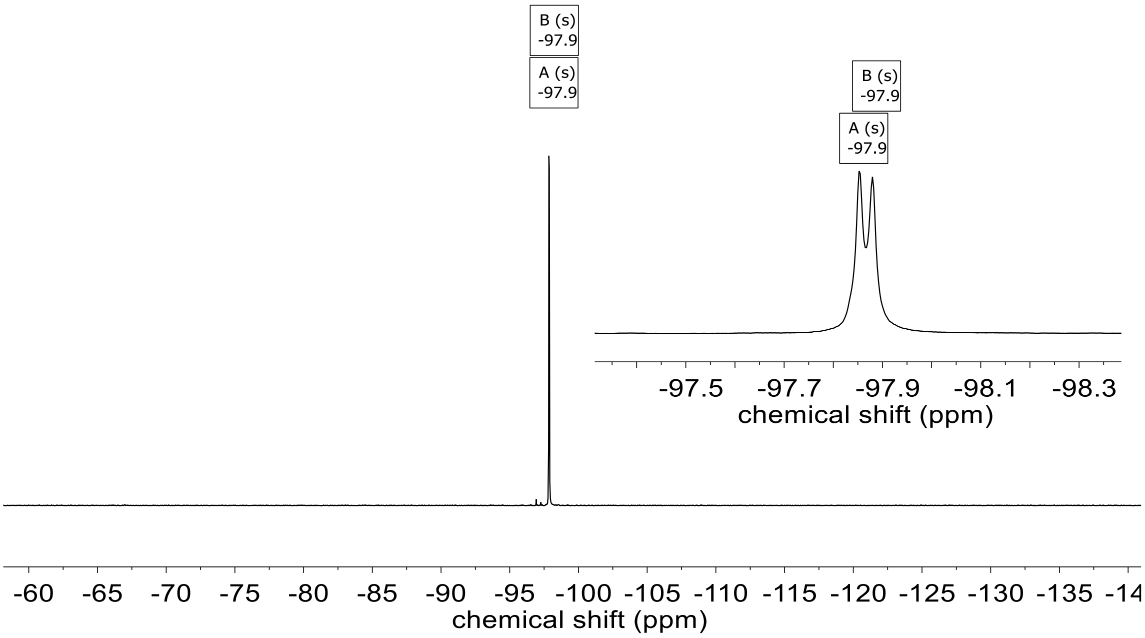

^{1}H-NMR

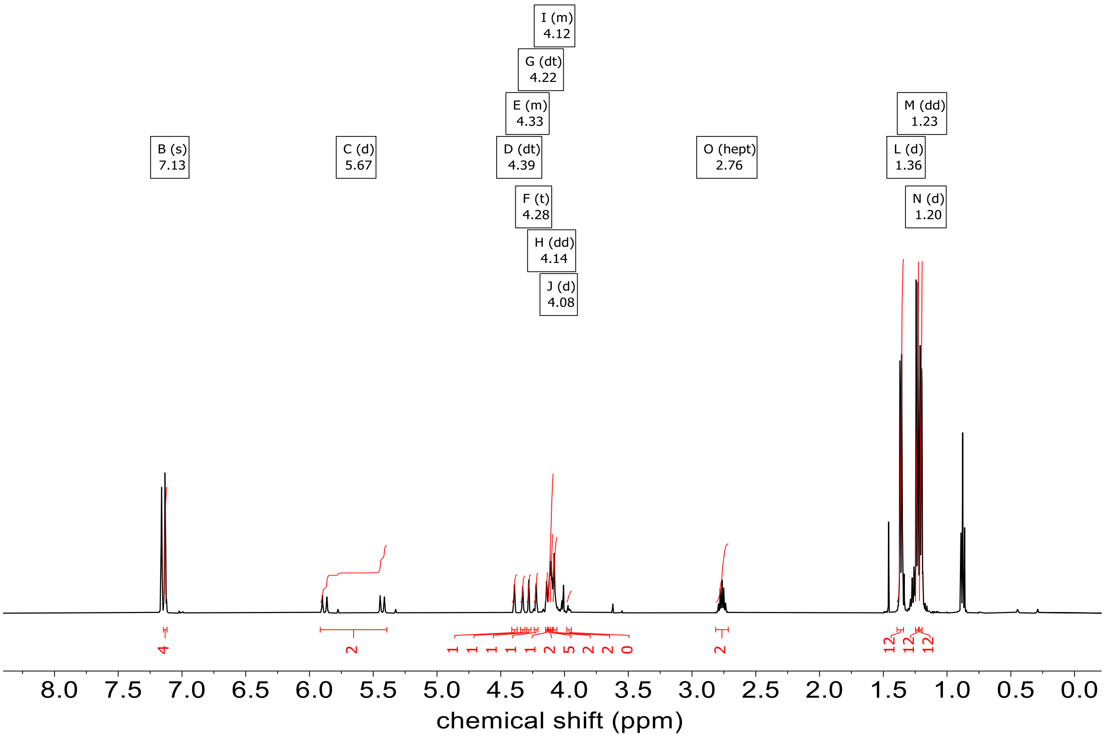

^{13}C-NMR

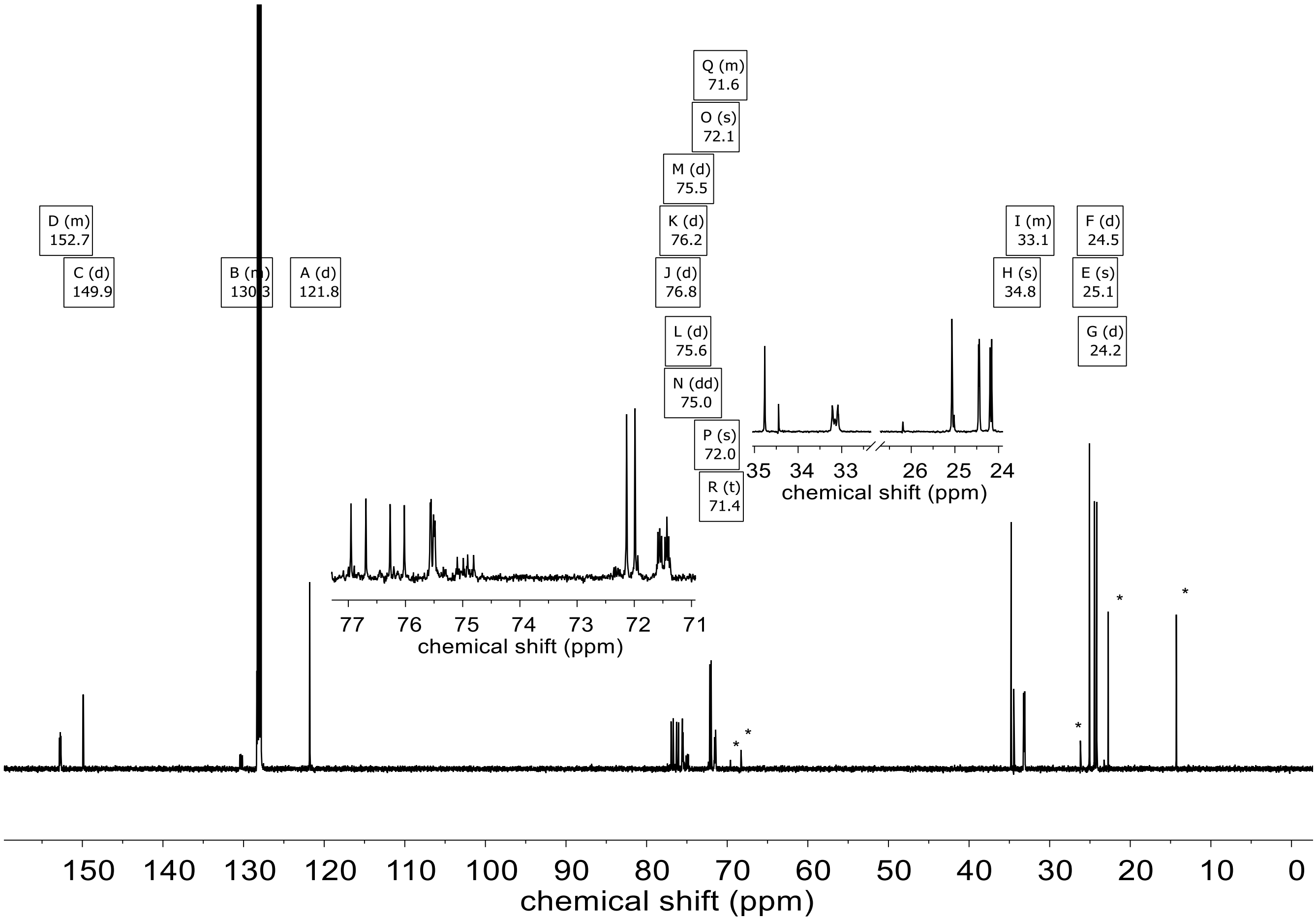

CH-HSQC

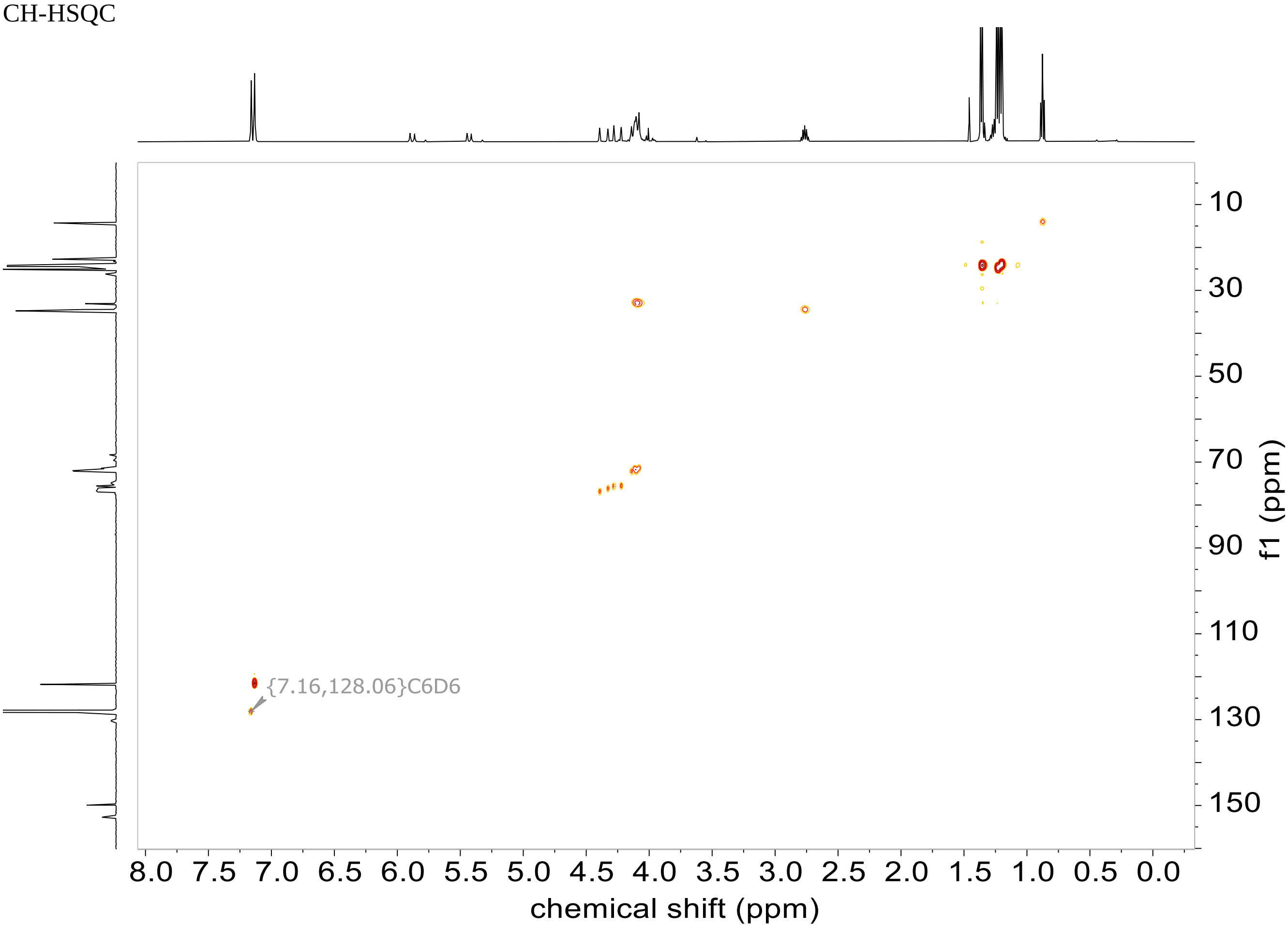

CH-HMBC

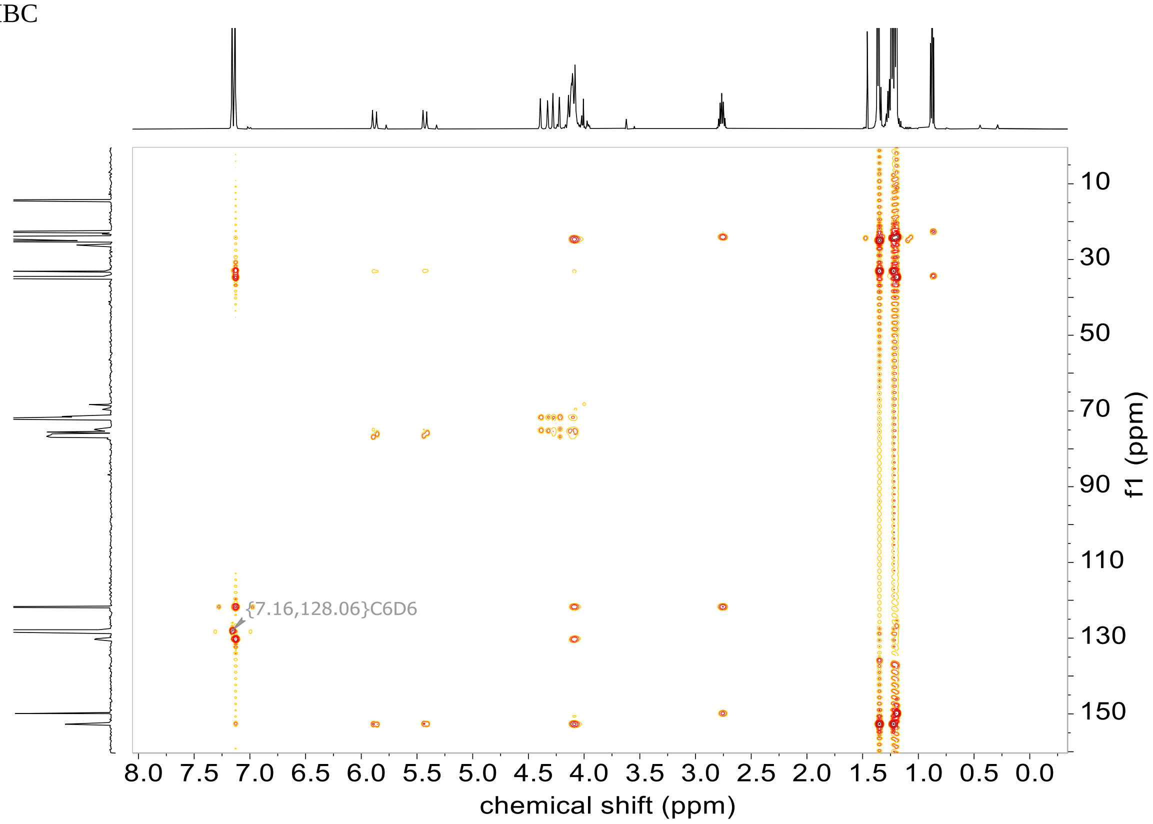
{7.16,128.06}C6D6
8.0 7.5 7.0 6.5 6.0 5.5 5.0 4.5 4.0 3.5 3.0 2.5 2.0 1.5 1.0 0.5 0.0
chemical shift (ppm)
10 30 50 70 90 110 130 150
f1 (ppm)

NMR-spectra of **3**:

$^{31}P\{^{1}H\}$-NMR

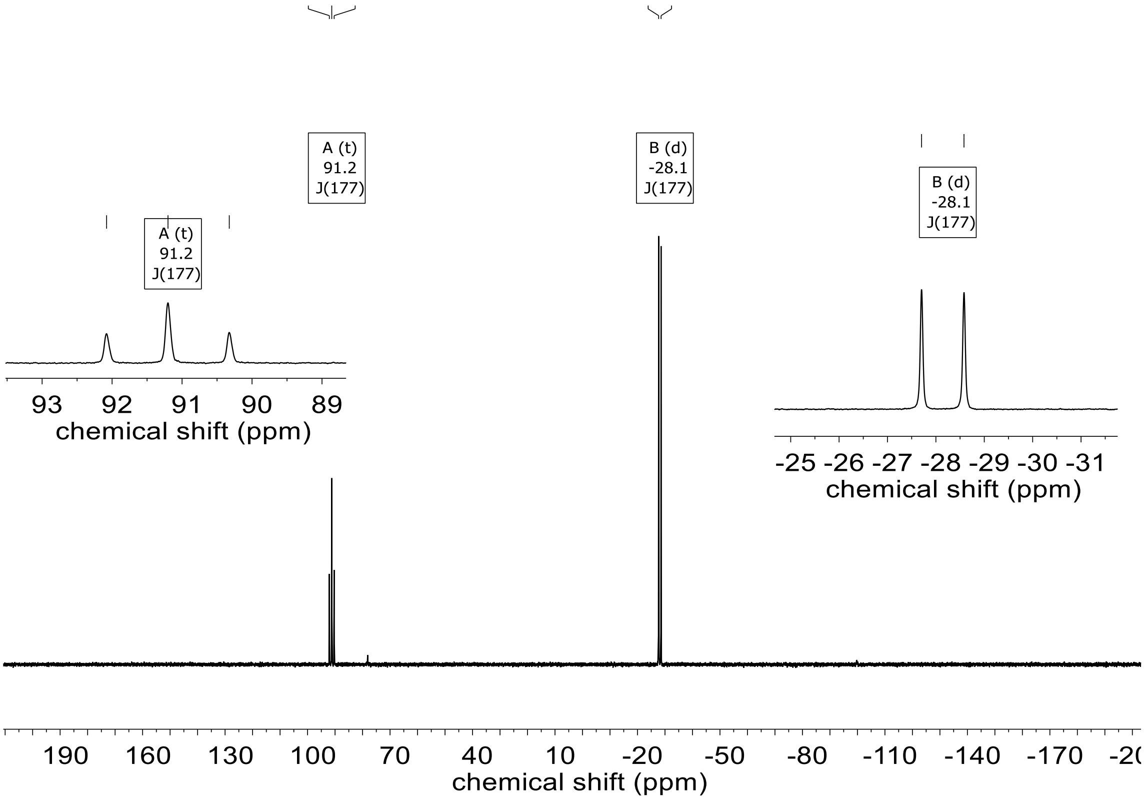

^{1}H-NMR

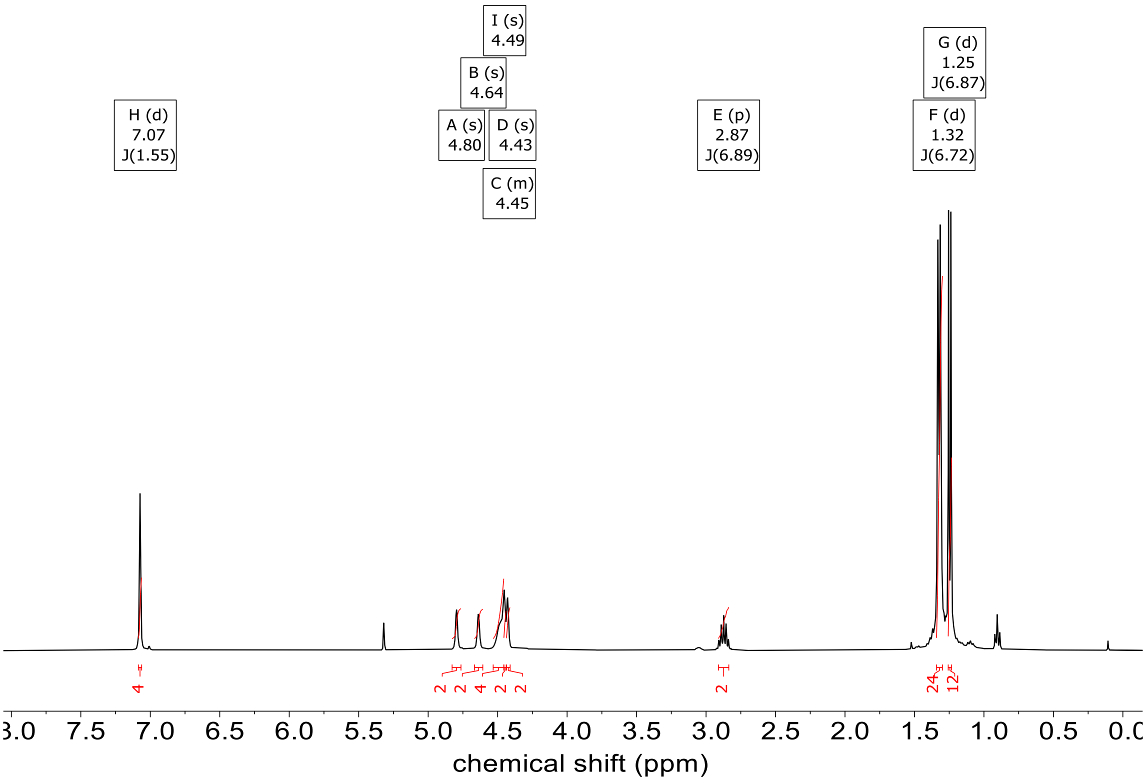

^{13}C-NMR

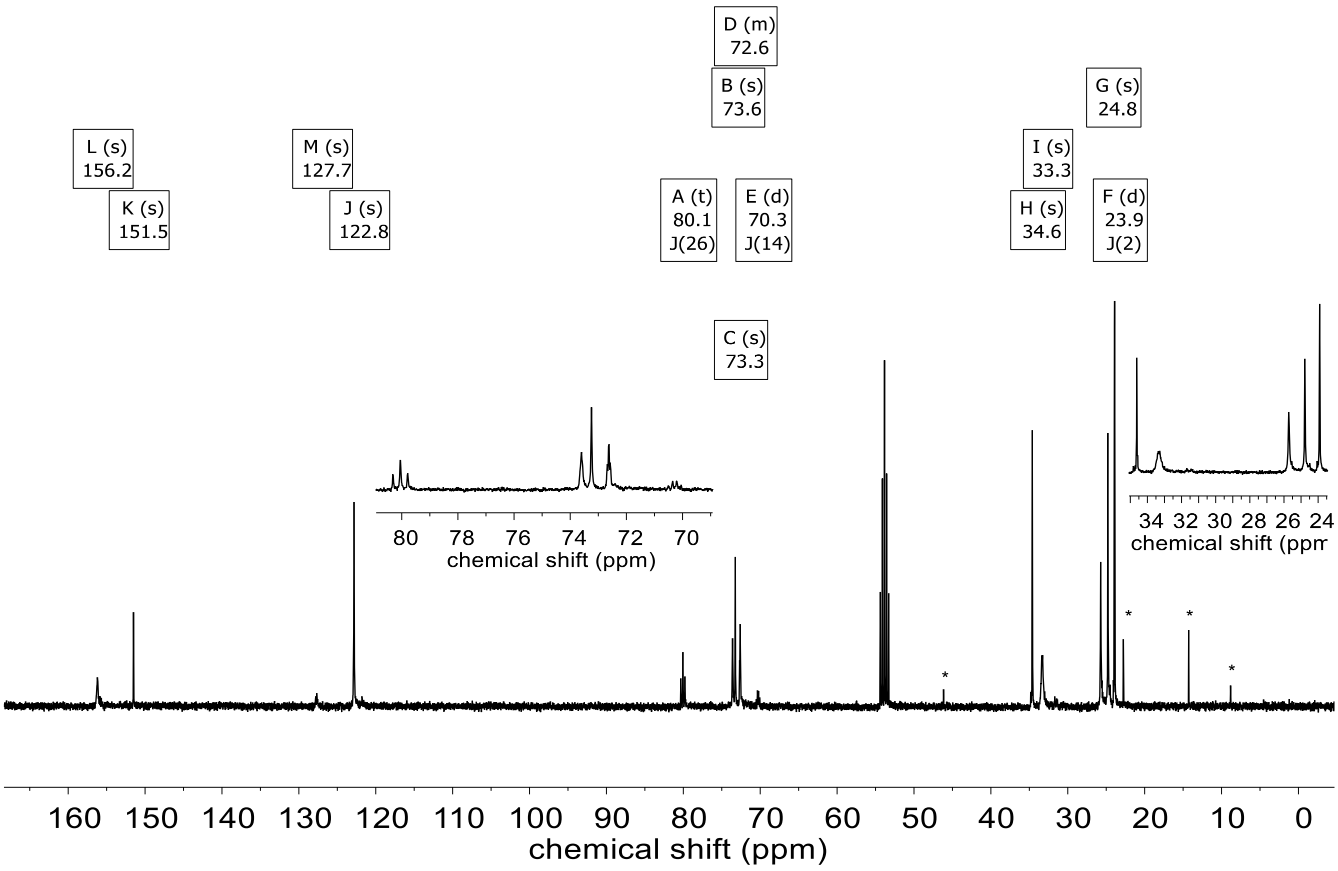

CH-HSQC

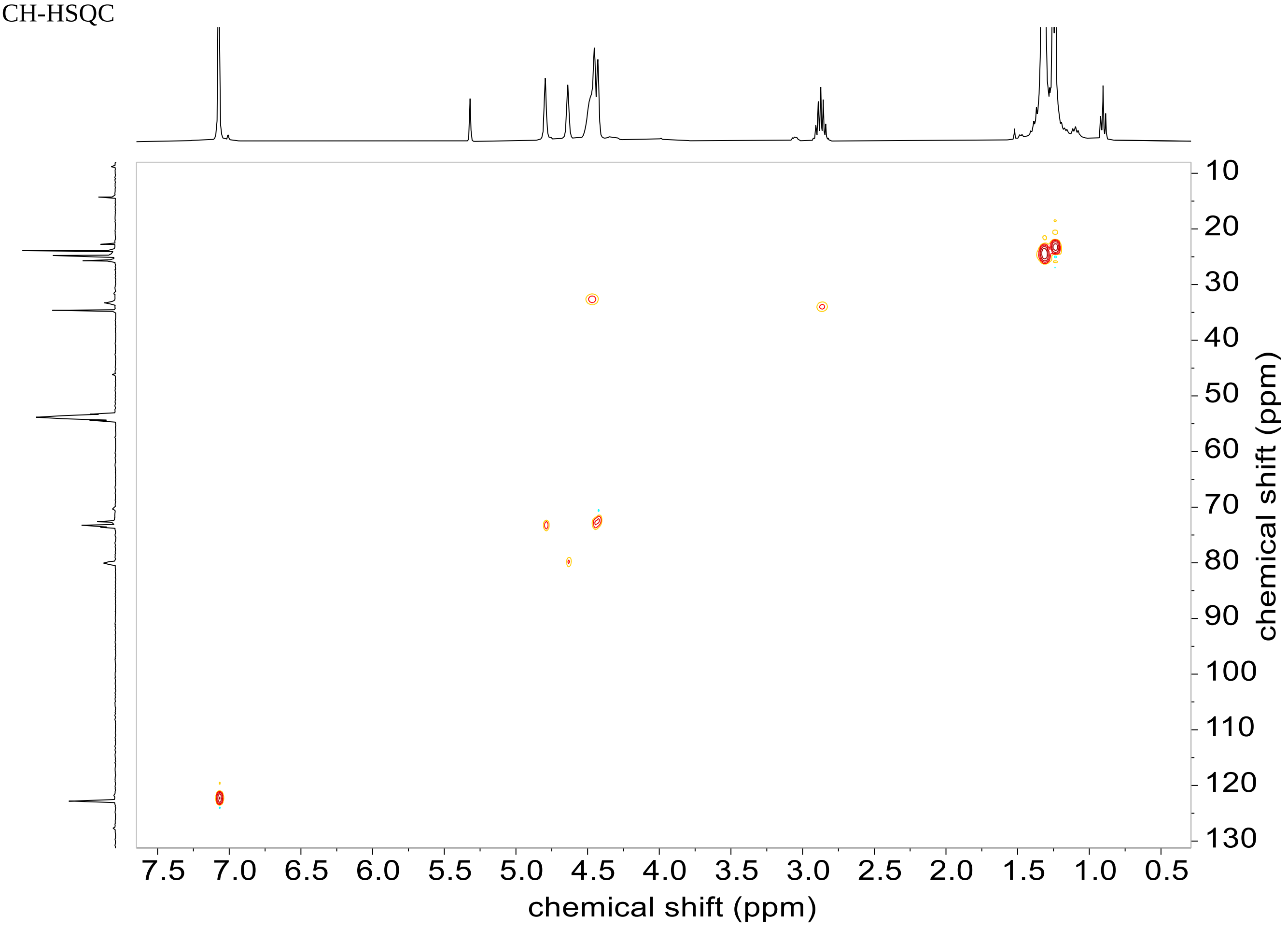

NMR-spectra of **4**[$AlCl_4$]$_2$:

^{1}H-NMR

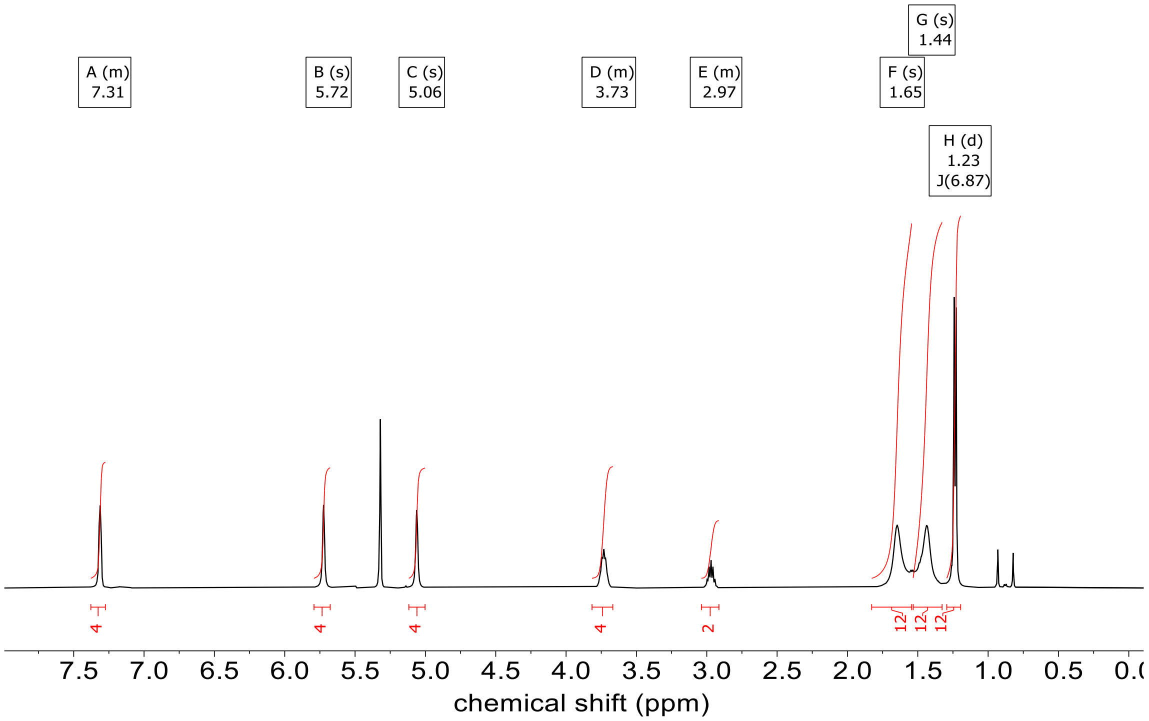

^{13}C-NMR

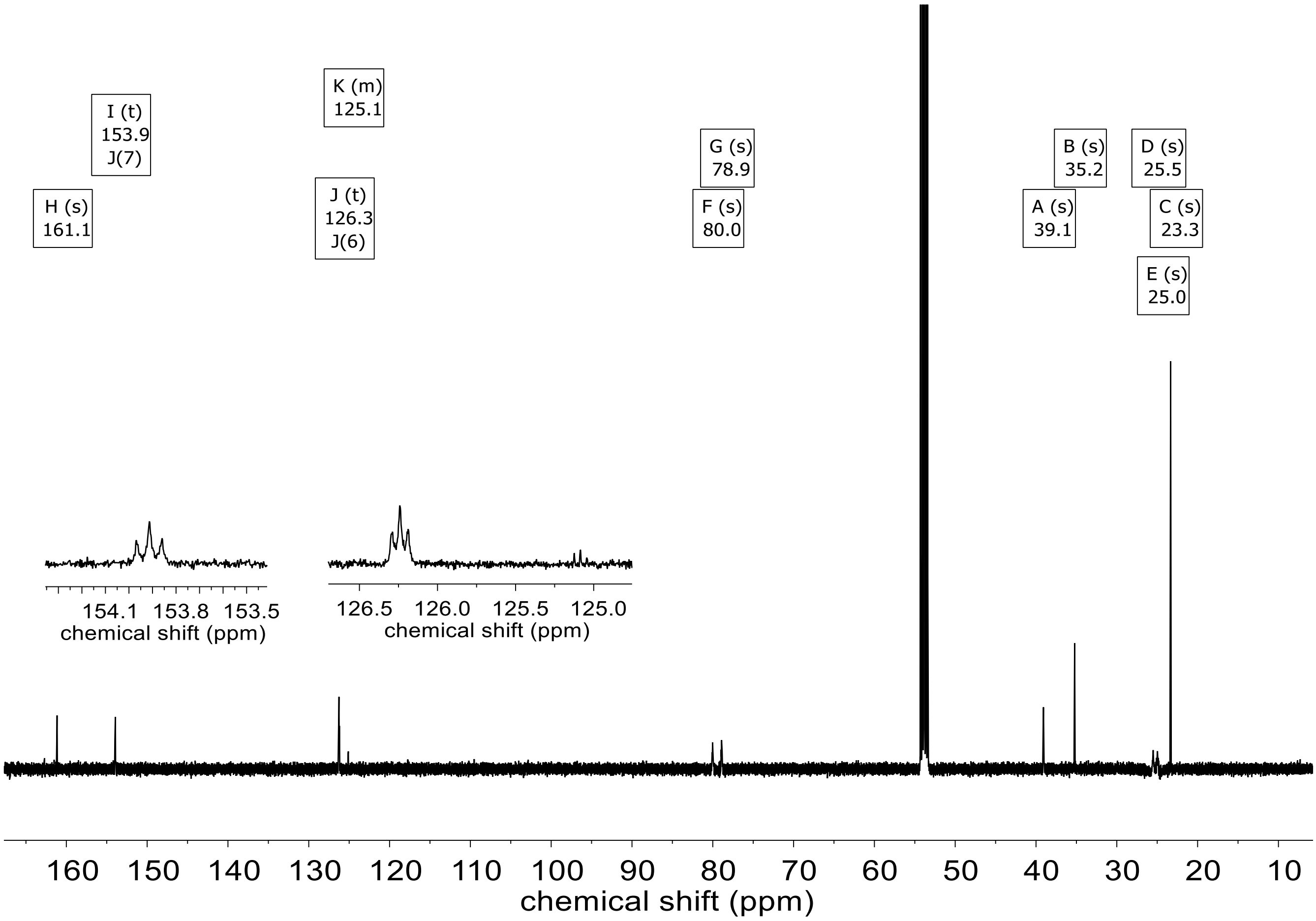

^{27}Al-NMR

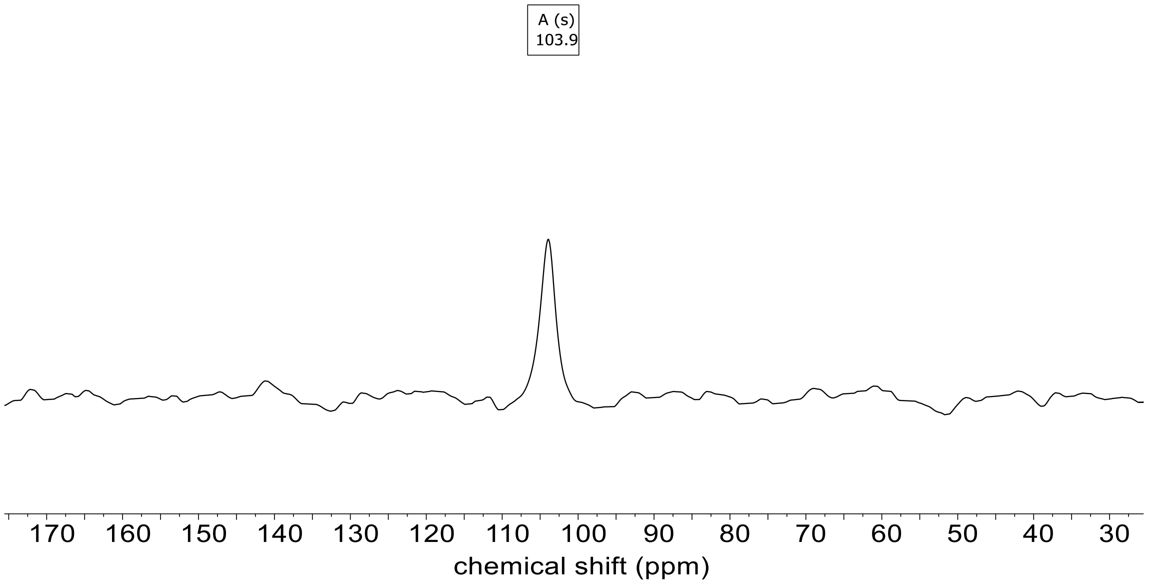

^{31}P-NMR

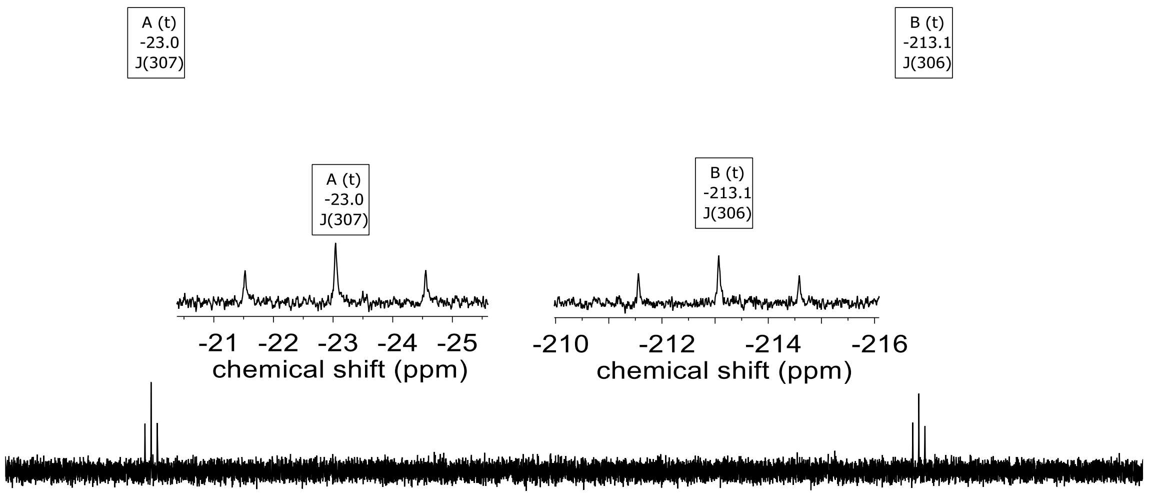

NMR-spectra of **5**[Al_2Cl_7]:

^{31}P-NMR

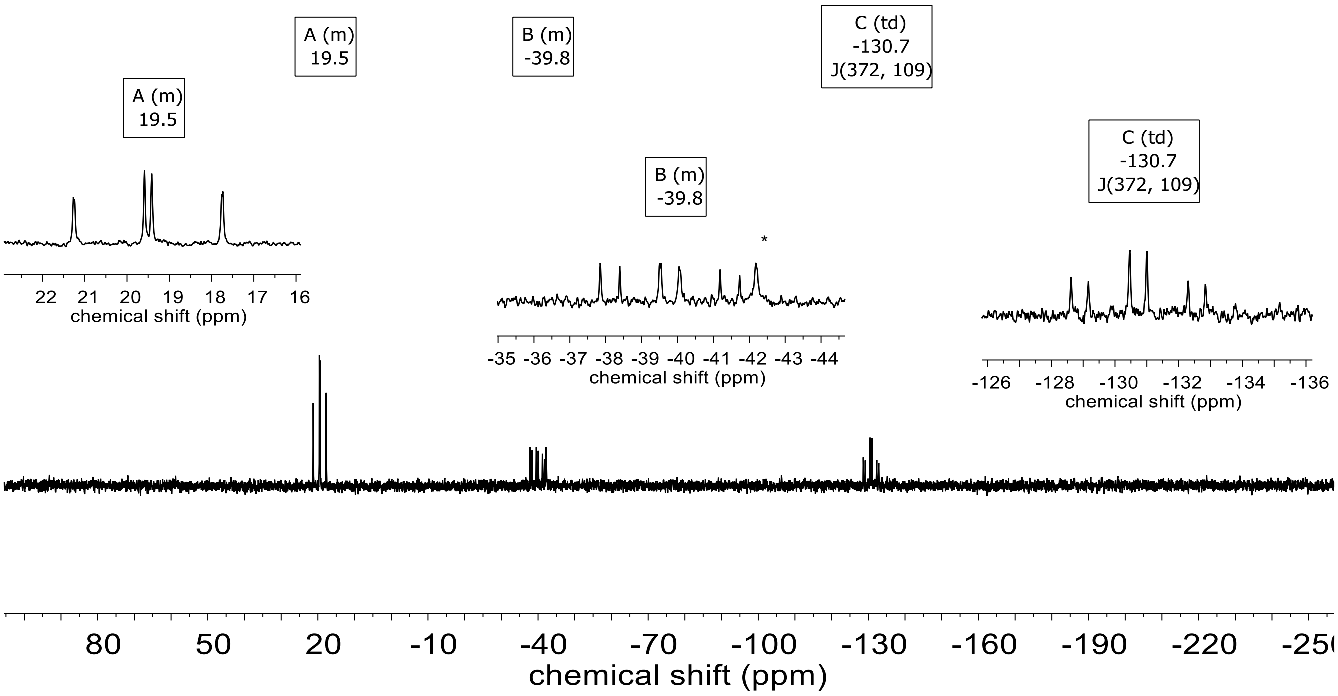

^{1}H-NMR

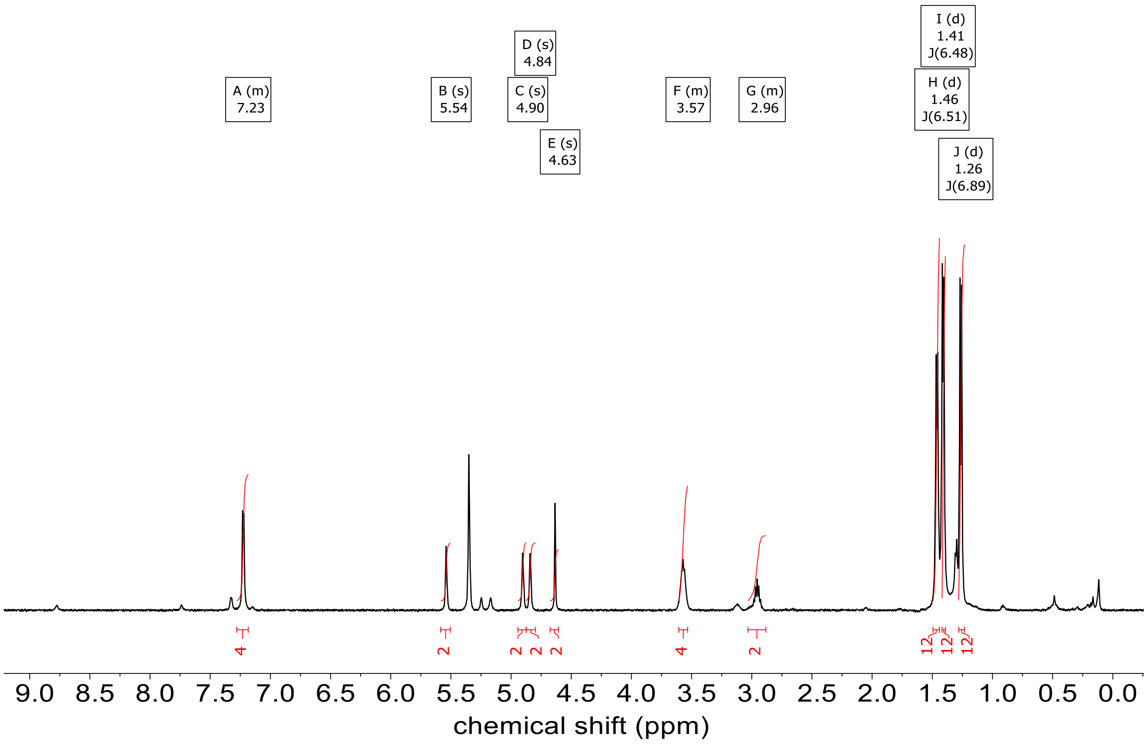

CH-HMBC

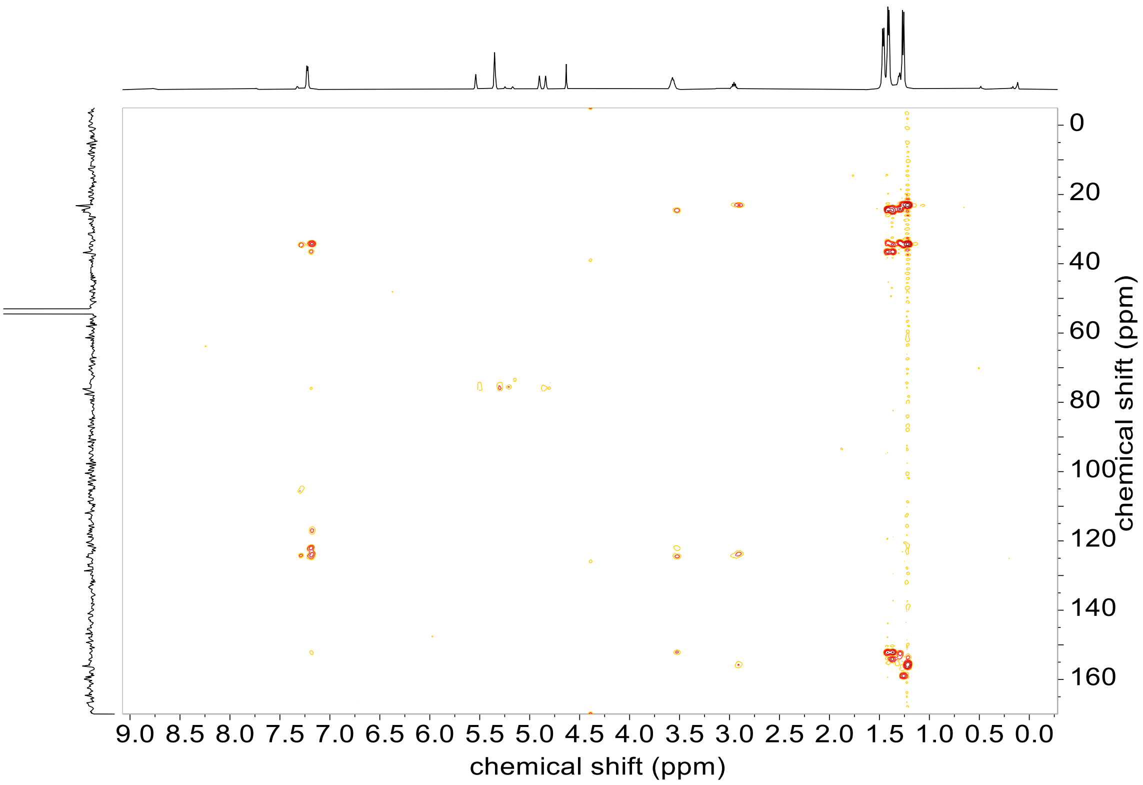

^{27}Al-NMR

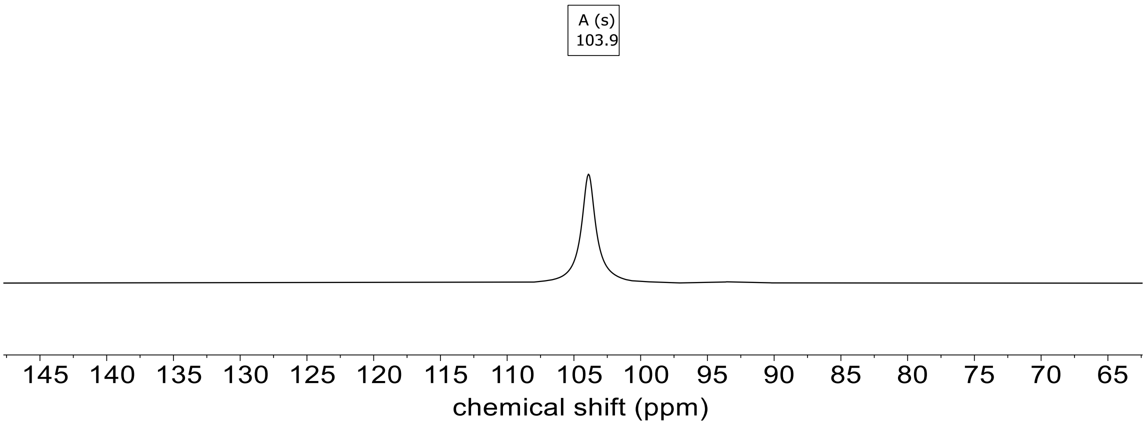

NMR-spectra of **6**[$AlCl_4$]:
^{31}P-NMR

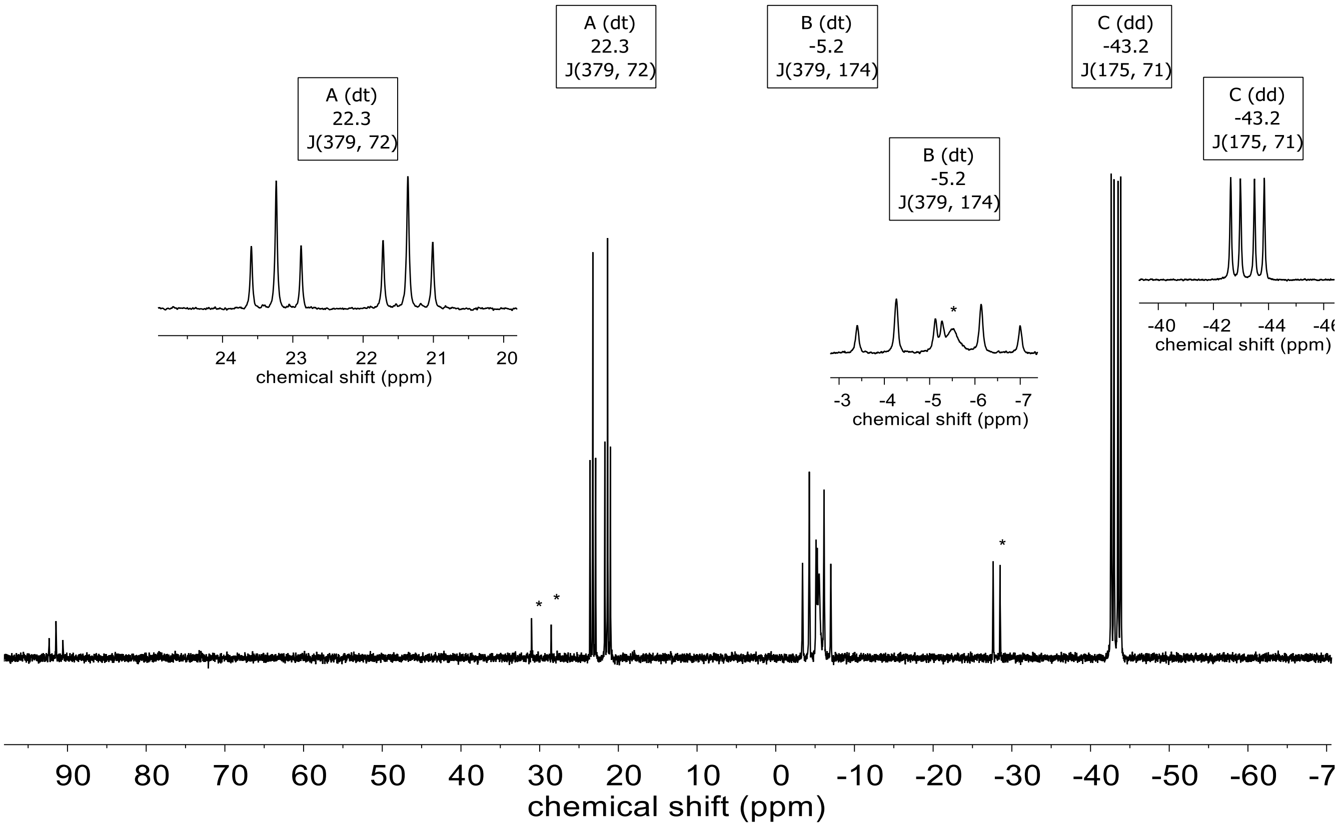

^{1}H-NMR

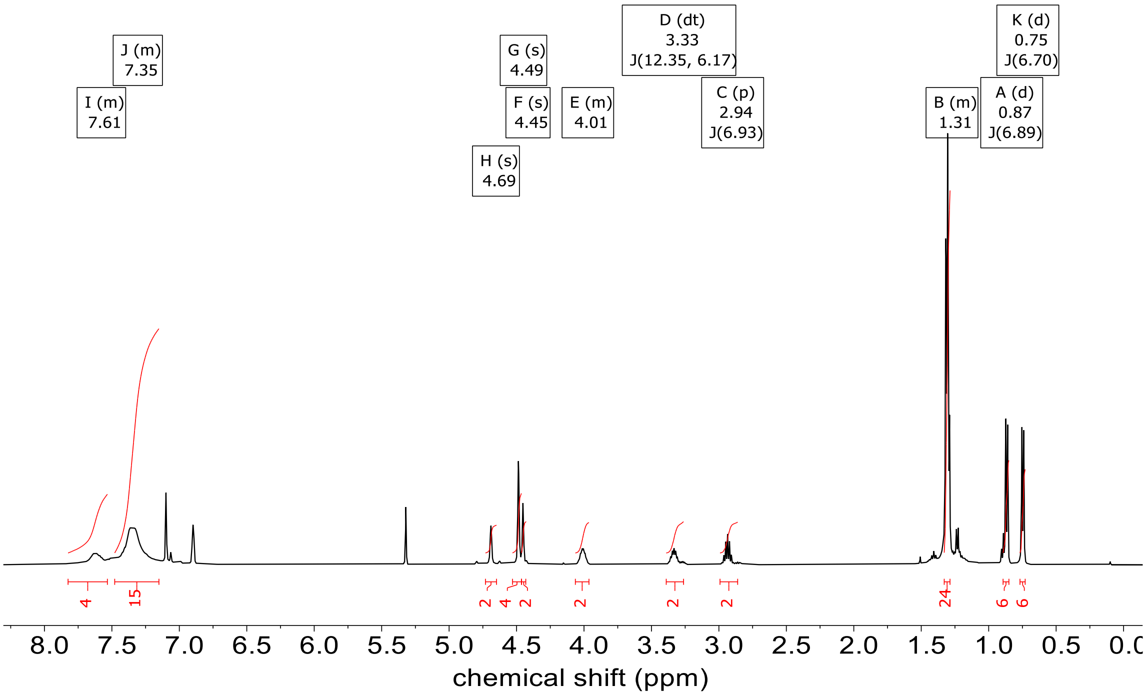

^{13}C-NMR

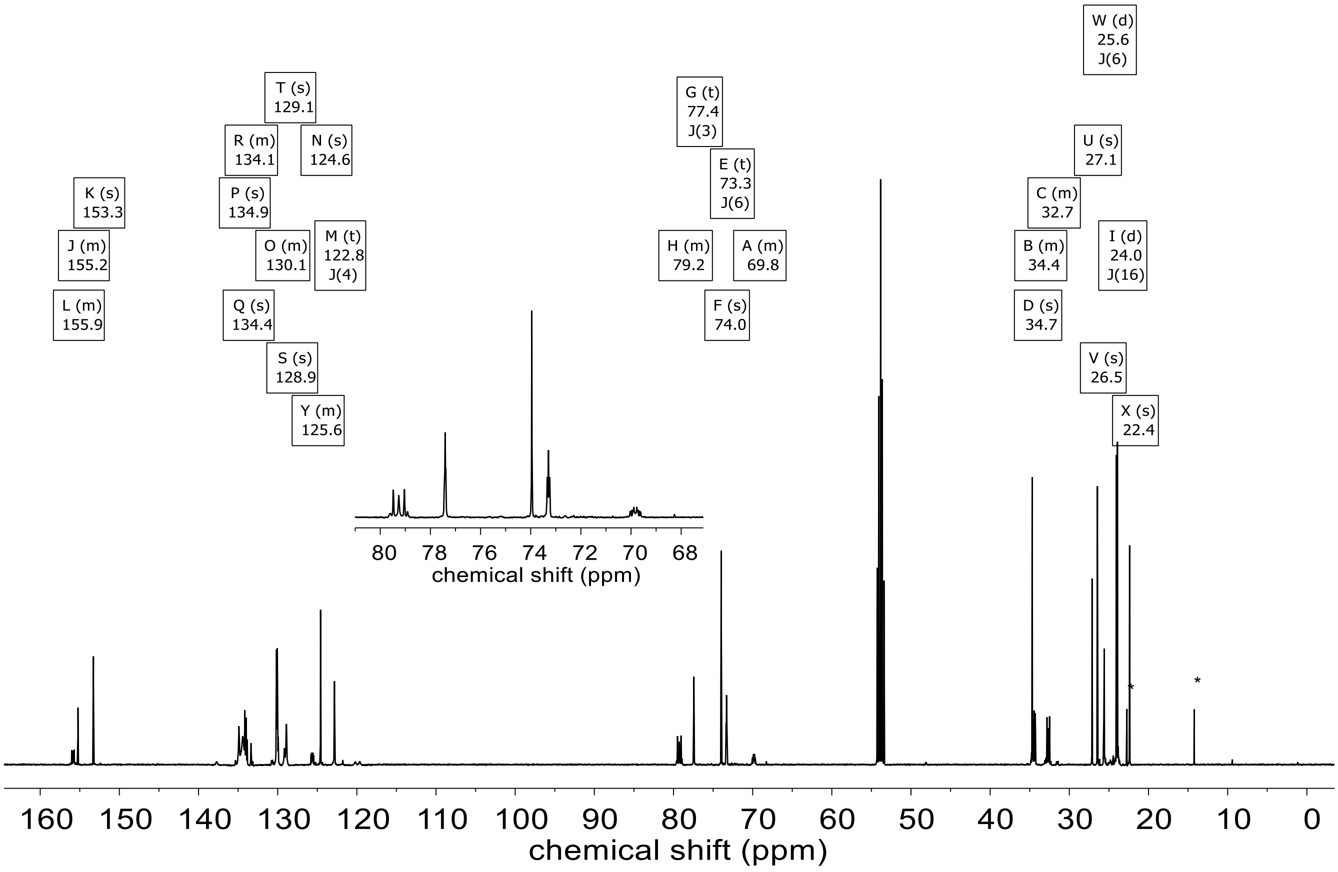

CH-HSQC

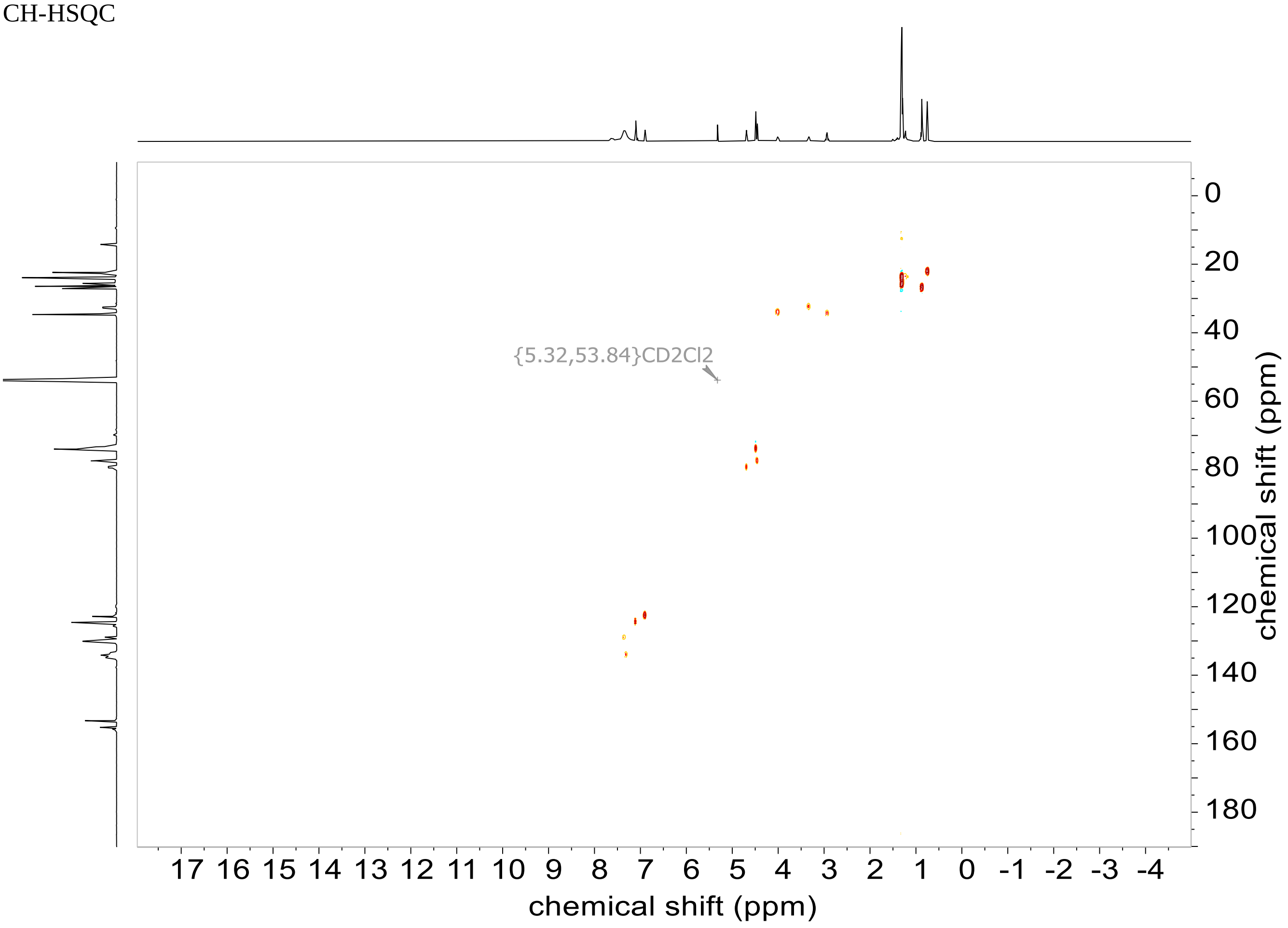

^{27}Al-NMR

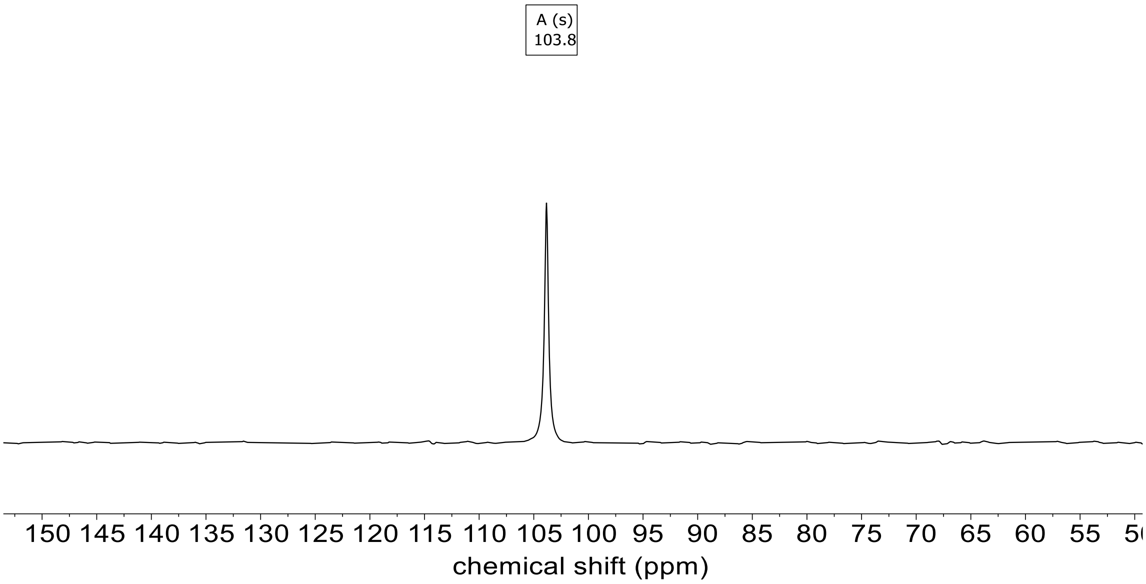

A (s)
103.8
150 145 140 135 130 125 120 115 110 105 100 95 90 85 80 75 70 65 60 55 5
chemical shift (ppm)

NMR-spectra of **8c**[BF_4]:

^{31}P-NMR

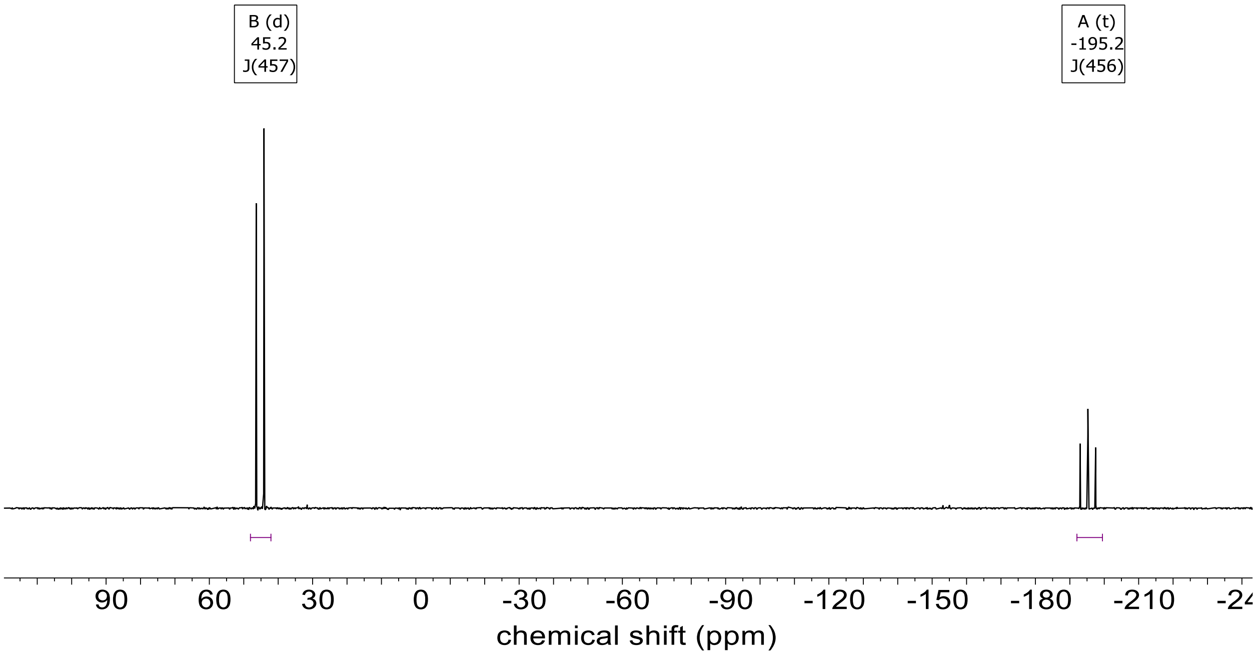

^{1}H-NMR

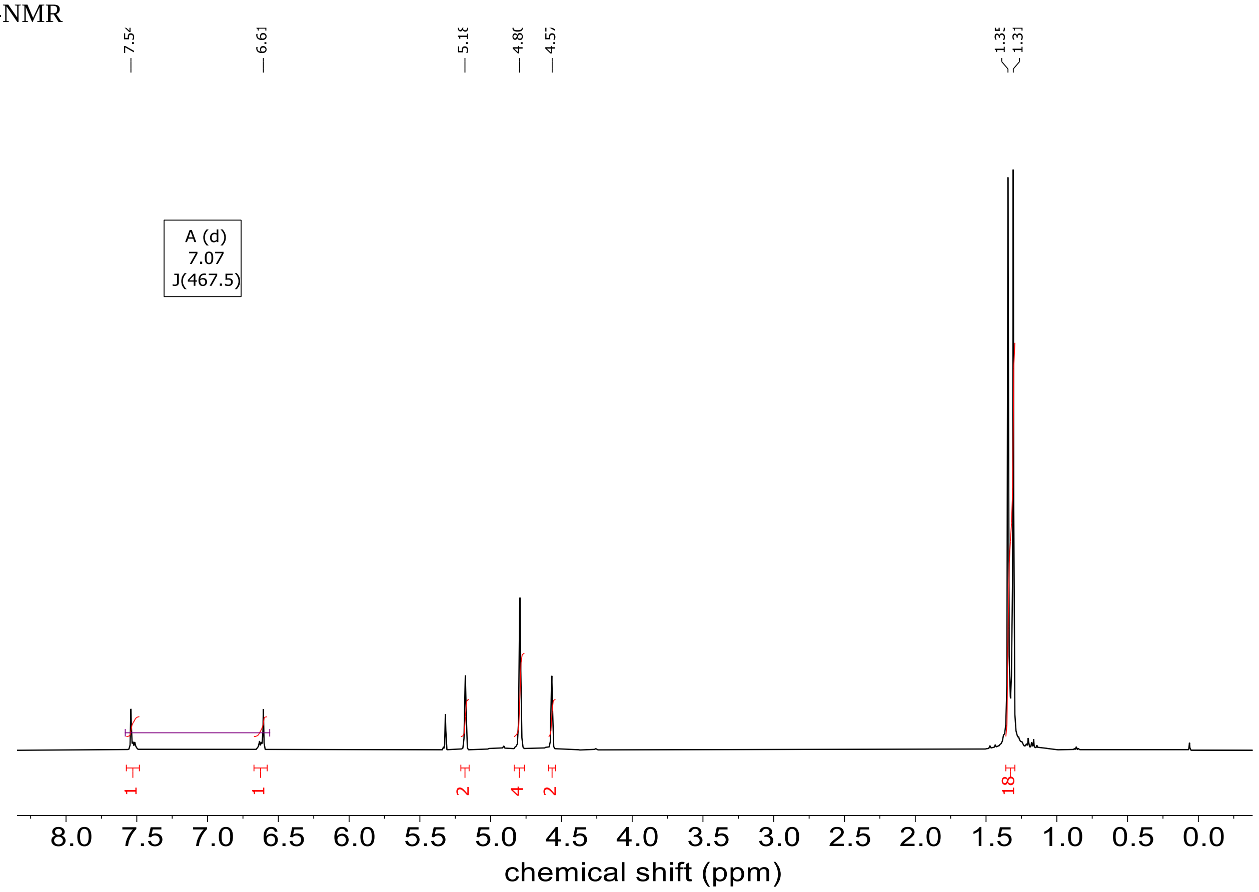

^{13}C-NMR

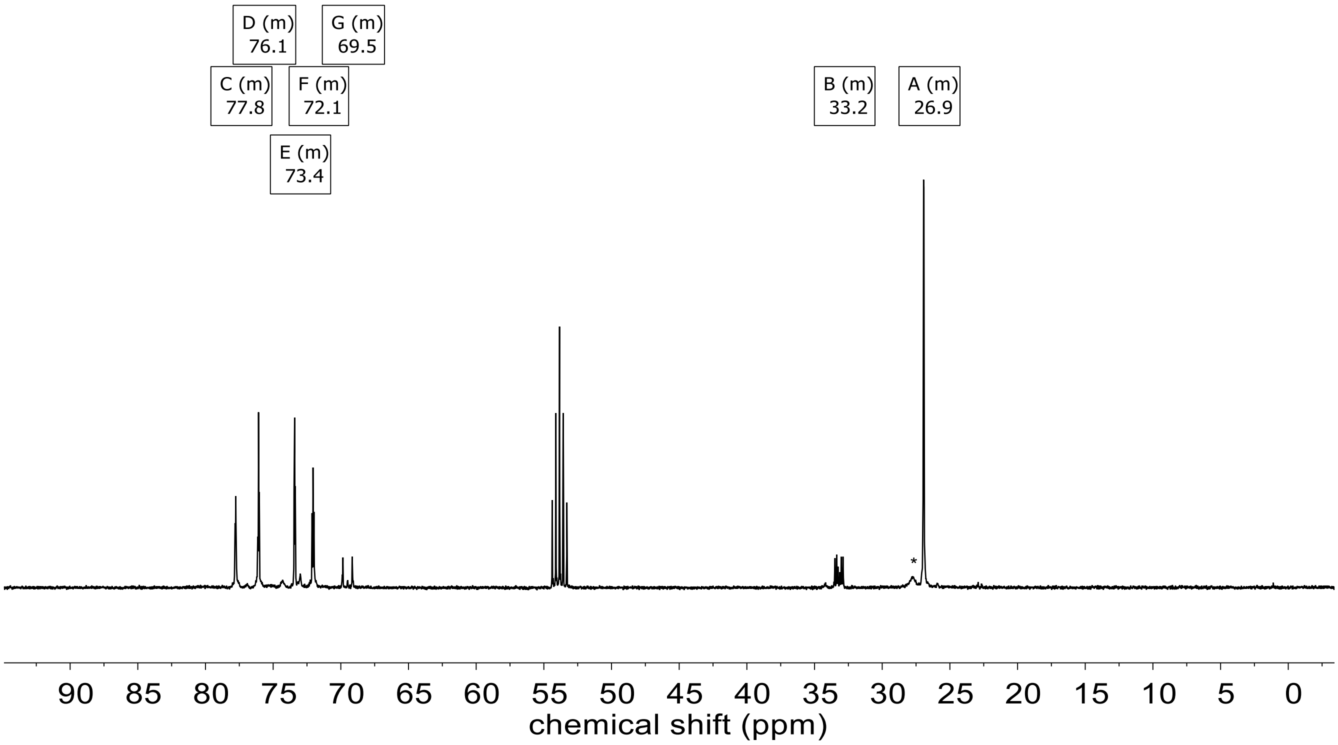

^{11}B-NMR

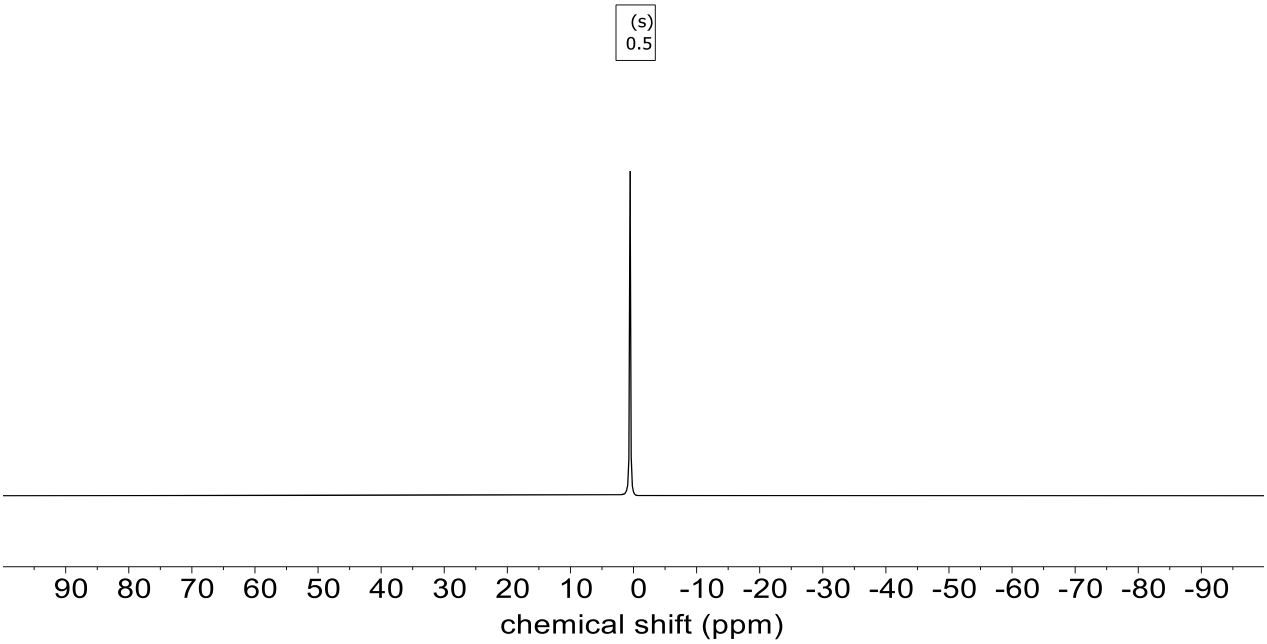

^{19}F-NMR

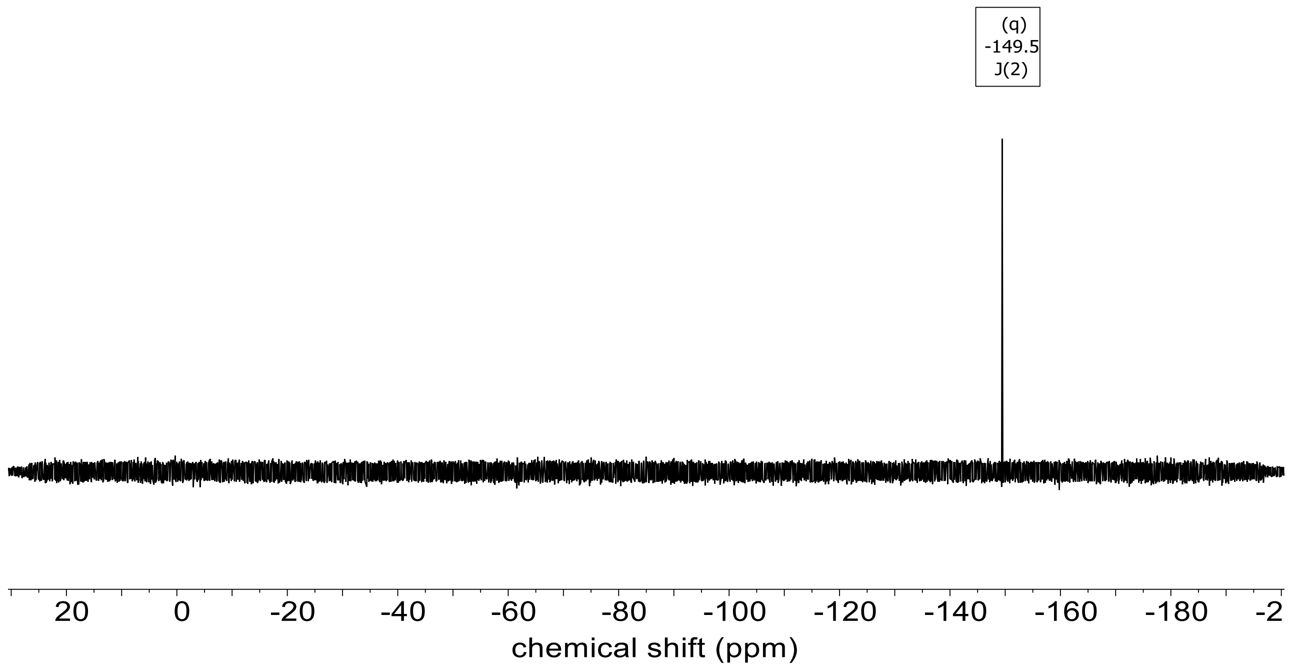

(q)
-149.5
J(2)
20
0
-20
-40
-60
-80
-100
-120
-140
-160
-180
-2
chemical shift (ppm)

References

1. C. Moser, A. Orthaber, M. Nieger, F. Belaj and R. Pietschnig, *Dalton Trans.*, 2006, 3879-3885.
2. D. Kargin, Z. Kelemen, K. Krekić, M. Maurer, C. Bruhn, L. Nyulászi and R. Pietschnig, *Dalton Trans.*, 2016, **45**, 2180 - 2189.
3. S. Borucki, Z. Kelemen, M. Maurer, C. Bruhn, L. Nyulaszi and R. Pietschnig, *Chem. Eur. J.*, 2017, **23**, 10438-10450.
4. N. Kano, K. Shibata, N. Tokitoh and R. Okazaki, *Organometallics*, 1999, **18**, 2999-3007.
5. R. K. Harris, E. D. Becker, S. M. Cabral de Menezes, R. Goodfellow and P. Granger, *Pure & Appl. Chem.*, 2001, **73**, 1795-1818.
6. G. M. Sheldrick, *Acta Crystallogr., Sect. C: Struct. Chem.*, 2015, **71**, 3-8.
7. O. V. Dolomanov, L. J. Bourhis, R. J. Gildea, J. A. K. Howard and H. Puschmann, *J. Appl. Cryst.*, 2009, **42**, 339-341.
8. C. F. Macrae, P. R. Edgington, P. McCabe, E. Pidcock, G. P. Shields, R. Taylor, M. Towler and J. van de Streek, *J. Appl. Crystallogr.*, 2006, **39**, 453-457.
9. M. J. Frisch, G. W. Trucks, H. B. Schlegel, G. E. Scuseria, M. A. Robb, J. R. Cheeseman, G. Scalmani, V. Barone, G. A. Petersson, H. Nakatsuji, X. Li, M. Caricato, A. V. Marenich, J. Bloino, B. G. Janesko, R. Gomperts, B. Mennucci, H. P. Hratchian, J. V. Ortiz, A. F. Izmaylov, J. L. Sonnenberg, D. Williams-Young, F. Ding, F. Lipparini, F. Egidi, J. Goings, B. Peng, A. Petrone, T. Henderson, D. Ranasinghe, V. G. Zakrzewski, J. Gao, N. Rega, G. Zheng, W. Liang, M. Hada, M. Ehara, K. Toyota, R. Fukuda, J. Hasegawa, M. Ishida, T. Nakajima, Y. Honda, O. Kitao, H. Nakai, T. Vreven, K. Throssell, J. A. Montgomery Jr., J. E. Peralta, F. Ogliaro, M. Bearpark, J. J. Heyd, E. Brothers, K. N. Kudin, V. N. Staroverov, T. Keith, R. Kobayashi, J. Normand, K. Raghavachari, A. Rendell, J. C. Burant, S. S. Iyengar, J. Tomasi, M. Cossi, J. M. Millam, M. Klene, C. Adamo, R. Cammi, J. W. Ochterski, R. L. Martin, K. Morokuma, O. Farkas, J. B. Foresman and D. J. Fox, *Gaussian 16, Revision C.01*, Gaussian, Inc., Wallingford CT, 2016.
10. S. Weller, S. H. Schlindwein, C. M. Feil, Z. Kelemen, D. Buzsáki, L. Nyulászi, S. Isenberg, R. Pietschnig, M. Nieger and D. Gudat, *Organometallics*, 2019, **38**, 4717-4725.
11. S. Dey, F. Roesler, C. Bruhn, Z. Kelemen and R. Pietschnig, *Inorg. Chem. Frontiers*, 2023, **10**, 3828-3843.
12. T. Lu and F. Chen, *J. Comput. Chem.*, 2012, **33**, 580-592.
13. A. T. B. Gilbert, 2018.
14. E. D. Glendening, J. K. Badenhoop, A. E. Reed, J. E. Carpenter, J. A. Bohmann, C. M. Morales, P. Karafiloglou, C. R. Landis and F. Weinhold, *NBO 7.0,* Theoretical Chemistry Institute University of Wisconsin, Madison WI, 2018.
15. R. F. W. Bader and H. Essén, *J. Chem. Phys.*, 1984, **80**, 1943-1960.
16. E. Espinosa, I. Alkorta, J. Elguero and E. Molins, *J. Chem. Phys.*, 2002, **117**, 5529-5542.
17. J. J. Weigand, N. Burford, A. Decken and A. Schulz, *Eur. J. Inorg. Chem.*, 2007, **2007**, 4868-4872.
18. R. W. Alder, C. Ganter, C. J. Harris and A. G. Orpen, *J. Chem. Soc., Chem. Commun.*, 1992, 1172-1174.
19. S. Lochschmidt and A. Schmidpeter, *Z. Naturforsch. B*, 1985, **40**, 765-773.
20. J. D. Burton, R. M. K. Deng, K. B. Dillon, P. K. Monks and R. J. Olivey, *Heteroat. Chem*, 2005, **16**, 447-452.

XYZ coordinates

The following lists contain the XYZ coordinates of the investigated systems and their total energies, which were obtained after the geometry optimization at ωB97X-D/def2-TZVP.

4

$AlCl_3$ SCF done: -1623.28276

Al	-0.00015	0.00032	-0.00039
Cl	1.94769	0.70518	0.00010
Cl	-0.36237	-2.03766	0.00010
Cl	-1.58521	1.33223	0.00010

5

$AlCl_4$ SCF done: -2083.6785117

Al	-0.00020	-0.00011	0.00003
Cl	-0.81562	0.77414	-1.84390
Cl	0.15438	1.59739	1.44535
Cl	-1.29954	-1.54427	0.76937
Cl	1.96094	-0.82718	-0.37085

58

8a$^+[AlCl_4]^-$ SCF done: -4112.91917

C	-4.60825	-0.76407	-1.58943
C	-3.41437	-0.07245	-1.41014
C	-3.28778	1.23558	-1.87419
C	-4.35122	1.83685	-2.52532
C	-5.53772	1.14319	-2.71219
C	-5.66705	-0.15510	-2.24344
P	-2.02665	-0.78317	-0.50899
C	-2.25100	-0.29461	1.22162
C	-1.37531	0.62138	1.78848
C	-1.52751	0.99110	3.11597
C	-2.55334	0.45108	3.87264
C	-3.44212	-0.45312	3.30300
C	-3.29511	-0.82383	1.97921
C	-2.11788	-2.62948	-0.52968
C	-0.71461	-3.22428	-0.73639
P	0.57129	-1.94567	-0.46575
C	2.10475	-2.47557	-1.24278
C	2.43647	-2.03579	-2.52017
C	3.61139	-2.46819	-3.11171
C	4.45025	-3.34011	-2.43554
C	4.12176	-3.77875	-1.16103
C	2.95388	-3.34378	-0.55975
P	-0.19261	-0.19280	-1.37777
C	0.88015	-1.84489	1.30880
C	1.78940	-0.88327	1.74494
C	1.98711	-0.68840	3.09984
C	1.27363	-1.43882	4.02255
C	0.36262	-2.39093	3.59257
C	0.16606	-2.59715	2.23714
Cl	2.89370	1.44767	-1.07173
Al	1.88249	3.13797	-0.14910

Cl	-0.09250	3.39382	-1.00144
Cl	3.04164	4.90339	-0.41059
Cl	1.63773	2.67829	1.95627
H	-2.55516	-2.96542	0.40962
H	-2.77948	-2.92978	-1.34058
H	-0.54344	-4.10249	-0.11408
H	-0.57563	-3.52362	-1.77459
H	2.31767	-0.25985	1.03157
H	2.67771	0.07678	3.42684
H	1.41953	-1.27206	5.08216
H	-0.20336	-2.96890	4.31137
H	-0.55907	-3.33515	1.92001
H	1.79117	-1.34178	-3.04348
H	3.87728	-2.10977	-4.09717
H	5.37171	-3.66977	-2.89817
H	4.78326	-4.44916	-0.62822
H	2.71387	-3.66290	0.44698
H	-0.56643	1.04693	1.20737
H	-0.83228	1.70260	3.54169
H	-2.66949	0.73796	4.91015
H	-4.25209	-0.86481	3.89138
H	-4.00270	-1.51889	1.54056
H	-2.36724	1.79058	-1.72457
H	-4.24841	2.85180	-2.88567
H	-6.36589	1.61717	-3.22320
H	-6.59365	-0.69590	-2.38559
H	-4.72566	-1.77591	-1.22110

99
isomer-1 of **3** SCF done: -4305.004198

C	3.92963	-0.20182	0.95862
C	3.16260	-0.62937	-0.14163
C	3.64602	-1.69434	-0.93973
C	4.86523	-2.27871	-0.61640
C	5.63789	-1.84918	0.44748
C	5.14990	-0.80803	1.21809
P	1.56565	0.09298	-0.71000
C	1.66355	1.90017	-0.82561
C	1.61928	2.97510	0.11684
C	1.64050	4.19727	-0.59683
C	1.68267	3.90212	-1.98412
C	1.69542	2.49582	-2.12858
Fe	0.00010	3.07262	-1.11719
C	-1.69862	2.49924	-2.12474
C	-1.66364	1.90133	-0.82293
C	-1.61562	2.97453	0.12130
C	-1.63768	4.19796	-0.59012
C	-1.68403	3.90529	-1.97781
P	-1.56595	0.09393	-0.71016
P	-0.00030	-0.54776	0.65279
Cl	-0.00083	0.60067	2.42572
C	2.86883	-2.28451	-2.10869
C	1.95232	-3.40626	-1.61357
C	6.96630	-2.50190	0.75939

C	6.95350	-3.16678	2.13702
C	3.49456	0.90456	1.89356
C	4.35735	2.15334	1.70779
C	-3.16264	-0.62923	-0.14191
C	-3.64516	-1.69480	-0.93975
C	-4.86419	-2.27969	-0.61666
C	-5.63751	-1.85013	0.44672
C	-5.15038	-0.80841	1.21711
C	-3.93032	-0.20168	0.95790
C	-2.86719	-2.28509	-2.10812
C	-1.95071	-3.40653	-1.61225
C	-3.49624	0.90552	1.89233
C	-3.48091	0.45792	3.35445
C	-6.96573	-2.50339	0.75834
C	-6.95322	-3.16764	2.13627
C	-4.35837	2.15435	1.70382
C	-8.12140	-1.50867	0.63677
C	-3.74644	-2.77552	-3.25789
C	3.74882	-2.77447	-3.25809
C	8.12152	-1.50654	0.63878
C	3.47713	0.45514	3.35512
H	-2.47215	1.15818	1.64193
H	-2.22691	-1.50107	-2.51034
H	1.70460	1.94734	-3.05685
H	-5.23122	-3.10081	-1.21943
H	1.59383	5.18162	-0.15941
H	-4.48591	0.25233	3.72876
H	-3.04925	1.24269	3.97903
H	-2.87855	-0.44220	3.48166
H	-1.58880	5.18149	-0.15110
H	-1.71093	1.95246	-3.05398
H	-7.12155	-3.28762	0.01186
H	1.54138	2.88088	1.18561
H	-1.68301	4.62690	-2.77859
H	1.68002	4.62230	-2.78618
H	-5.73548	-0.45749	2.05932
H	-5.39722	1.95481	1.97701
H	-4.33937	2.49301	0.66725
H	-3.99633	2.96932	2.33491
H	-1.53475	2.87819	1.18968
H	-6.14091	-3.89143	2.21651
H	-7.89613	-3.68647	2.32086
H	-6.81963	-2.42598	2.92708
H	-1.25631	-3.04484	-0.85202
H	-1.36572	-3.81887	-2.43713
H	-2.53831	-4.21544	-1.17064
H	-4.29694	-3.68380	-3.00382
H	-3.11872	-3.01371	-4.11863
H	-4.46750	-2.01546	-3.56318
H	-8.02904	-0.70932	1.37559
H	-9.07752	-2.00874	0.80425
H	-8.14235	-1.04911	-0.35247
H	5.23295	-3.09937	-1.21937
H	5.73447	-0.45716	2.06069

H	2.22860	-1.50054	-2.51107
H	7.12285	-3.28572	0.01263
H	2.47087	1.15788	1.64200
H	7.89659	-3.68522	2.32180
H	6.81919	-2.42553	2.92809
H	6.14152	-3.89100	2.21658
H	9.07782	-2.00622	0.80642
H	8.14265	-1.04651	-0.35024
H	8.02846	-0.70759	1.37794
H	3.12162	-3.01254	-4.11924
H	4.46990	-2.01419	-3.56277
H	4.29934	-3.68272	-3.00398
H	1.36803	-3.81869	-2.43890
H	2.53984	-4.21503	-1.17162
H	1.25729	-3.04482	-0.85380
H	3.04509	1.23929	3.98019
H	2.87416	-0.44484	3.48040
H	4.48158	0.24850	3.73031
H	3.99475	2.96761	2.33946
H	5.39578	1.95320	1.98212
H	4.33983	2.49333	0.67162

53

8a$^+$ SCF done: -2029.1478817

C	-4.11515	-0.89198	-0.44790
C	-2.92389	-1.47485	-0.02745
C	-2.87681	-2.83950	0.25857
C	-4.01972	-3.60803	0.14188
C	-5.20962	-3.02283	-0.27093
C	-5.25573	-1.67074	-0.56847
P	-1.40965	-0.51377	0.11777
C	-1.83996	1.23511	-0.05029
C	-1.38180	1.96601	-1.13916
C	-1.67671	3.31668	-1.23954
C	-2.43067	3.93759	-0.25749
C	-2.90078	3.20853	0.82731
C	-2.60780	1.86111	0.93254
C	-0.73516	-0.69524	1.83166
C	0.78001	-0.92925	1.81370
P	1.48230	-0.49209	0.18384
C	3.08413	-1.28367	-0.00134
C	3.15374	-2.60620	-0.43617
C	4.38266	-3.23677	-0.52351
C	5.53979	-2.55444	-0.17698
C	5.47248	-1.23904	0.25713
C	4.24776	-0.59925	0.34501
P	0.06918	-1.23627	-1.22162
C	1.73004	1.29428	0.13002
C	2.21498	1.84850	-1.05459
C	2.32872	3.21989	-1.18236
C	1.94435	4.04963	-0.13677
C	1.45721	3.50508	1.03922
C	1.35347	2.12907	1.17629

H	-0.98926	0.21012	2.38099
H	-1.25389	-1.52659	2.30662
H	1.29190	-0.39820	2.61617
H	1.01058	-1.98858	1.92599
H	2.49671	1.20669	-1.88126
H	2.71292	3.64417	-2.10056
H	2.02705	5.12359	-0.24128
H	1.15753	4.14927	1.85504
H	0.96938	1.72467	2.10330
H	2.25169	-3.13952	-0.71097
H	4.43725	-4.26107	-0.86728
H	6.49943	-3.04953	-0.24946
H	6.37591	-0.70728	0.52440
H	4.20183	0.43088	0.67445
H	-0.78143	1.48082	-1.89835
H	-1.31288	3.88288	-2.08659
H	-2.65860	4.99269	-0.33631
H	-3.49800	3.69096	1.58978
H	-2.99033	1.29941	1.77723
H	-1.94439	-3.30450	0.55709
H	-3.98277	-4.66572	0.36618
H	-6.10253	-3.62697	-0.36523
H	-6.18110	-1.21732	-0.89784
H	-4.15777	0.16290	-0.68417

99
isomer-2 of **3** SCF done: -4305.0049984

C	0.000000	0.000000	0.000000
C	0.000000	0.000000	1.414331
C	1.235097	0.000000	2.092265
C	2.414282	-0.022123	1.361228
C	2.430081	-0.044046	-0.022518
C	1.212710	-0.027413	-0.678401
P	-1.687651	-0.064102	2.169535
C	-1.909010	1.447732	3.162452
C	-2.919470	2.382804	2.768953
C	-2.962516	3.433488	3.714263
C	-1.985802	3.165195	4.707374
C	-1.340194	1.949336	4.376082
Fe	-3.345277	1.621981	4.628677
C	-3.551363	0.280703	6.161627
C	-4.146988	-0.244369	4.971011
C	-5.170069	0.680145	4.585227
C	-5.204078	1.740178	5.520383
C	-4.203345	1.492625	6.494814
P	-3.817462	-1.677586	3.894979
P	-1.593053	-1.586866	3.793973
Cl	-1.311151	-3.252000	2.585407
C	1.376191	0.024231	3.601484
C	2.071648	1.300639	4.077211
C	3.731294	-0.080297	-0.792935
C	4.527904	-1.349693	-0.486425
C	-1.274634	-0.010328	-0.835746
C	-1.688989	-1.449048	-1.150915

C	-4.236151	-3.205893	4.849811
C	-3.615598	-3.669806	6.026546
C	-4.010811	-4.882308	6.573399
C	-5.001220	-5.665283	6.006425
C	-5.615297	-5.193025	4.860830
C	-5.265177	-3.984392	4.270733
C	-2.519457	-2.922931	6.760670
C	-1.214114	-3.719400	6.797526
C	-5.997352	-3.585551	2.994748
C	-5.292522	-4.178579	1.773063
C	-5.403562	-6.988614	6.618746
C	-4.237255	-7.978277	6.629631
C	-7.481920	-3.950583	2.998055
C	-5.982375	-6.803640	8.022579
C	-2.966268	-2.512792	8.164733
C	2.079695	-1.227812	4.127872
C	-1.177833	0.821704	-2.114647
C	4.570502	1.172467	-0.535598
H	-2.305354	-2.013877	6.209812
H	-5.942643	-2.501399	2.902299
H	-3.553066	2.275190	1.903045
H	-6.396979	-5.792980	4.411717
H	-1.787397	3.765677	5.580631
H	-1.321067	-4.639118	7.376623
H	-0.421591	-3.124437	7.256503
H	-0.893850	-3.990978	5.790377
H	-3.958937	2.130122	7.329315
H	-5.788291	0.581706	3.707199
H	-6.191189	-7.409059	5.986753
H	-0.573376	1.480026	4.966959
H	-5.856453	2.597584	5.484839
H	-3.635642	4.275286	3.697689
H	-3.521047	-5.227621	7.476843
H	-3.143436	-3.384522	8.797955
H	-3.888987	-1.931573	8.127834
H	-2.196219	-1.905225	8.646555
H	-2.726654	-0.143318	6.706991
H	-3.835697	-8.123745	5.625809
H	-4.559816	-8.947518	7.015482
H	-3.425026	-7.619357	7.265888
H	-4.260232	-3.831041	1.707644
H	-5.809093	-3.891272	0.854604
H	-5.278892	-5.269886	1.831217
H	-7.646364	-5.026166	2.906596
H	-7.973701	-3.479358	2.144948
H	-7.976420	-3.607754	3.908446
H	-5.230699	-6.401417	8.705725
H	-6.323131	-7.759130	8.426662
H	-6.827543	-6.113837	8.011629
H	1.211787	-0.036854	-1.761266
H	3.356539	-0.025675	1.897384
H	-2.071332	0.429372	-0.236726
H	3.475016	-0.096566	-1.856232
H	0.381082	0.017187	4.031962

H	5.432691	-1.391846	-1.096299
H	4.830961	-1.377958	0.562728
H	3.935594	-2.243556	-0.687042
H	5.476955	1.159379	-1.144307
H	4.009419	2.077320	-0.773678
H	4.873222	1.232146	0.512393
H	-2.168875	0.915914	-2.562598
H	-0.798825	1.824748	-1.911860
H	-0.531929	0.359631	-2.864031
H	-2.605523	-1.463373	-1.744816
H	-0.905415	-1.957656	-1.718238
H	-1.865729	-2.016600	-0.235850
H	2.091850	-1.223086	5.219918
H	1.565378	-2.132130	3.798667
H	3.113878	-1.283194	3.781367
H	2.090642	1.339035	5.169192
H	3.104851	1.346624	3.726978
H	1.553306	2.188424	3.711495

52
dppe SCF done: -1687.957646

C	1.46929	1.07211	1.81301
C	1.64884	1.03518	0.43171
C	1.82496	2.23844	-0.24653
C	1.83580	3.44532	0.43441
C	1.64810	3.46824	1.80704
C	1.46171	2.27826	2.49541
P	1.63747	-0.48723	-0.58335
C	3.33752	-1.13988	-0.35366
C	3.70148	-2.23735	-1.13451
C	4.95709	-2.80993	-1.01638
C	5.88119	-2.27852	-0.12859
C	5.53811	-1.17685	0.63882
C	4.27461	-0.61367	0.53101
C	0.74021	-1.70459	0.49169
C	-0.73146	-1.39367	0.76951
P	-1.75561	-1.09261	-0.75358
C	-1.61325	0.72729	-0.94490
C	-1.32360	1.22646	-2.21029
C	-1.19878	2.59222	-2.42451
C	-1.36172	3.47535	-1.37053
C	-1.64780	2.99101	-0.10148
C	-1.77204	1.62938	0.10710
C	-3.43868	-1.27951	-0.03748
C	-4.42245	-0.29694	-0.11661
C	-5.70473	-0.53386	0.35843
C	-6.02944	-1.75670	0.92126
C	-5.06398	-2.75055	0.99299
C	-3.78835	-2.51599	0.50948
H	-0.82413	-0.54796	1.45300
H	-1.17654	-2.25138	1.27714
H	1.27500	-1.84413	1.43500
H	0.81838	-2.65086	-0.05038
H	1.93685	2.22816	-1.32435

H	1.97307	4.37022	-0.11148
H	1.64368	4.41052	2.34060
H	1.31461	2.28892	3.56831
H	1.33684	0.15249	2.36933
H	2.99407	-2.64347	-1.84968
H	5.21895	-3.66481	-1.62735
H	6.86707	-2.71754	-0.04137
H	6.25648	-0.75164	1.32884
H	4.02166	0.24585	1.13870
H	-1.18200	0.53526	-3.03234
H	-0.97112	2.96343	-3.41589
H	-1.25993	4.54113	-1.53338
H	-1.76493	3.67606	0.72849
H	-2.00399	1.26729	1.10138
H	-3.05773	-3.31652	0.54994
H	-5.30754	-3.71588	1.41916
H	-7.02905	-1.93873	1.29498
H	-6.45197	0.24691	0.28800
H	-4.19139	0.66684	-0.55088

65
dppf SCF done: -3259.006097

C	-2.85100	0.72068	2.09186
C	-2.10764	1.18842	1.00963
C	-1.13673	2.15900	1.23564
C	-0.88658	2.62970	2.51697
C	-1.61567	2.13995	3.58793
C	-2.60407	1.18819	3.37160
P	-2.29354	0.58190	-0.71122
C	-4.11012	0.34561	-0.81532
C	-4.94968	1.40679	-0.47419
C	-6.32184	1.30420	-0.62574
C	-6.88223	0.14147	-1.13579
C	-6.05804	-0.91385	-1.49040
C	-4.68313	-0.81363	-1.33133
C	-1.72907	-1.14001	-0.59908
C	-0.90368	-1.74913	-1.59207
C	-0.74171	-3.11763	-1.27040
C	-1.46831	-3.37280	-0.07716
C	-2.07110	-2.16134	0.33913
Fe	-0.03563	-1.93894	0.26469
C	1.07085	-0.47266	1.20655
C	1.86584	-1.14769	0.23706
C	1.89078	-2.52780	0.60382
C	1.12474	-2.68995	1.78592
C	0.60792	-1.41885	2.15173
P	2.73041	-0.47186	-1.20666
C	2.10023	1.25472	-1.21103
C	1.11870	1.58769	-2.13888
C	0.60267	2.87399	-2.20054
C	1.07523	3.85038	-1.33947
C	2.05523	3.53184	-0.40831
C	2.56118	2.24423	-0.34299
C	4.41484	-0.21904	-0.52312

C	4.76138	-0.43578	0.80729
C	6.06296	-0.22501	1.24057
C	7.03299	0.21088	0.35239
C	6.69905	0.42788	-0.97708
C	5.40327	0.20492	-1.41085
H	-0.46250	-1.23978	-2.43384
H	-1.51968	-4.31585	0.44313
H	-0.04857	-1.20701	2.98049
H	2.39520	-3.31134	0.06055
H	-2.67564	-2.02908	1.22102
H	0.93432	-3.62283	2.29183
H	-0.14438	-3.82988	-1.81607
H	0.83748	0.57732	1.20216
H	0.75343	0.83365	-2.82536
H	-0.17124	3.10777	-2.91990
H	0.68066	4.85746	-1.39043
H	2.42896	4.29104	0.26767
H	3.32954	2.00785	0.38275
H	5.15249	0.36478	-2.45386
H	7.45224	0.76332	-1.67904
H	8.04752	0.37678	0.69238
H	6.31687	-0.39958	2.27884
H	4.01073	-0.76899	1.51289
H	-4.52601	2.32120	-0.07448
H	-6.95638	2.13608	-0.34615
H	-7.95516	0.06059	-1.25601
H	-6.48470	-1.82548	-1.89021
H	-4.05442	-1.65124	-1.60614
H	-3.63977	-0.00384	1.93004
H	-3.18699	0.81348	4.20392
H	-1.42432	2.50480	4.58936
H	-0.12089	3.37931	2.67358
H	-0.55927	2.53915	0.40104

49

7 SCF done: -2649.317856

C	1.32738	-0.60780	0.22210
C	1.52852	-1.85727	-0.43615
C	1.13289	-2.89874	0.43876
C	0.67757	-2.30728	1.64590
C	0.79127	-0.90161	1.51262
Fe	-0.45398	-1.64851	0.05288
C	-1.54998	-1.43570	-1.66541
C	-1.76199	-2.72300	-1.11297
C	-2.29404	-2.55394	0.18948
C	-2.41994	-1.16299	0.43440
C	-1.96882	-0.45264	-0.71637
P	-1.88337	1.31621	-1.15031
C	-2.47661	2.28578	0.34107
C	-1.54653	2.05172	1.53392
P	1.71918	1.06731	-0.35199
C	3.58325	1.14342	-0.13431
C	4.33378	-0.01508	-0.78719
C	-2.36943	3.75580	-0.08735

C	-3.92760	1.98763	0.72360
C	4.02323	2.46528	-0.77190
C	3.87897	1.17094	1.36734
H	-1.12944	-1.22335	-2.63598
H	-2.53119	-3.34147	0.88670
H	1.90260	-1.97847	-1.44012
H	-2.77890	-0.73032	1.35227
H	1.14000	-3.95328	0.21387
H	-1.52264	-3.66225	-1.58485
H	0.51012	-0.16746	2.25091
H	-4.21457	2.58773	1.59320
H	-4.60673	2.23749	-0.09372
H	-4.08520	0.93963	0.97501
H	-2.72075	4.40292	0.72148
H	-1.33797	4.02732	-0.31863
H	-2.98257	3.96396	-0.96806
H	-1.82852	2.72078	2.35234
H	-1.60509	1.02796	1.90322
H	-0.50675	2.25188	1.27049
H	4.95292	1.29390	1.53443
H	3.36509	1.99893	1.86102
H	3.56988	0.24151	1.85036
H	5.41287	0.12126	-0.66311
H	4.06601	-0.97124	-0.33653
H	4.12509	-0.07169	-1.85787
H	5.10008	2.60397	-0.63942
H	3.81624	2.47982	-1.84487
H	3.51488	3.31836	-0.31760
H	0.28213	-2.83354	2.49970
H	-3.11747	1.36331	-1.84947
H	1.70462	0.79371	-1.74047

55

isomer-1 of **8c**$^{+}$[$AlCl_4$]$^{-}$ SCF done: -5074.302603

C	2.47044	-1.68358	-2.52911
C	3.57690	-1.66817	-1.63940
C	3.08168	-1.63799	-0.31336
C	1.65550	-1.64078	-0.38469
C	1.28115	-1.66913	-1.76433
Fe	2.40187	0.00025	-1.32846
C	1.28062	1.66913	-1.76420
C	2.46983	1.68388	-2.52912
C	3.57641	1.66876	-1.63951
C	3.08134	1.63847	-0.31339
C	1.65514	1.64094	-0.38459
P	0.51749	1.49211	0.98456
P	1.01780	-0.00012	2.41520
P	0.51773	-1.49225	0.98438
C	0.27542	-3.13713	1.76982
C	-0.90749	-3.02286	2.73755
C	-0.05304	-4.13144	0.65010
C	1.55028	-3.55308	2.50562
C	0.27511	3.13694	1.77012
C	-0.90765	3.02251	2.73798

C	1.55004	3.55295	2.50575
C	-0.05360	4.13129	0.65051
Cl	-2.43141	-1.75123	-1.64804
Al	-3.42481	-0.00002	-0.83626
Cl	-2.43153	1.75145	-1.64745
Cl	-3.03896	-0.00053	1.29792
Cl	-5.49561	-0.00007	-1.27359
H	0.26857	-1.65090	-2.13753
H	4.61660	-1.64969	-1.92297
H	0.26799	1.65078	-2.13726
H	3.66675	-1.58154	0.59038
H	2.52312	1.67651	-3.60556
H	2.52378	-1.67608	-3.60555
H	3.66648	1.58221	0.59031
H	-0.25408	-5.10682	1.09847
H	-0.93904	-3.82838	0.08893
H	0.77724	-4.24611	-0.04813
H	-1.10178	-4.00752	3.16856
H	-0.70068	-2.32784	3.55181
H	-1.81292	-2.68776	2.22871
H	1.39218	-4.53368	2.95975
H	2.40057	-3.63438	1.82574
H	1.80704	-2.85353	3.30338
H	1.39191	4.53349	2.96002
H	1.80702	2.85334	3.30340
H	2.40020	3.63443	1.82573
H	-0.25462	5.10664	1.09898
H	0.77655	4.24607	-0.04786
H	-0.93968	3.82822	0.08948
H	-1.10198	4.00712	3.16910
H	-1.81313	2.68743	2.22919
H	-0.70079	2.32742	3.55215
H	4.61610	1.65061	-1.92316
H	-0.69771	-1.24340	0.32905
H	-0.69796	1.24318	0.32926

50

isomer-2 of **8c**$^{+}$ SCF done: -2990.4925081

C	-3.08796	-1.35453	-1.20416
C	-3.48021	-0.94862	0.09525
C	-2.73847	0.20136	0.44647
C	-1.86634	0.51221	-0.64577
C	-2.09589	-0.46442	-1.66910
Fe	-1.44214	-1.33710	0.06008
C	-0.02189	-2.74908	-0.38867
C	-0.90600	-3.27342	0.58026
C	-0.86428	-2.43426	1.72121
C	0.04101	-1.37815	1.46839
C	0.56943	-1.56371	0.15089
P	1.75434	-0.56797	-0.73793
P	1.19024	1.24271	-1.73780
P	-0.63270	1.79765	-0.80221
C	-0.61417	2.92920	0.66344
C	0.43078	4.01003	0.35834

C	-1.99602	3.58384	0.78967
C	-0.22604	2.18914	1.94183
C	3.33982	-0.48417	0.20556
C	4.40122	0.05623	-0.76044
C	3.20288	0.44938	1.40683
C	3.69603	-1.90535	0.65231
H	-1.57889	-0.51465	-2.61387
H	-4.20188	-1.44363	0.72434
H	0.16108	-3.15446	-1.37096
H	-2.81285	0.72292	1.38446
H	-1.52145	-4.14986	0.46029
H	-3.45921	-2.21431	-1.73754
H	0.27825	-0.57717	2.14676
H	-1.97170	4.29120	1.62078
H	-2.25781	4.14440	-0.10936
H	-2.79238	2.86857	0.98726
H	0.42491	4.73684	1.17254
H	1.43497	3.59422	0.27649
H	0.20772	4.54738	-0.56571
H	-0.15145	2.90950	2.75841
H	-0.95777	1.43581	2.22935
H	0.74852	1.71020	1.84024
H	4.16837	0.52133	1.91058
H	2.91412	1.45588	1.09884
H	2.48378	0.07557	2.13721
H	4.66888	-1.88522	1.14694
H	2.96857	-2.30714	1.35829
H	3.77258	-2.59136	-0.19387
H	5.35949	0.09899	-0.23981
H	4.52745	-0.58976	-1.63166
H	4.16384	1.06438	-1.10422
H	-1.44321	-2.56084	2.62142
H	-1.12807	2.66697	-1.78761
H	2.10830	-1.43097	-1.78635

66

8b⁺ SCF done: -3600.1934197

C	1.05030	1.40145	-1.95993
C	1.77653	1.41845	-0.77136
C	2.53576	2.54157	-0.45172
C	2.56911	3.62582	-1.31325
C	1.85120	3.59729	-2.49878
C	1.09362	2.48231	-2.82189
P	1.66332	0.06550	0.42618
C	3.18150	0.09957	1.39984
C	3.25116	0.79004	2.60742
C	4.44423	0.83202	3.31023
C	5.56778	0.19135	2.81134
C	5.50169	-0.49508	1.60763
C	4.31276	-0.54329	0.90107
C	1.68625	-1.47665	-0.47986
C	1.85395	-1.72437	-1.87845
C	1.74026	-3.11746	-2.08626

C	1.52416	-3.74117	-0.83159
C	1.48909	-2.73965	0.16359
Fe	0.01631	-2.37209	-1.19785
C	-1.60502	-1.27441	-0.61134
C	-1.72936	-2.62865	-0.16946
C	-1.73244	-3.46317	-1.31088
C	-1.59977	-2.64514	-2.46094
C	-1.52193	-1.29645	-2.04052
P	-1.56367	0.11878	0.52496
C	-1.83946	1.63126	-0.43136
C	-1.18019	2.80005	-0.06935
C	-1.43786	3.97973	-0.74703
C	-2.35534	3.99719	-1.78506
C	-3.03148	2.83679	-2.13636
C	-2.78291	1.65694	-1.45726
P	0.09053	0.14883	1.87254
C	-3.02232	-0.04785	1.57969
C	-3.06864	0.62986	2.79611
C	-4.21087	0.57225	3.57579
C	-5.31228	-0.15126	3.14395
C	-5.27290	-0.81750	1.92897
C	-4.13224	-0.76747	1.14496
H	1.31503	-2.88511	1.21741
H	1.78971	-3.61464	-3.04128
H	-1.54578	-2.98837	-3.48111
H	-1.79553	-2.94813	0.85818
H	2.03371	-0.98595	-2.64073
H	-1.79258	-4.53914	-1.30233
H	1.37528	-4.79581	-0.66679
H	-1.42988	-0.43969	-2.68664
H	-0.45660	2.78160	0.73566
H	-0.91622	4.88456	-0.46489
H	-2.55440	4.91880	-2.31656
H	-3.76296	2.85374	-2.93362
H	-3.33446	0.76370	-1.72195
H	-2.21780	1.20644	3.13792
H	-4.24029	1.09542	4.52232
H	-6.20403	-0.19465	3.75553
H	-6.13213	-1.38046	1.58906
H	-4.11193	-1.29501	0.20031
H	2.37881	1.29639	3.00145
H	4.49384	1.36517	4.25032
H	6.49798	0.22429	3.36352
H	6.37743	-0.99781	1.21916
H	4.26633	-1.08770	-0.03371
H	3.11003	2.57228	0.46503
H	3.16612	4.49178	-1.05931
H	1.88332	4.44422	-3.17173
H	0.52544	2.45747	-3.74238
H	0.43872	0.54833	-2.21161

71

8b$^+$[$AlCl_4$]$^-$ SCF done: -5683.968562

C	1.32538	-1.09114	2.49840

C	0.49690	-1.76759	1.60438
C	-0.60776	-2.45981	2.08939
C	-0.87644	-2.47330	3.44868
C	-0.04426	-1.80610	4.33130
C	1.06117	-1.11678	3.85447
P	0.74600	-1.66027	-0.18621
C	-0.03989	-3.10880	-0.91620
C	-1.36467	-3.04470	-1.34022
C	-1.97156	-4.17529	-1.86369
C	-1.26459	-5.36266	-1.96160
C	0.05579	-5.42740	-1.53637
C	0.67053	-4.30390	-1.01329
C	2.50937	-1.83450	-0.48347
C	3.58039	-2.08552	0.42815
C	4.79001	-2.05726	-0.30378
C	4.48047	-1.81152	-1.66604
C	3.07925	-1.67214	-1.78421
Fe	3.78990	-0.25251	-0.50115
C	2.69283	1.46067	-0.23456
C	3.45381	1.53084	-1.44146
C	4.82401	1.42357	-1.10649
C	4.92584	1.27427	0.30011
C	3.61848	1.29789	0.84311
P	0.88899	1.53845	-0.22274
C	0.34416	1.81890	1.47768
C	-0.85250	1.24635	1.88987
C	-1.33949	1.50044	3.16112
C	-0.63188	2.32227	4.02128
C	0.55551	2.91303	3.60536
C	1.04007	2.67346	2.33220
P	-0.10148	-0.02725	-1.26344
C	0.45130	3.03646	-1.13418
C	-0.84454	3.16119	-1.62905
C	-1.22830	4.33218	-2.26105
C	-0.32846	5.37712	-2.39853
C	0.96006	5.25713	-1.89806
C	1.35202	4.09095	-1.26318
Cl	-3.55891	-1.16124	1.05906
Al	-4.47909	0.18470	-0.38383
Cl	-3.75590	-0.34705	-2.34754
Cl	-6.60003	0.02092	-0.27549
Cl	-3.82403	2.18673	0.09433
H	2.52619	-1.44668	-2.68156
H	5.77738	-2.18128	0.11044
H	5.83679	1.14430	0.86120
H	3.04070	1.63069	-2.43232
H	3.48566	-2.25721	1.48660
H	5.64435	1.42176	-1.80546
H	5.19238	-1.71072	-2.46900
H	3.36801	1.21713	1.88740
H	-1.41401	0.60169	1.22647
H	-2.27726	1.05200	3.46085
H	-1.01088	2.51965	5.01614
H	1.09595	3.57482	4.27010

H	1.94823	3.16399	2.00484
H	-1.57079	2.36658	-1.50646
H	-2.23973	4.42179	-2.63428
H	-0.63255	6.29122	-2.89247
H	1.66241	6.07442	-1.99977
H	2.35873	4.00650	-0.87442
H	-1.93760	-2.12856	-1.26054
H	-3.00078	-4.11414	-2.19153
H	-1.74203	-6.24354	-2.37182
H	0.60832	-6.35475	-1.61447
H	1.70214	-4.35648	-0.68781
H	-1.27635	-2.97228	1.41244
H	-1.75020	-2.99877	3.81005
H	-0.26114	-1.81431	5.39180
H	1.70670	-0.57976	4.53715
H	2.17033	-0.52688	2.13541

98

3⁺ SCF done: -3844.537833

C	-1.83218	2.91781	-0.47881
C	-1.38345	1.61821	-0.87625
C	-0.84784	1.71935	-2.20336
C	-0.95983	3.07060	-2.60116
C	-1.56293	3.80327	-1.54631
P	-1.48905	0.13625	0.07522
P	-0.01889	-0.77941	1.05244
P	1.68952	0.51778	1.20657
C	1.62338	2.07325	0.29718
C	2.16733	2.40606	-0.97983
C	2.00747	3.79698	-1.17856
C	1.37502	4.34091	-0.03149
C	1.14430	3.28804	0.88374
Fe	0.17441	2.89039	-0.87747
C	-3.12449	-0.56936	0.16210
C	-3.96109	-0.24899	1.24501
C	-5.21697	-0.82820	1.27932
C	-5.66557	-1.68038	0.27565
C	-4.82340	-1.94599	-0.79428
C	-3.54855	-1.40675	-0.87968
C	-3.52839	0.68546	2.35708
C	-4.55968	1.77835	2.63658
C	-2.68652	-1.70264	-2.08959
C	-3.27060	-1.06136	-3.34927
C	-7.04538	-2.29418	0.34237
C	-8.13423	-1.21974	0.30709
C	3.07207	-0.47715	0.54609
C	3.08750	-1.07917	-0.72750
C	4.19842	-1.83007	-1.09080
C	5.29276	-1.99068	-0.25979
C	5.26623	-1.36718	0.97999
C	4.18445	-0.61770	1.41007
C	1.95985	-0.97668	-1.74229
C	2.45038	-0.55224	-3.13043

C	6.48339	-2.81555	-0.69194
C	6.69136	-4.01775	0.23128
C	4.26518	0.08636	2.75582
C	5.12901	-0.63484	3.78809
C	1.19359	-2.29739	-1.85422
C	7.74927	-1.96108	-0.78332
C	4.75236	1.52686	2.55974
C	-2.44439	-3.20030	-2.27009
C	-7.19350	-3.19622	1.56932
C	-3.17781	-0.09896	3.62165
H	4.21432	-2.30986	-2.06223
H	-2.61313	1.19389	2.03085
H	3.25580	0.13268	3.16936
H	6.12055	-1.47458	1.63427
H	1.26486	-0.20775	-1.40417
H	-1.70334	-1.24619	-1.92242
H	6.26527	-3.19695	-1.69328
H	2.62118	1.71717	-1.66926
H	-5.17664	-2.59428	-1.58757
H	1.09897	5.37303	0.11056
H	-4.05024	-0.64126	3.99129
H	-2.83907	0.57466	4.41016
H	-2.38939	-0.82926	3.42934
H	-1.75823	4.86311	-1.54542
H	5.75760	1.53290	2.13282
H	4.78466	2.05253	3.51541
H	4.10388	2.09116	1.88571
H	1.84785	-3.09065	-2.21988
H	0.36236	-2.20059	-2.55584
H	0.79203	-2.62507	-0.89388
H	-0.43029	0.90982	-2.77993
H	-7.16343	-2.91845	-0.54716
H	0.68161	3.37486	1.85413
H	3.10907	0.31567	-3.09183
H	1.59979	-0.30711	-3.76996
H	3.00109	-1.35398	-3.62364
H	-0.62169	3.47807	-3.53977
H	7.60732	-1.11213	-1.45361
H	8.58212	-2.55731	-1.15877
H	8.03446	-1.57329	0.19703
H	2.30041	4.34434	-2.05963
H	-5.87295	-0.60605	2.11181
H	-5.47683	1.36706	3.06051
H	-4.82430	2.31788	1.72581
H	-4.16082	2.49251	3.35845
H	-2.27837	3.17246	0.46888
H	4.85574	-1.68695	3.87851
H	4.99681	-0.16553	4.76363
H	6.19223	-0.57554	3.54788
H	-6.42589	-3.97102	1.58788
H	-8.16962	-3.68294	1.56355
H	-7.11592	-2.62079	2.49442
H	6.93959	-3.69728	1.24542
H	7.51348	-4.63613	-0.13182

H	5.79499	-4.63733	0.28364
H	-1.99100	-3.63727	-1.37922
H	-1.77782	-3.37706	-3.11562
H	-3.37634	-3.73150	-2.46952
H	-4.24322	-1.49223	-3.59313
H	-2.60932	-1.22804	-4.20141
H	-3.40248	0.01431	-3.22020
H	-8.08640	-0.57981	1.19091
H	-9.12117	-1.68372	0.28748
H	-8.03784	-0.58527	-0.57505

103

3$^+$[$AlCl_4$]$^-$ SCF done: -5928.317488

C	-1.37883	-2.05302	2.84447
C	-1.51820	-0.93472	1.95285
C	-1.71505	0.24053	2.74377
C	-1.68650	-0.15238	4.09905
C	-1.48628	-1.55641	4.16252
P	-1.43939	-0.99084	0.18651
P	0.19758	-1.35351	-0.89364
P	1.83749	-1.69968	0.42318
C	1.76083	-0.87008	2.01950
C	1.58875	0.50133	2.38910
C	1.60511	0.57554	3.79921
C	1.79085	-0.73130	4.32009
C	1.89260	-1.62549	3.23010
Fe	0.08531	-0.67098	3.15110
C	-3.00740	-1.22904	-0.65131
C	-3.62368	-2.49325	-0.62261
C	-4.84455	-2.63147	-1.25894
C	-5.45667	-1.57238	-1.91907
C	-4.81957	-0.34231	-1.93077
C	-3.59889	-0.13543	-1.30205
C	-3.02167	-3.69236	0.08213
C	-3.85609	-4.09225	1.29966
C	-2.92812	1.21719	-1.36871
C	-3.88455	2.38819	-1.16191
C	-6.79116	-1.75643	-2.60636
C	-7.88727	-2.12322	-1.60411
C	3.29171	-1.14242	-0.52870
C	3.41640	0.13254	-1.10887
C	4.54924	0.40285	-1.87039
C	5.55983	-0.51995	-2.05593
C	5.43057	-1.76211	-1.44808
C	4.32190	-2.10090	-0.69255
C	2.41503	1.25833	-0.94469
C	3.05583	2.45297	-0.23358
C	6.77649	-0.18264	-2.88826
C	6.89649	-1.10541	-4.10237
C	4.29814	-3.45583	-0.00209
C	4.98260	-4.57081	-0.79026
C	1.79226	1.68141	-2.27619
C	8.05336	-0.20743	-2.04610
C	4.90538	-3.33365	1.39964

C	-2.14943	1.36412	-2.67711
C	-6.70249	-2.78714	-3.73322
C	-2.81169	-4.86749	-0.87309
Cl	-0.49103	2.77905	0.71573
Al	-0.63915	4.88402	0.09877
Cl	-0.62032	4.89035	-2.05023
Cl	-2.49039	5.63124	0.85775
Cl	1.07207	5.88231	0.89536
H	4.64531	1.37935	-2.33003
H	-2.02964	-3.41113	0.44338
H	3.25475	-3.75202	0.11802
H	6.22296	-2.48858	-1.57124
H	1.59608	0.91571	-0.31302
H	-2.19708	1.27276	-0.55605
H	6.64056	0.83766	-3.25690
H	1.41848	1.33382	1.72792
H	-5.29089	0.48751	-2.44242
H	1.82324	-1.00047	5.36329
H	-3.76145	-5.24494	-1.25609
H	-2.31226	-5.68921	-0.35675
H	-2.19818	-4.57421	-1.72624
H	-1.39817	-2.14362	5.06207
H	5.95504	-3.03815	1.33471
H	4.85175	-4.28811	1.92773
H	4.38836	-2.58074	1.99764
H	2.54016	2.10836	-2.94710
H	1.02755	2.44003	-2.10427
H	1.33035	0.83445	-2.78933
H	-1.81106	1.24551	2.36411
H	-7.05687	-0.79510	-3.05373
H	2.02098	-2.69474	3.28679
H	3.51394	2.15166	0.71045
H	2.30709	3.21697	-0.02484
H	3.83189	2.90886	-0.85093
H	-1.77307	0.51353	4.94192
H	7.97398	0.46448	-1.19040
H	8.91166	0.10095	-2.64584
H	8.25511	-1.21227	-1.66783
H	1.46126	1.47603	4.37408
H	-5.33656	-3.59676	-1.23981
H	-4.85363	-4.41866	0.99918
H	-3.97011	-3.25559	1.99063
H	-3.38080	-4.91756	1.83384
H	-1.21075	-3.07781	2.55751
H	4.62061	-4.61472	-1.81840
H	4.77916	-5.53236	-0.31591
H	6.06743	-4.45072	-0.81566
H	-5.93103	-2.51697	-4.45560
H	-7.65579	-2.85695	-4.25995
H	-6.46525	-3.77921	-3.34219
H	7.05762	-2.14116	-3.79456
H	7.74195	-0.80772	-4.72531
H	5.99291	-1.07296	-4.71278
H	-1.40946	0.56887	-2.79581

H	-1.63044	2.32300	-2.69339
H	-2.82675	1.32022	-3.53369
H	-4.57132	2.50516	-2.00298
H	-3.31541	3.31382	-1.07636
H	-4.47183	2.26894	-0.25032
H	-7.68768	-3.09173	-1.13973
H	-8.85458	-2.18778	-2.10538
H	-7.96173	-1.37839	-0.81068

100

5^{+} SCF done: -4646.181563

C	3.74060	-0.45653	1.35642
C	3.04802	-0.58630	0.14131
C	3.60997	-1.31981	-0.92516
C	4.78892	-2.00719	-0.69236
C	5.44468	-1.97598	0.53046
C	4.91630	-1.17308	1.52652
P	1.42525	0.18576	-0.07081
P	0.00005	-0.08637	1.58248
Cl	0.00007	1.58412	2.86143
C	3.06575	-1.32536	-2.34232
C	2.72587	-2.73166	-2.83539
C	6.71927	-2.75668	0.75226
C	6.48709	-4.25927	0.58326
C	3.30852	0.47765	2.46521
C	2.95964	-0.26938	3.75196
C	1.60707	1.86654	-0.65242
C	1.62795	2.22833	-2.03745
C	1.68520	3.63904	-2.11073
C	1.70503	4.15590	-0.79058
C	1.66550	3.07171	0.11606
Fe	-0.00001	3.00968	-1.08466
C	-1.68524	3.63900	-2.11072
C	-1.62796	2.22829	-2.03743
C	-1.60707	1.86651	-0.65240
C	-1.66551	3.07169	0.11607
C	-1.70507	4.15587	-0.79058
P	-1.42522	0.18575	-0.07074
P	0.00000	-1.02388	-1.12311
C	-3.04799	-0.58632	0.14139
C	-3.74055	-0.45664	1.35652
C	-4.91625	-1.17320	1.52658
C	-5.44465	-1.97602	0.53048
C	-4.78892	-2.00713	-0.69236
C	-3.60997	-1.31975	-0.92513
C	-3.30849	0.47749	2.46534
C	-2.95972	-0.26958	3.75211
C	-6.71925	-2.75673	0.75224
C	-7.84061	-2.26081	-0.16332
C	-3.06583	-1.32514	-2.34232
C	-4.03860	-0.61345	-3.28762
C	-4.35923	1.56323	2.71126
C	-6.48709	-4.25931	0.58312
C	-2.72599	-2.73138	-2.83558

C	4.03848	-0.61380	-3.28777
C	4.35929	1.56336	2.71114
C	7.84063	-2.26084	-0.16334
H	-5.44714	-1.09210	2.46772
H	2.14297	-0.74484	-2.37374
H	-2.14304	-0.74463	-2.37372
H	-5.22267	-2.57754	-1.50444
H	-2.41491	1.00893	2.13893
H	2.41497	1.00910	2.13874
H	-7.02925	-2.58013	1.78561
H	-1.65130	3.14249	1.19033
H	5.44720	-1.09192	2.46764
H	-1.68716	4.22048	-3.01809
H	3.62087	-3.35055	-2.91632
H	2.26677	-2.68351	-3.82394
H	2.03156	-3.23338	-2.15980
H	1.68710	4.22053	-3.01809
H	-4.98579	-1.15002	-3.36056
H	-3.61195	-0.55340	-4.29040
H	-4.25342	0.39936	-2.94212
H	-3.83889	-0.76826	4.16338
H	-2.58509	0.42473	4.50520
H	-2.19770	-1.03228	3.57964
H	1.65129	3.14251	1.19032
H	7.02929	-2.58001	1.78562
H	-1.57412	1.54631	-2.87055
H	-4.59133	2.10157	1.79100
H	-3.98959	2.27972	3.44674
H	-5.28761	1.14038	3.09770
H	1.72445	5.19879	-0.51950
H	-8.01693	-1.19230	-0.03213
H	-8.76905	-2.78968	0.05609
H	-7.59900	-2.43726	-1.21383
H	-1.72451	5.19877	-0.51950
H	5.22263	-2.57768	-1.50439
H	4.98566	-1.15039	-3.36072
H	4.25335	0.39904	-2.94241
H	3.61176	-0.55386	-4.29053
H	1.57412	1.54636	-2.87058
H	-2.03164	-3.23319	-2.16010
H	-2.26694	-2.68311	-3.82415
H	-3.62100	-3.35026	-2.91655
H	5.69742	-4.61609	1.24581
H	7.40010	-4.81040	0.81240
H	6.20233	-4.50167	-0.44298
H	-6.20234	-4.50163	-0.44314
H	-7.40010	-4.81044	0.81224
H	-5.69741	-4.61618	1.24563
H	2.19757	-1.03202	3.57946
H	2.58501	0.42496	4.50502
H	3.83875	-0.76813	4.16328
H	5.28763	1.14050	3.09766
H	3.98963	2.27989	3.44657
H	4.59146	2.10165	1.79087

H	7.59900	-2.43737	-1.21384
H	8.76907	-2.78970	0.05609
H	8.01696	-1.19233	-0.03223

99

4^{2+} SCF done: -4185.608037

C	3.65646	-0.72436	1.38916
C	3.05619	-0.53947	0.12692
C	3.65530	-1.01852	-1.05938
C	4.80154	-1.77839	-0.93066
C	5.38118	-2.05094	0.30498
C	4.80573	-1.49498	1.43995
P	1.50742	0.32166	0.02582
P	0.00001	-0.60869	1.29381
P	-0.00001	-0.87693	-0.99248
P	-1.50742	0.32166	0.02585
C	-3.05619	-0.53948	0.12694
C	-3.65645	-0.72441	1.38918
C	-4.80571	-1.49504	1.43995
C	-5.38117	-2.05097	0.30497
C	-4.80154	-1.77837	-0.93068
C	-3.65530	-1.01848	-1.05937
C	-3.16617	-0.06083	2.66195
C	-2.81138	-1.07412	3.75078
C	-6.63035	-2.89215	0.40351
C	-7.80799	-2.21032	-0.29872
C	-3.16243	-0.67097	-2.45151
C	-4.18604	0.20779	-3.17711
C	3.16241	-0.67106	-2.45152
C	2.80125	-1.91117	-3.26991
C	6.63036	-2.89212	0.40354
C	6.39317	-4.30192	-0.14301
C	3.16621	-0.06073	2.66192
C	2.81141	-1.07397	3.75078
C	-1.59114	2.06969	-0.17942
C	-1.64030	2.76770	-1.43187
C	-1.69850	4.14732	-1.13659
C	-1.69741	4.31237	0.27145
C	-1.63861	3.03828	0.87763
Fe	-0.00000	3.26635	-0.32043
C	1.69748	4.31238	0.27124
C	1.69843	4.14726	-1.13679
C	1.64019	2.76763	-1.43200
C	1.59113	2.06969	-0.17951
C	1.63872	3.03831	0.87749
C	-4.18837	0.96737	3.15658
C	-6.39319	-4.30191	-0.14316
C	-2.80130	-1.91104	-3.26996
C	4.18602	0.20765	-3.17718
C	4.18843	0.96749	3.15649
C	7.80799	-2.21036	-0.29878
H	-5.28630	-1.65767	2.39695
H	2.25508	-0.06296	-2.36379
H	-2.25508	-0.06288	-2.36375

H	-5.27669	-2.15905	-1.82597
H	-2.25629	0.50652	2.43644
H	2.25633	0.50663	2.43639
H	-6.87793	-2.98019	1.46410
H	-1.62355	2.83257	1.93580
H	5.28634	-1.65757	2.39695
H	-1.71806	4.94201	-1.86467
H	3.68256	-2.52412	-3.46140
H	2.38839	-1.62111	-4.23672
H	2.07099	-2.53940	-2.75588
H	1.71792	4.94193	-1.86490
H	-5.11649	-0.33418	-3.34890
H	-3.79782	0.51438	-4.14934
H	-4.42272	1.10253	-2.59902
H	-3.69519	-1.62154	4.07968
H	-2.39881	-0.56513	4.62257
H	-2.08243	-1.80801	3.40123
H	1.62376	2.83265	1.93566
H	6.87798	-2.98009	1.46413
H	-1.62692	2.32288	-2.41382
H	-4.41932	1.70212	2.38350
H	-3.80124	1.49245	4.03085
H	-5.12145	0.48420	3.44775
H	1.71629	5.25390	0.79583
H	-7.98606	-1.21000	0.09799
H	-8.71532	-2.79764	-0.15745
H	-7.63386	-2.12551	-1.37357
H	-1.71617	5.25387	0.79607
H	5.27668	-2.15911	-1.82595
H	5.11647	-0.33433	-3.34896
H	4.42272	1.10242	-2.59913
H	3.79780	0.51420	-4.14942
H	1.62670	2.32277	-2.41393
H	-2.07104	-2.53930	-2.75596
H	-2.38844	-1.62095	-4.23676
H	-3.68261	-2.52397	-3.46146
H	5.56093	-4.79302	0.36299
H	7.28510	-4.91133	0.00267
H	6.17910	-4.28285	-1.21378
H	-6.17916	-4.28277	-1.21394
H	-7.28512	-4.91133	0.00252
H	-5.56093	-4.79305	0.36277
H	2.08245	-1.80786	3.40126
H	2.39885	-0.56494	4.62255
H	3.69521	-1.62139	4.07970
H	5.12149	0.48430	3.44768
H	3.80131	1.49259	4.03075
H	4.41938	1.70219	2.38339
H	7.63382	-2.12563	-1.37363
H	8.71532	-2.79767	-0.15749
H	7.98607	-1.21001	0.09785

109

5⁺ $[Al_2Cl_7]^-$ SCF done: -8353.285864

C	3.74612	0.74887	1.40923
C	3.26951	0.35389	0.14866
C	3.65822	1.05361	-1.00969
C	4.38615	2.22302	-0.85508
C	4.76886	2.69943	0.38866
C	4.47917	1.91915	1.49673
P	2.07210	-0.99044	-0.00848
C	2.91519	-2.54965	-0.28460
C	3.22895	-3.06961	-1.58005
C	3.79778	-4.35259	-1.40427
C	3.84306	-4.63358	-0.01461
C	3.30935	-3.52479	0.68302
Fe	1.89065	-4.26319	-0.60823
C	0.67829	-5.90127	-0.22994
C	0.22590	-4.76707	0.48434
C	-0.05748	-3.73861	-0.46657
C	0.21035	-4.26290	-1.76974
C	0.66572	-5.59322	-1.61448
P	-0.56054	-2.04217	-0.17123
P	0.43772	-0.52900	-1.31468
C	3.44779	0.53047	-2.41874
C	2.88977	1.57116	-3.38619
C	5.45219	4.03427	0.57604
C	4.39781	5.09401	0.91689
C	3.54289	-0.06467	2.66834
C	4.88266	-0.53190	3.24248
C	-2.36342	-1.92666	-0.16200
C	-3.04092	-1.50441	-1.31958
C	-4.39612	-1.22438	-1.22098
C	-5.10468	-1.39001	-0.04364
C	-4.43063	-1.91941	1.04710
C	-3.07686	-2.19599	1.01945
C	-2.41054	-1.42091	-2.69644
C	-3.01137	-2.48773	-3.61725
C	-2.44859	-2.81505	2.24825
C	-3.02588	-4.20655	2.51659
C	-6.55803	-0.99987	0.10239
C	-7.34360	-1.02427	-1.20463
P	0.46581	-1.02599	1.48435
Cl	0.94412	-2.41227	3.02977
C	-2.55980	-1.91077	3.47526
C	-6.65153	0.37982	0.76402
C	-2.51368	-0.02435	-3.30566
C	4.76140	-0.06795	-2.93425
C	2.71495	0.68085	3.71478
C	6.29979	4.47337	-0.61385
Cl	-2.45144	1.29252	1.16910
Al	-3.56117	2.97275	0.49283
Cl	-2.05315	4.70784	0.42331
Al	-0.01999	4.10424	-0.43378
Cl	-0.45210	3.00544	-2.19801
Cl	-4.98831	3.66477	1.89377
Cl	-4.33038	2.73506	-1.46878
Cl	0.98024	2.97735	1.06432

Cl	0.91998	5.96551	-0.81257
H	4.82640	2.24863	2.46935
H	-1.34961	-1.66397	-2.61742
H	2.72501	-0.28619	-2.39165
H	4.66716	2.77381	-1.74194
H	3.00742	-0.97714	2.40667
H	-1.38780	-2.97001	2.05528
H	6.11608	3.93038	1.44020
H	3.18613	-3.44261	1.74949
H	-4.98552	-2.10616	1.95950
H	4.11543	-5.01218	-2.19517
H	-3.55038	0.23785	-3.52200
H	-1.95421	0.02014	-4.24192
H	-2.12074	0.73789	-2.63332
H	0.97519	-6.24836	-2.41231
H	5.52852	0.70398	-3.02088
H	4.61475	-0.51000	-3.92217
H	5.13749	-0.84090	-2.26110
H	3.24859	1.56269	4.07391
H	2.51477	0.03436	4.57069
H	1.76219	1.02144	3.30668
H	0.14495	-4.68083	1.55443
H	-7.01081	-1.72966	0.78154
H	3.03115	-2.58171	-2.52037
H	5.46136	-1.07859	2.49593
H	4.71317	-1.18845	4.09805
H	5.48478	0.31180	3.58372
H	0.99889	-6.83191	0.20905
H	7.01337	3.70191	-0.90966
H	6.85970	5.37367	-0.35711
H	5.67870	4.71785	-1.47821
H	4.20113	-5.54476	0.43611
H	-4.90943	-0.86574	-2.10238
H	-4.07396	-2.30707	-3.78832
H	-2.90540	-3.48643	-3.18845
H	-2.50941	-2.47124	-4.58676
H	0.11557	-3.72057	-2.69677
H	1.97361	2.02047	-3.00407
H	2.66621	1.10173	-4.34591
H	3.60760	2.37103	-3.57516
H	-6.09626	0.41670	1.70198
H	-7.69246	0.63474	0.97081
H	-6.24159	1.14604	0.10362
H	3.69845	5.22454	0.08890
H	4.87563	6.05493	1.11707
H	3.81315	4.80773	1.79179
H	-2.15591	-0.91685	3.27692
H	-2.01717	-2.34526	4.31626
H	-3.60177	-1.78508	3.77426
H	-4.08852	-4.15301	2.75942
H	-2.51239	-4.66993	3.36157
H	-2.91191	-4.85362	1.64494
H	-7.01301	-0.23278	-1.88063
H	-8.40209	-0.85359	-1.00340

H	-7.24537	-1.98202	-1.71942

109

4^{2+} 2$[AlCl_4]^-$ SCF done: -8353.242342

C	0.06420	4.74404	2.23656
C	-0.16835	3.35872	2.39468
C	0.22976	2.71680	1.17814
C	0.72915	3.71916	0.28859
C	0.61333	4.96355	0.94742
Fe	-1.22867	4.02257	0.78377
C	-2.27783	3.85012	-0.95594
C	-2.68150	2.88725	0.02401
C	-3.18663	3.59411	1.16252
C	-3.06779	4.97572	0.88783
C	-2.51142	5.13236	-0.40777
P	-2.59961	1.12506	-0.13834
C	-4.10876	0.44142	-0.79848
C	-5.11998	0.01118	0.07975
C	-6.19755	-0.66221	-0.46441
C	-6.31023	-0.90007	-1.82782
C	-5.34092	-0.37828	-2.67006
C	-4.23238	0.29940	-2.19129
C	-5.12067	0.29914	1.56820
C	-6.23563	1.28937	1.91616
C	-7.44900	-1.72376	-2.38018
C	-8.81532	-1.14905	-2.00542
C	-3.24946	0.87970	-3.18977
C	-3.91044	1.98959	-4.01125
P	0.17829	0.96713	0.88985
P	-1.63698	-0.16828	1.34072
P	-0.83931	0.01328	-0.78742
C	1.69352	0.14045	1.26440
C	1.90121	-0.45468	2.52388
C	3.06145	-1.18276	2.69368
C	4.00508	-1.32632	1.68047
C	3.79335	-0.67361	0.47529
C	2.65877	0.07944	0.23919
C	0.95568	-0.29262	3.69715
C	0.37338	-1.63093	4.15106
C	2.56646	0.87203	-1.04794
C	3.54842	2.04578	-0.99811
C	5.24930	-2.15518	1.88245
C	4.88663	-3.62720	2.08999
C	-5.20678	-0.97429	2.40840
C	-7.30985	-3.18061	-1.92898
C	-2.62583	-0.19383	-4.08285
C	2.76344	0.00542	-2.29109
C	6.10087	-1.61312	3.03086
C	1.63847	0.45042	4.84820
Cl	0.37291	-2.90682	0.21714
Al	-1.57121	-3.87536	0.24162
Cl	-1.48545	-5.89497	-0.36308
Cl	-2.39273	-3.61463	2.22021
Cl	-2.82388	-2.69713	-1.10587

Cl	7.03634	0.83922	0.12062
Al	7.31479	0.03850	-1.87969
Cl	6.31372	1.34624	-3.27657
Cl	6.31306	-1.88279	-1.93870
Cl	9.38705	-0.15691	-2.32968
H	4.55063	-0.75113	-0.29304
H	-4.18164	0.79483	1.83061
H	0.11806	0.34207	3.38714
H	3.24262	-1.65827	3.64981
H	1.56251	1.30975	-1.12397
H	-2.43179	1.35668	-2.64190
H	5.83785	-2.07583	0.96693
H	1.11445	3.55419	-0.70386
H	-5.44696	-0.52237	-3.73846
H	-0.15765	5.50423	2.96748
H	-6.16741	-1.47200	2.26685
H	-5.11486	-0.73334	3.46877
H	-4.42329	-1.68671	2.14672
H	-3.33505	5.77353	1.56118
H	2.47063	-0.12958	5.24912
H	0.92973	0.62129	5.66096
H	2.03317	1.41361	4.51964
H	3.77457	-0.39950	-2.33806
H	2.61809	0.60502	-3.19095
H	2.06267	-0.83246	-2.30446
H	-1.85139	3.63368	-1.92200
H	-7.36412	-1.70070	-3.46984
H	-0.58228	2.87138	3.26276
H	3.37044	2.67310	-0.12126
H	3.45813	2.65517	-1.89951
H	4.57755	1.69381	-0.94777
H	-2.28167	6.06928	-0.88810
H	6.37530	-0.57313	2.85211
H	7.02067	-2.19358	3.11431
H	5.57602	-1.68006	3.98795
H	0.88199	5.91922	0.52815
H	-6.97201	-1.02490	0.19953
H	-7.21722	0.85839	1.71305
H	-6.14747	2.20853	1.33338
H	-6.19686	1.54434	2.97688
H	-3.56665	3.15046	2.06845
H	-0.11176	-2.16297	3.33163
H	-0.35881	-1.47993	4.94643
H	1.15974	-2.27931	4.54061
H	-6.33543	-3.58815	-2.19995
H	-8.08402	-3.79497	-2.39121
H	-7.41567	-3.26504	-0.84519
H	4.31572	-3.77162	3.01116
H	5.79504	-4.22710	2.16065
H	4.29088	-4.00743	1.25932
H	-2.18054	-0.99677	-3.49416
H	-1.85506	0.24461	-4.71844
H	-3.37632	-0.64313	-4.73493
H	-4.72564	1.59436	-4.61911

H	-3.18232	2.44360	-4.68572
H	-4.32065	2.76913	-3.36629
H	-8.98199	-1.18894	-0.92682
H	-9.60847	-1.72804	-2.48058
H	-8.91228	-0.11035	-2.32530

34

PPh_3 SCF done: -1036.310869

P	-0.13110	-0.09434	-1.02960
C	-1.88011	-0.38749	-0.46869
C	1.22791	-1.19948	-0.40400
C	0.50606	1.48180	-0.27501
C	2.54830	-0.98881	-0.81897
C	3.57121	-1.82063	-0.34809
C	3.27373	-2.86312	0.53775
C	1.95335	-3.07379	0.95272
C	0.93044	-2.24197	0.48184
H	2.77969	-0.17790	-1.50803
H	4.59828	-1.65676	-0.67088
H	4.06941	-3.51016	0.90403
H	1.72195	-3.88470	1.64178
H	-0.09664	-2.40584	0.80463
C	1.80826	1.91441	-0.55273
C	2.28785	3.10075	0.01524
C	1.46524	3.85449	0.86093
C	0.16304	3.42188	1.13865
C	-0.31655	2.23554	0.57068
H	2.44814	1.32811	-1.21055
H	3.30077	3.43726	-0.20078
H	1.83828	4.77729	1.30273
H	-0.47684	4.00818	1.79648
H	-1.32947	1.89903	0.78671
C	-2.91160	0.43762	-0.93262
C	-4.22807	0.21697	-0.51043
C	-4.51304	-0.82878	0.37567
C	-3.48155	-1.65389	0.83959
C	-2.16509	-1.43324	0.41741
H	-2.68994	1.25107	-1.62188
H	-5.03042	0.85879	-0.87130
H	-5.53706	-1.00042	0.70407
H	-3.70322	-2.46734	1.52886
H	-1.36273	-2.07506	0.77828

50

isomer-1 of **8c**$^+$ SCF done: -2990.503494

C	0.010600	-0.006704	-0.003776
C	-0.003318	-0.002128	1.424949
C	1.354033	-0.010085	1.878082
C	2.187576	-0.016875	0.736225
C	1.363270	-0.018286	-0.417933
Fe	0.952567	1.649670	0.733469
C	-0.055137	3.247683	-0.044541
C	-0.069452	3.278401	1.383857

C	1.286284	3.352470	1.835839
C	2.119349	3.364223	0.693673
C	1.296136	3.303417	-0.459665
P	-1.506744	3.152031	2.429264
P	-2.876684	1.588222	1.988337
P	-1.445137	0.092319	2.467494
C	-2.246918	-1.561696	2.577143
C	-1.156865	-2.580262	2.929093
C	-2.882498	-1.904039	1.226326
C	-3.307857	-1.511916	3.681655
C	-2.374512	4.774646	2.498071
C	-3.433084	4.709768	3.604068
C	-3.022746	5.057317	1.139484
C	-1.326507	5.844731	2.823898
H	1.680074	-0.000062	2.905893
H	3.265122	0.001230	0.743859
H	1.706847	-0.000434	-1.439269
H	-0.855158	0.028846	-0.645076
H	1.612037	3.381393	2.863382
H	3.196744	3.389792	0.701114
H	1.640578	3.273815	-1.480436
H	-0.918494	3.161161	-0.684225
H	-3.352408	-2.886289	1.301123
H	-3.654753	-1.187676	0.941600
H	-2.138851	-1.954059	0.429596
H	-3.751023	-2.504152	3.783882
H	-2.876933	-1.240655	4.647534
H	-4.107688	-0.808127	3.451125
H	-1.618072	-3.565141	3.021447
H	-0.389289	-2.641955	2.157014
H	-0.676199	-2.350780	3.882332
H	-3.531835	6.021424	1.190004
H	-2.281357	5.117316	0.341340
H	-3.765423	4.303577	0.873155
H	-3.915885	5.685599	3.681755
H	-4.203827	3.968816	3.391612
H	-2.992023	4.480258	4.576174
H	-1.827031	6.812242	2.891915
H	-0.837419	5.658636	3.782297
H	-0.561691	5.917997	2.050093
H	-0.913675	3.023423	3.700332
H	-0.858438	0.276358	3.734695

50

8c'$^{+}$ SCF done: -2990.498569

C	0.018141	-0.003114	-0.044319
C	-0.001507	-0.014143	1.388869
C	1.355477	-0.024292	1.850812
C	2.189815	-0.025142	0.711718
C	1.372185	-0.015228	-0.447496
Fe	0.957148	1.644051	0.721661
C	0.018739	3.301344	1.476930
C	1.408182	3.323248	1.816337
C	2.159892	3.323757	0.618310

C	1.252218	3.302514	-0.470821
C	-0.064240	3.288169	0.049312
P	-1.263433	3.240247	2.759636
C	-2.218931	4.842784	2.556555
C	-2.658755	5.174445	1.133062
P	-1.435265	0.073685	2.412921
C	-2.344733	-1.518902	2.477834
C	-1.351880	-2.568727	2.993822
P	-2.637690	1.770004	1.763685
C	-3.436351	4.761548	3.483511
C	-1.254608	5.924582	3.064768
C	-3.521926	-1.367122	3.447008
C	-2.835024	-1.883994	1.073754
H	1.805502	3.320633	2.818981
H	1.517838	3.273137	-1.515199
H	1.675749	-0.021423	2.880234
H	-0.970845	3.249379	-0.532614
H	3.267242	-0.010422	0.724647
H	3.235248	3.316472	0.546039
H	-0.841390	0.025297	-0.694457
H	-1.757187	6.893851	3.033893
H	-0.944766	5.741467	4.095373
H	-0.361511	5.994732	2.440859
H	-3.919108	5.739711	3.533677
H	-4.183804	4.053433	3.117893
H	-3.156911	4.476178	4.499783
H	-3.222109	6.110610	1.140547
H	-1.806080	5.311273	0.468659
H	-3.310598	4.407513	0.708318
H	-3.387057	-2.823534	1.131879
H	-3.508247	-1.129131	0.663428
H	-2.005464	-2.030478	0.381130
H	-1.859109	-3.534127	3.036459
H	-0.489621	-2.673533	2.333951
H	-0.998003	-2.336963	4.000359
H	-3.998616	-2.340768	3.571262
H	-3.202326	-1.029478	4.435309
H	-4.278413	-0.678669	3.068409
H	1.721582	0.008654	-1.466587
H	-3.519671	1.630115	2.863307
H	-0.942598	0.295742	3.707583

82

*t*Bu substituted counterpart of **3**$^{+}$[PPh_3] SCF done: -4025.622958

C	-3.376878	-1.460729	-1.387954
C	-3.082428	-1.181236	-0.057462
C	-3.924362	-1.657826	0.947753
C	-5.011659	-2.448572	0.622044
C	-5.279171	-2.755151	-0.704523
C	-4.467684	-2.251985	-1.707899
P	-1.753769	-0.024029	0.365378
C	-1.067896	-0.460427	1.971856
C	-1.121613	-1.763650	2.463951
C	-0.495804	-2.079773	3.658171

C	0.195800	-1.106966	4.363473
C	0.258299	0.189816	3.875778
C	-0.370147	0.511652	2.686635
P	-0.365132	0.095281	-1.424621
P	1.177910	1.656568	-1.654639
C	0.479622	3.387335	-1.522800
C	-0.605017	3.543882	-2.589827
C	2.464332	1.603746	-0.390883
C	3.838110	1.503943	-0.791892
C	4.644537	1.508850	0.369143
C	3.791414	1.613328	1.497382
C	2.455735	1.679193	1.035061
Fe	3.306799	-0.067825	0.397420
C	3.436150	-1.733720	1.611381
C	4.378733	-1.828369	0.555739
C	3.672799	-1.767710	-0.667978
C	2.274673	-1.635109	-0.378341
C	2.144297	-1.623679	1.043468
P	1.097964	-1.526375	-1.744432
C	0.284520	-3.215260	-1.764088
C	-0.444575	-3.621194	-0.488220
C	1.457253	-4.184354	-1.995245
C	-0.647138	-3.279791	-2.975869
C	1.674259	4.282373	-1.900414
C	-0.029576	3.806827	-0.146680
C	-2.669226	1.531890	0.502346
C	-2.938308	2.155062	1.717162
C	-3.701449	3.311536	1.745145
C	-4.208627	3.843215	0.570662
C	-3.975051	3.202456	-0.637216
C	-3.216619	2.047478	-0.671598
H	1.227232	-1.517281	1.591994
H	3.664052	-1.722537	2.665009
H	5.448516	-1.900216	0.665370
H	4.104166	-1.788839	-1.656050
H	1.583531	1.746267	1.656459
H	4.104430	1.618470	2.528921
H	5.718180	1.418028	0.393272
H	4.182241	1.414102	-1.809867
H	1.053871	-5.185451	-2.163590
H	2.046212	-3.910799	-2.872930
H	2.122851	-4.230979	-1.133134
H	-1.061299	-4.287162	-3.062647
H	-1.482997	-2.587809	-2.889926
H	-0.112656	-3.053808	-3.899726
H	-0.750225	-4.668868	-0.557836
H	0.197545	-3.520718	0.387880
H	-1.347698	-3.031791	-0.339447
H	-0.951315	4.580037	-2.605650
H	-0.223030	3.300636	-3.582602
H	-1.467759	2.910309	-2.393707
H	-0.353674	4.850436	-0.182703
H	-0.880989	3.211757	0.174740
H	0.756680	3.736068	0.605308

H	1.332706	5.319109	-1.944275
H	2.474784	4.225478	-1.161900
H	2.085007	4.023497	-2.877949
H	-1.636477	-2.539313	1.916360
H	-0.543053	-3.093541	4.032786
H	0.686296	-1.359399	5.294409
H	0.797756	0.952701	4.421372
H	-0.320592	1.526480	2.314517
H	-2.569339	1.746100	2.646524
H	-3.903222	3.793948	2.692313
H	-4.799968	4.749028	0.597540
H	-4.387037	3.600560	-1.554945
H	-3.056053	1.544372	-1.616753
H	-3.743748	-1.407950	1.984841
H	-5.656599	-2.820212	1.407227
H	-6.129589	-3.375591	-0.955154
H	-4.683064	-2.470929	-2.745327
H	-2.760902	-1.054893	-2.178489

48

*t*Bu substituted counterpart of **3**$^{+}$ SCF done: -2989.251876

C	-0.057654	-0.027092	-0.004900
C	-0.002547	0.006433	1.424235
C	1.379818	0.048098	1.794372
C	2.151003	0.024899	0.609439
C	1.266769	-0.021975	-0.498434
Fe	0.943524	-1.650551	0.747362
C	-0.271785	-3.292329	0.712714
C	0.458814	-3.192568	1.945731
C	1.858908	-3.248881	1.634439
C	1.975949	-3.380727	0.233837
C	0.673905	-3.408453	-0.328279
P	-0.238148	-2.917736	3.541321
C	-0.148693	-4.386754	4.654289
C	1.329424	-4.762803	4.826240
P	-1.505854	-0.006057	2.424750
C	-1.579552	1.675917	3.255850
C	-2.830147	1.703111	4.139860
P	-1.250228	-1.315320	4.141485
C	-0.908672	-5.523325	3.954335
C	-0.781837	-4.055592	6.004711
C	-1.745496	2.670615	2.097466
C	-0.339092	2.035242	4.071304
H	2.670229	-3.176395	2.340300
H	2.901999	-3.426671	-0.315455
H	0.441468	-3.480001	-1.378246
H	-1.344606	-3.268655	0.609323
H	1.765046	0.059520	2.801264
H	3.227834	0.014326	0.558479
H	1.554243	-0.071299	-1.535996
H	-0.958876	-0.080462	-0.594875
H	-0.822701	-6.422063	4.568569
H	-1.968307	-5.291095	3.841254
H	-0.490172	-5.750895	2.972825

H	-0.708528	-4.939781	6.640458
H	-0.265550	-3.238741	6.510855
H	-1.837516	-3.799153	5.911670
H	1.385373	-5.644245	5.467836
H	1.798711	-5.019410	3.876057
H	1.899926	-3.964264	5.302830
H	-2.967515	2.713156	4.531437
H	-3.728733	1.436375	3.580970
H	-2.746449	1.033927	4.999015
H	-0.463100	3.034346	4.495734
H	-0.181125	1.344452	4.903077
H	0.559864	2.051957	3.455274
H	-1.859564	3.675711	2.508798
H	-0.874143	2.678065	1.440542
H	-2.631735	2.453346	1.498233

49

isomer-1 of **9** SCF done: -2990.102092

C	0.000949	-0.004954	0.019765
C	0.015671	-0.003720	1.435388
C	1.370952	-0.003903	1.858111
C	2.192595	0.000577	0.703632
C	1.350275	-0.003373	-0.451749
Fe	0.998116	1.658215	0.704765
C	0.069924	3.362124	0.022973
C	0.084545	3.357604	1.438590
C	1.438685	3.301476	1.861243
C	2.259485	3.265535	0.706716
C	1.418065	3.306175	-0.448613
P	1.813772	3.249609	-2.224386
P	3.322029	1.613360	-2.189354
P	1.748057	0.040291	-2.227422
C	2.730141	-1.548008	-2.490490
C	3.234012	-1.517687	-3.937319
C	1.714737	-2.685872	-2.330407
C	3.905081	-1.782786	-1.543638
C	2.860043	4.796876	-2.484547
C	1.892040	5.975041	-2.322370
C	3.362341	4.748615	-3.931436
C	4.043550	4.981650	-1.537286
H	3.474997	1.611562	-3.599880
H	-0.873105	0.016524	-0.611480
H	-0.848647	0.018954	2.079183
H	1.715907	0.022615	2.879303
H	3.269272	0.043479	0.695329
H	-0.804261	3.377660	-0.608266
H	-0.779993	3.369128	2.082385
H	1.782237	3.258915	2.882366
H	3.333502	3.178586	0.698276
H	4.438690	-2.690405	-1.842206
H	4.617202	-0.955569	-1.562281
H	3.567813	-1.923499	-0.517036
H	3.662009	-2.489321	-4.199041
H	2.425875	-1.304571	-4.640500

H	4.014162	-0.766489	-4.076963
H	2.201818	-3.644549	-2.530873
H	1.312114	-2.720561	-1.315911
H	0.879702	-2.578762	-3.025816
H	4.613869	5.867216	-1.834208
H	3.712261	5.134178	-0.510425
H	4.721226	4.126021	-1.557448
H	3.829763	5.702396	-4.191378
H	4.111102	3.966373	-4.072469
H	2.546204	4.570044	-4.634997
H	2.417953	6.913349	-2.521092
H	1.053362	5.903459	-3.017960
H	1.491115	6.024325	-1.307805

49

isomer-2 of **9** SCF done: -2990.103707

C	0.014211	-0.003234	0.016369
C	0.007111	-0.004577	1.445299
C	1.373550	-0.004577	1.864771
C	2.201381	-0.000592	0.716320
C	1.360513	-0.004026	-0.427108
Fe	0.969990	1.657125	0.722524
C	-0.053142	3.260290	-0.020457
C	-0.060874	3.293619	1.408083
C	1.304232	3.359430	1.826731
C	2.131990	3.363634	0.678227
C	1.292160	3.306568	-0.464545
P	-1.425589	3.240425	2.611373
P	-2.714640	1.594798	1.928678
P	-1.359249	0.019429	2.647658
C	-2.310637	-1.561693	2.270823
C	-1.333068	-2.716490	2.515673
C	-2.881879	-1.669954	0.858315
C	-3.451384	-1.635583	3.291277
C	-2.441134	4.772157	2.199585
C	-3.584047	4.822256	3.219060
C	-3.016210	4.824789	0.785465
C	-1.511868	5.971399	2.417370
H	-2.432986	1.584949	0.539775
H	1.702847	0.017030	2.891424
H	3.279004	0.027110	0.710240
H	1.687377	0.022425	-1.454249
H	-0.854406	0.037106	-0.619984
H	1.633726	3.374611	2.853435
H	3.209841	3.380254	0.672208
H	1.620255	3.270417	-1.490998
H	-0.919100	3.169809	-0.655278
H	-3.442694	-2.604560	0.759655
H	-3.568362	-0.850863	0.633421
H	-2.094060	-1.681324	0.105531
H	-3.987802	-2.581474	3.174650
H	-3.074864	-1.583286	4.314607
H	-4.169081	-0.824717	3.151537
H	-1.857268	-3.670239	2.407376

H	-0.508874	-2.702463	1.800039
H	-0.911295	-2.676762	3.522368
H	-3.614981	5.733066	0.666131
H	-2.229456	4.851481	0.031958
H	-3.668403	3.973275	0.579760
H	-4.158846	5.742438	3.081489
H	-4.267825	3.979648	3.098329
H	-3.205791	4.808674	4.242997
H	-2.074799	6.900105	2.287884
H	-1.088937	5.971899	3.424361
H	-0.687711	5.974994	1.701564

49

isomer-3 of **9** SCF done: -2990.0782739

C	-1.85251	0.61390	-0.64931
C	-2.14258	-0.38508	-1.63172
C	-3.13521	-1.25450	-1.12013
C	-3.47613	-0.80968	0.18020
C	-2.69501	0.33849	0.46828
Fe	-1.47333	-1.27504	0.09676
C	0.07969	-1.36411	1.43428
C	0.55639	-1.65104	0.11687
C	-0.16057	-2.80435	-0.33103
C	-1.05786	-3.20973	0.68301
C	-0.90339	-2.32263	1.77939
P	1.85363	-0.91708	-0.92225
P	0.93695	0.89888	-1.84475
P	-0.73896	2.01835	-0.96902
C	-0.45035	2.93055	0.65085
C	-1.72999	3.74827	0.88641
C	-0.12052	2.11149	1.89071
C	0.70053	3.90370	0.37089
C	3.30671	-0.50299	0.20039
C	3.10162	0.54617	1.28329
C	3.72019	-1.83323	0.84210
C	4.41991	-0.03044	-0.74367
H	-1.66562	-0.47043	-2.59520
H	-4.18537	-1.27159	0.84779
H	-0.03822	-3.26581	-1.29811
H	-2.73331	0.88823	1.39233
H	-1.75314	-4.03149	0.62412
H	-3.53350	-2.12130	-1.62215
H	0.39514	-0.54980	2.06033
H	-1.59865	4.37955	1.77008
H	-1.95032	4.39633	0.03659
H	-2.60055	3.11384	1.05715
H	0.81322	4.58883	1.21535
H	1.64842	3.37955	0.23821
H	0.51468	4.50012	-0.52499
H	0.04834	2.78313	2.73836
H	-0.91801	1.42453	2.16382
H	0.78566	1.53452	1.73946
H	4.05078	0.74021	1.79236

H	2.75310	1.49011	0.86309
H	2.39280	0.21361	2.04026
H	4.64964	-1.69491	1.40201
H	2.95919	-2.19757	1.53422
H	3.89250	-2.60493	0.08905
H	5.34408	0.11471	-0.17747
H	4.61377	-0.75941	-1.53220
H	4.17024	0.92230	-1.21630
H	-1.45826	-2.35161	2.70345
H	1.97020	1.84913	-1.68197

97

Fc'(TipP)$_2$ SCF done: -3503.396618

C	1.489339	1.271962	1.541533
C	0.440058	1.660260	0.682089
C	0.649002	2.752898	-0.182359
C	1.823136	3.483828	-0.091733
C	2.833411	3.155456	0.797726
C	2.650404	2.036293	1.587493
P	-1.108365	0.664792	0.886339
C	-2.679380	1.510114	0.437050
C	-3.314340	1.839697	-0.810159
C	-4.706705	1.972234	-0.592151
C	-4.965820	1.724573	0.779911
C	-3.729379	1.453123	1.411494
Fe	-4.021326	0.084813	-0.090754
C	-3.478873	-1.710591	0.669055
C	-2.740457	-1.422953	-0.530931
C	-3.716074	-1.299272	-1.574209
C	-5.007454	-1.489849	-1.029079
C	-4.858769	-1.753660	0.356281
P	-1.091650	-0.680093	-0.870501
C	0.373906	-1.769477	-0.562056
C	1.504686	-1.445685	-1.346367
C	2.615192	-2.277023	-1.316392
C	2.671912	-3.410448	-0.522417
C	1.585654	-3.676730	0.290211
C	0.453600	-2.871526	0.307024
C	1.588842	-0.204922	-2.225399
C	1.416126	-0.565080	-3.700090
C	-0.651313	-3.216550	1.280521
C	-0.144711	-3.560960	2.681715
C	3.906586	-4.282610	-0.482116
C	4.294808	-4.801495	-1.866430
C	-0.364755	3.161454	-1.228087
C	0.254343	3.474047	-2.590967
C	4.121010	3.946513	0.851432
C	4.918215	3.774573	-0.443798
C	1.438317	0.033949	2.426709
C	2.685490	-0.836698	2.267594
C	-1.215945	4.333552	-0.739029
C	1.195549	0.412772	3.886826
C	3.875981	5.424102	1.157347
C	-1.536710	-4.336960	0.734433

C	5.071057	-3.538965	0.176806
C	2.875593	0.584944	-1.983041
H	-3.489101	-1.036779	-2.595285
H	-5.656841	-1.933438	1.058580
H	-5.932965	1.730927	1.255811
H	-5.442095	2.200067	-1.346981
H	-2.002906	4.570128	-1.458769
H	2.861498	-1.078421	1.218610
H	-1.005562	2.300397	-1.383396
H	1.630339	-4.535705	0.949715
H	0.767922	0.458859	-1.959780
H	3.471591	-2.021079	-1.928264
H	-1.248280	-2.318087	1.392673
H	0.595764	-0.578521	2.110668
H	3.668304	-5.148217	0.142998
H	-3.074947	-1.820774	1.660430
H	3.447798	1.740501	2.259240
H	-5.938321	-1.433997	-1.569675
H	0.852942	4.386744	-2.576559
H	-0.537884	3.619772	-3.328330
H	0.888961	2.656641	-2.935599
H	2.205608	-1.244055	-4.031574
H	1.457670	0.332342	-4.321922
H	0.455137	-1.054324	-3.868779
H	0.402378	-4.505175	2.705481
H	-0.992112	-3.661006	3.363256
H	0.509465	-2.779356	3.070191
H	-3.589697	1.181023	2.445654
H	4.719472	3.531978	1.667959
H	-1.929385	-4.088093	-0.251384
H	-2.383845	-4.521909	1.398860
H	-0.962941	-5.263155	0.648172
H	3.466469	-5.337026	-2.332525
H	5.146760	-5.480737	-1.795280
H	4.579868	-3.982061	-2.529977
H	1.961125	4.329313	-0.754837
H	-0.593776	5.222778	-0.609628
H	-1.689478	4.108788	0.216576
H	-2.837608	1.919600	-1.771643
H	3.000908	0.810172	-0.923208
H	2.840040	1.533233	-2.522586
H	3.761132	0.047920	-2.329116
H	5.110711	2.720183	-0.648324
H	5.876190	4.295001	-0.380447
H	4.368807	4.183841	-1.294955
H	5.356180	-2.665155	-0.413974
H	5.945605	-4.187320	0.263076
H	4.798781	-3.191281	1.174696
H	2.556132	-1.775907	2.809014
H	3.580148	-0.352980	2.664762
H	2.003233	1.042885	4.267162
H	1.141320	-0.481988	4.511475
H	0.258232	0.961840	3.993080
H	3.305990	5.902969	0.358006

H	4.823777	5.957651	1.253790
H	3.316997	5.546038	2.086243

132

6^{+} SCF done: -4880.934862

C	-0.903868	-3.442281	-1.968696
C	-1.358465	-2.753978	-0.846408
C	-2.727779	-2.637507	-0.619907
C	-3.629645	-3.184379	-1.512988
C	-3.174413	-3.854735	-2.637365
C	-1.812944	-3.988388	-2.859462
P	-0.213187	-1.955470	0.298595
C	1.406034	-2.723106	0.115378
C	2.139006	-2.518341	-1.054364
C	3.355814	-3.147272	-1.227413
C	3.855915	-3.975306	-0.234623
C	3.139150	-4.169665	0.934669
C	1.916088	-3.544449	1.115820
P	0.073749	0.119061	-0.375238
P	1.772192	0.616009	1.023763
C	3.281837	0.342776	0.002303
C	4.327873	-0.326699	0.689615
C	5.465740	-0.691260	-0.009581
C	5.616833	-0.445038	-1.366235
C	4.602547	0.233873	-2.012423
C	3.450389	0.661173	-1.359209
C	4.276531	-0.594808	2.188573
C	4.550436	0.698747	2.965455
C	6.869188	-0.867796	-2.101294
C	8.091660	-0.106062	-1.584598
C	2.488527	1.508253	-2.177496
C	1.822620	0.739577	-3.321167
C	1.668915	2.405306	1.297657
C	0.995411	2.975514	2.425830
C	1.249881	4.365796	2.444095
C	2.092401	4.671933	1.344908
C	2.353796	3.473913	0.642751
Fe	0.326642	3.816564	0.683102
C	-0.564087	3.372548	-1.104893
C	-1.319168	2.850995	-0.007577
C	-1.703530	3.970644	0.798195
C	-1.190567	5.147403	0.206183
C	-0.482430	4.777439	-0.964180
P	-1.612738	1.184547	0.639535
C	-3.151034	0.584598	-0.172931
C	-3.430333	0.629892	-1.551364
C	-4.702715	0.288490	-1.992505
C	-5.710611	-0.101268	-1.130270
C	-5.407104	-0.183614	0.220626
C	-4.156482	0.142985	0.721705
C	-2.423741	1.026852	-2.612599
C	-2.176775	-0.107733	-3.608022
C	-3.951190	0.038603	2.227441
C	-4.566244	-1.219600	2.839463

C	-7.100846	-0.409020	-1.640645
C	-7.492713	-1.866029	-1.392842
C	5.223561	-1.690193	2.671752
C	7.093488	-2.378735	-2.026176
C	3.187994	2.745494	-2.758308
C	-4.473166	1.297467	2.921612
C	-8.130795	0.547837	-1.036201
C	-2.834344	2.318001	-3.320964
C	-0.815377	-2.280523	1.965810
C	-1.453537	-3.493093	2.231004
C	-1.861338	-3.790971	3.518780
C	-1.639958	-2.884364	4.544946
C	-1.003087	-1.681521	4.284889
C	-0.584440	-1.378132	3.000215
H	4.723837	0.465514	-3.064652
H	-2.881857	-0.016430	2.421522
H	3.262492	-0.919592	2.430413
H	6.265068	-1.193777	0.516925
H	1.711541	1.870458	-1.507660
H	-1.473092	1.212487	-2.125463
H	6.732092	-0.603244	-3.153465
H	2.970065	3.381293	-0.231914
H	-4.919525	0.337691	-3.053340
H	0.851534	5.071998	3.154314
H	-5.657030	-1.198255	2.832825
H	-4.251983	-1.309054	3.880423
H	-4.241505	-2.121564	2.317044
H	-1.293496	6.147106	0.595443
H	5.552630	1.069078	2.738207
H	4.490726	0.517932	4.040182
H	3.842632	1.490957	2.718259
H	2.569009	0.357114	-4.019839
H	1.158070	1.401894	-3.881371
H	1.222993	-0.095922	-2.963873
H	-0.119632	2.804812	-1.904888
H	-7.090595	-0.246790	-2.721788
H	0.374722	2.433493	3.121844
H	3.824288	3.246088	-2.029030
H	2.445490	3.461976	-3.114969
H	3.820603	2.479266	-3.606413
H	0.045249	5.446336	-1.624559
H	7.941636	0.972042	-1.653366
H	8.977907	-0.366406	-2.165322
H	8.292893	-0.351522	-0.539563
H	2.456210	5.650600	1.077318
H	-6.179688	-0.497873	0.910763
H	-5.545065	1.417835	2.750444
H	-3.973446	2.190457	2.541469
H	-4.304153	1.242965	3.998690
H	-2.252071	3.913837	1.724978
H	5.122738	-2.602849	2.084450
H	4.997758	-1.929957	3.711746
H	6.268030	-1.373547	2.638078
H	-6.814651	-2.551515	-1.904031

H	-8.500471	-2.057901	-1.764114
H	-7.479787	-2.104697	-0.327078
H	7.232603	-2.706801	-0.993683
H	7.986796	-2.659493	-2.585879
H	6.249076	-2.928329	-2.445578
H	-1.866691	-1.019471	-3.095187
H	-1.388666	0.173851	-4.309110
H	-3.070496	-0.339594	-4.189686
H	-3.759344	2.185183	-3.885011
H	-2.058395	2.626945	-4.024523
H	-2.986173	3.126561	-2.605225
H	-8.214438	0.405412	0.043389
H	-9.115421	0.372194	-1.471974
H	-7.856554	1.587641	-1.217954
H	1.366294	-3.698303	2.034508
H	3.532640	-4.807363	1.715032
H	4.814493	-4.459438	-0.369581
H	3.920588	-2.972325	-2.132306
H	1.771500	-1.857029	-1.826979
H	-1.634051	-4.203793	1.435241
H	-2.356519	-4.731642	3.719490
H	-1.965206	-3.117880	5.550462
H	-0.827296	-0.974203	5.084472
H	-0.074274	-0.445712	2.806589
H	0.153499	-3.569278	-2.150683
H	-1.452547	-4.523417	-3.727926
H	-3.880833	-4.280826	-3.337798
H	-4.688528	-3.079545	-1.321822
H	-3.100647	-2.120026	0.250814

38

1,6-Diphosphatricyclo[4.4.4.0]tetradecanediium HF=-1153.9761582

C	-0.094190	1.739127	-1.563622
P	0.000399	0.000297	-1.089343
C	-1.457796	-0.950890	-1.565006
C	-1.655671	-2.238246	-0.746740
C	-1.913265	-2.023447	0.744583
C	-0.681183	-1.603123	1.563776
P	-0.000297	-0.000438	1.089305
C	1.727810	0.210918	1.565089
C	2.708334	-0.645562	0.746070
C	2.766767	-0.314158	-0.744968
C	1.553773	-0.786243	-1.564464
C	-1.110379	2.553629	-0.745226
C	-0.796099	2.668102	0.746268
C	-1.048247	1.390133	1.564283
H	-0.340173	1.756939	-2.629628
H	0.911730	2.156530	-1.463290
H	1.787753	-0.029685	2.630784
H	1.962366	1.274577	1.466480
H	-0.871511	1.561887	2.630333
H	-2.086456	1.061197	1.463972
H	-0.919455	-1.535687	2.629545

H	0.122722	-2.337987	1.464466
H	1.692410	-0.580420	-2.630113
H	1.412585	-1.866267	-1.465828
H	-1.349290	-1.172799	-2.630923
H	-2.322288	-0.288393	-1.465431
H	3.696058	-0.479865	1.176407
H	2.498423	-1.708930	0.889325
H	3.639451	-0.805969	-1.174828
H	2.931177	0.757188	-0.888303
H	-2.518089	-2.747473	-1.177041
H	-0.810172	-2.916318	-0.890779
H	-2.263428	-2.962164	1.174051
H	-2.729328	-1.310257	0.888683
H	-1.119972	3.555443	-1.174788
H	-2.120302	2.160589	-0.889951
H	-1.434311	3.440201	1.176149
H	0.229453	3.018337	0.891015

39
1,6-Diphosphatricyclo[4.4.4.0]tetradecanediium+F HF=-1254.2091912

C	1.221742	-0.357549	-1.790425
P	1.294020	-0.009515	0.010704
F	2.940531	-0.021892	0.024065
C	0.458956	-1.607923	-2.259375
C	-1.059251	-1.472402	-2.352513
C	-1.768275	-1.364932	-1.001251
P	-1.117494	0.008309	-0.009255
C	-1.749241	1.556534	-0.714443
C	-1.046425	2.777557	-0.118319
C	0.474492	2.758865	-0.254096
C	1.217798	1.724839	0.608146
C	-1.781826	-0.152544	1.671834
C	-1.097968	-1.282133	2.444187
C	0.422151	-1.160935	2.524826
C	1.189700	-1.393424	1.212615
H	-1.307956	-0.608853	-2.975887
H	2.255245	2.029754	0.730884
H	0.779528	1.695022	1.610132
H	-2.862932	-0.301439	1.618959
H	-1.614096	0.801947	2.177430
H	-2.830555	1.605101	-0.565563
H	-1.575857	1.513969	-1.792831
H	-2.846792	-1.240691	-1.124232
H	-1.614924	-2.280360	-0.423925
H	2.222145	-1.655961	1.435024
H	0.758405	-2.244780	0.677735
H	2.259974	-0.421670	-2.110250
H	0.801467	0.532717	-2.267616
H	-1.503726	-1.292643	3.456629
H	-1.361303	-2.248861	2.005693
H	0.781022	-1.915292	3.226079
H	0.699094	-0.199403	2.965570
H	0.835798	-1.839446	-3.256355

H	0.721572	-2.471136	-1.641806
H	-1.458279	-2.342940	-2.874746
H	0.849422	3.736650	0.050810
H	0.756171	2.654560	-1.305432
H	-1.432774	3.666756	-0.618169
H	-1.314434	2.886399	0.936429

32
Ligand-Stabilized Trimethylphosphane Dication HF= -842.8605961

C	1.253930	-1.197588	0.000013
C	-0.089896	-1.147875	-0.000011
N	-0.790802	0.040727	-0.000031
C	-0.065897	1.212316	0.000039
C	1.280158	1.227902	0.000064
C	2.034429	0.007748	0.000062
P	-2.509070	-0.005300	-0.000008
C	-3.033562	-0.862540	-1.470793
N	3.346045	-0.007467	0.000008
C	4.084744	-1.275792	0.000030
C	-3.120789	1.665995	-0.000599
C	-3.033579	-0.861488	1.471376
C	4.112562	1.244179	-0.000096
H	-2.665024	-1.887632	1.481640
H	-2.677811	-0.333805	2.357319
H	-4.126064	-0.886423	1.482099
H	-0.625329	2.135198	0.000057
H	1.769259	2.189409	0.000086
H	1.722109	-2.169549	-0.000023
H	-0.673580	-2.058608	-0.000033
H	-2.677591	-0.335633	-2.357113
H	-2.665258	-1.888779	-1.480201
H	-4.126053	-0.887238	-1.481610
H	-2.814922	2.203190	-0.898842
H	-4.211919	1.593801	-0.000789
H	-2.815274	2.203678	0.897473
H	5.147811	-1.063227	0.000192
H	3.851275	-1.855635	0.893444
H	3.851525	-1.855521	-0.893526
H	5.170723	1.008416	-0.000466
H	3.891155	1.829043	-0.893328
H	3.891707	1.828856	0.893399

32
Ligand-Stabilized Trimethylphosphane Dication HF= -943.096735

C	-0.005896	-0.024441	0.025807
N	-0.018288	-0.033256	1.369723
C	1.183139	-0.024150	1.972235
C	2.376163	-0.045292	1.307991
C	2.399380	-0.069587	-0.106965
C	1.129954	-0.045627	-0.732245
N	3.540820	-0.104899	-0.804188
C	4.821541	-0.115085	-0.114935
C	3.512142	-0.116509	-2.258301
P	-1.821880	0.280747	2.479491

F	-3.169832	0.638201	3.313861
C	-2.712990	-0.677131	1.237392
C	-1.398916	2.025884	2.237907
C	-1.127796	-0.708853	3.818944
H	-3.102711	-0.014095	0.464545
H	-2.104784	-1.456493	0.789882
H	-3.564515	-1.121245	1.749141
H	1.186398	0.005480	3.053284
H	3.285555	-0.041303	1.887993
H	1.029092	-0.041846	-1.806130
H	-0.965982	0.004892	-0.470835
H	-0.424718	2.231990	2.678500
H	-1.336841	2.244884	1.173173
H	-2.157238	2.648916	2.703474
H	-0.624619	-0.061112	4.537351
H	-1.974232	-1.166622	4.327295
H	-0.448139	-1.478535	3.468324
H	4.529375	-0.166023	-2.633495
H	2.970372	-0.986702	-2.635308
H	3.048673	0.790350	-2.653786
H	5.619756	-0.162687	-0.848809
H	4.960460	0.791559	0.478594
H	4.911119	-0.985629	0.538548

49
threecoordinate phosphorus dications isoelectronic to silylium cation scf done: -1419.768829

N	0.000664	0.001627	0.000190
C	-0.000139	-0.000456	1.343437
N	1.277977	-0.002098	1.748741
C	2.100741	0.017789	0.642195
C	1.307431	0.017833	-0.444865
N	-1.055559	-0.096611	2.174767
P	-2.284132	0.817741	2.376060
N	-2.482668	2.054470	1.472313
C	-3.430540	3.011260	1.465407
N	-3.173182	4.324269	1.553155
C	-4.354624	5.023739	1.424435
C	-5.335448	4.120336	1.242951
N	-4.751232	2.869576	1.265405
C	-1.859903	4.908966	1.770748
C	-5.444707	1.609902	1.058428
C	1.718442	-0.003447	3.134354
C	-1.178910	-0.051294	-0.846111
N	-3.333416	0.474385	3.456315
C	-3.376012	-0.507665	4.377169
N	-3.405601	-1.835128	4.174095
C	-3.547219	-2.469456	5.392197
C	-3.610985	-1.512971	6.336688
N	-3.511933	-0.296772	5.694319
C	-3.346046	-2.494161	2.880809
C	-3.529850	1.007085	6.337413
H	1.551942	0.019145	-1.492208
H	3.172806	0.023567	0.729067
H	-0.871625	-0.337703	-1.848088

H	-1.867425	-0.804054	-0.464457
H	-1.669132	0.920149	-0.875765
H	2.659582	-0.543230	3.201704
H	1.858630	1.016738	3.489655
H	0.970320	-0.505783	3.741975
H	-6.392098	4.252301	1.091961
H	-4.388555	6.097930	1.467416
H	-6.423727	1.820725	0.637179
H	-4.885849	0.998033	0.351546
H	-5.557447	1.078944	2.001961
H	-1.816872	5.870381	1.265283
H	-1.678262	5.049921	2.835550
H	-1.105558	4.247634	1.352640
H	-3.597959	-3.540684	5.473536
H	-3.723708	-1.586357	7.403832
H	-3.685835	-3.519169	3.000385
H	-4.009877	-1.986393	2.182322
H	-2.327242	-2.487254	2.497557
H	-4.169759	0.955739	7.214632
H	-2.523645	1.296376	6.638285
H	-3.930802	1.738705	5.640850

50

threecoordinate phosphorus dications isoelectronic to silylium cation+F scf done: -1520.0063

N	3.260399	-0.841168	-1.028078
C	2.359564	-0.930172	-0.016345
N	2.577945	-2.132381	0.574259
C	3.609297	-2.785131	-0.076074
C	4.035533	-1.983464	-1.065236
N	1.524773	0.031160	0.299528
P	-0.000008	-0.000831	0.731604
F	0.001610	-0.002894	2.320638
C	1.903074	-2.614083	1.763875
C	3.388581	0.296836	-1.910412
N	-0.790267	1.304959	0.304104
C	-0.374010	2.507447	-0.014241
N	-0.901292	3.242438	-1.026465
C	-0.297856	4.484034	-1.065703
C	0.610498	4.515565	-0.077443
N	0.559636	3.296836	0.574296
C	-1.951904	2.784281	-1.907592
C	1.315532	2.952741	1.762888
N	-0.736024	-1.336985	0.300316
C	-1.986356	-1.577481	-0.015227
N	-3.136136	-1.163468	0.575153
C	-4.217924	-1.728076	-0.075595
C	-3.738005	-2.498925	-1.064580
N	-2.361067	-2.401064	-1.027318
C	-3.215248	-0.336480	1.763607
C	-1.440822	-3.082374	-1.909971
H	-5.229398	-1.530721	0.230335
H	-4.247606	-3.105982	-1.790878
H	-4.223435	-0.408130	2.163802

H	-2.510498	-0.699967	2.509743
H	-2.979925	0.699860	1.528572
H	-1.716305	-2.897758	-2.947470
H	-0.443931	-2.692550	-1.721273
H	-1.450485	-4.155344	-1.717528
H	1.286939	5.293485	0.227329
H	-0.569766	5.228218	-1.792333
H	1.758825	3.861753	2.161496
H	0.648177	2.526092	2.509976
H	2.094260	2.229367	1.528386
H	-1.652720	2.922636	-2.945751
H	-2.116485	1.727832	-1.711871
H	-2.874491	3.333694	-1.719608
H	3.946009	-3.758967	0.230324
H	4.816498	-2.120002	-1.791289
H	2.462637	-3.459577	2.155905
H	1.878255	-1.826023	2.514873
H	0.883417	-2.916397	1.532561
H	3.368470	-0.034024	-2.947957
H	2.550604	0.963128	-1.722564
H	4.321083	0.827288	-1.716840

XYZ coordinates of the investigated systems and their total energies, which were obtained after the geometry optimization at ωB97X-D/def2-SVP

103
Tip_triphosphoniumcation+alcl4 scf done: -5925.459141

C	-1.422801	-1.802332	2.911846
C	-1.532623	-0.690045	2.000660
C	-1.684852	0.510213	2.774515
C	-1.657459	0.136487	4.142159
C	-1.500713	-1.277691	4.227977
P	-1.478663	-0.781227	0.222627
P	0.152841	-1.243157	-0.863767
P	1.786248	-1.603531	0.469255
C	1.735470	-0.727996	2.051593
C	1.591056	0.663681	2.377331
C	1.613678	0.781765	3.790359
C	1.775870	-0.516515	4.354905
C	1.854492	-1.451847	3.289682
Fe	0.077532	-0.454292	3.186142
C	-3.050355	-1.191237	-0.567803
C	-3.505831	-2.529081	-0.588735
C	-4.724732	-2.788312	-1.211094
C	-5.490374	-1.777414	-1.798934
C	-5.014081	-0.468225	-1.749478
C	-3.801423	-0.143256	-1.139271
C	-2.738592	-3.684156	0.035268
C	-3.540342	-4.356382	1.153729
C	-3.294371	1.285143	-1.143622
C	-4.381773	2.328993	-0.890214
C	-6.813460	-2.097306	-2.468946

C	-7.830189	-2.647338	-1.462742
C	3.281418	-1.138591	-0.487237
C	3.476958	0.129441	-1.080205
C	4.642687	0.339002	-1.825376
C	5.614730	-0.640429	-1.988401
C	5.407975	-1.879226	-1.376678
C	4.264488	-2.157249	-0.632792
C	2.516717	1.299873	-0.954696
C	3.182469	2.480865	-0.238690
C	6.867412	-0.370570	-2.800389
C	6.961147	-1.301601	-4.013807
C	4.152141	-3.509669	0.063589
C	4.789185	-4.666268	-0.708900
C	1.932098	1.718324	-2.307714
C	8.126319	-0.450147	-1.930208
C	4.726948	-3.414098	1.483591
C	-2.519656	1.573791	-2.433698
C	-6.626050	-3.045352	-3.658109
C	-2.273282	-4.683265	-1.028055
Cl	-0.446428	2.680163	0.398720
Al	-0.608837	4.809869	-0.172672
Cl	-0.496335	4.880329	-2.321340
Cl	-2.516071	5.478059	0.551027
Cl	1.050569	5.799723	0.753075
H	4.796192	1.314642	-2.293601
H	-1.826921	-3.275810	0.496668
H	3.080830	-3.745626	0.158101
H	6.169456	-2.653439	-1.485689
H	1.665134	0.994072	-0.333114
H	-2.570072	1.384697	-0.315726
H	6.788620	0.662165	-3.178056
H	1.427560	1.485439	1.683704
H	-5.607379	0.329494	-2.201802
H	1.810057	-0.754810	5.416142
H	-3.127923	-5.152210	-1.539870
H	-1.674536	-5.485294	-0.570178
H	-1.656394	-4.186490	-1.792099
H	-1.424817	-1.857881	5.145507
H	5.801807	-3.175926	1.449670
H	4.603954	-4.366638	2.022291
H	4.234540	-2.622033	2.068069
H	2.712892	2.092686	-2.987937
H	1.193994	2.521653	-2.169818
H	1.432341	0.872611	-2.806840
H	-1.747377	1.519689	2.369666
H	-7.212573	-1.147161	-2.859987
H	1.963129	-2.531292	3.383736
H	3.634545	2.165232	0.714035
H	2.445178	3.268389	-0.025949
H	3.977690	2.928071	-0.855000
H	-1.717697	0.824776	4.982693
H	8.061409	0.235725	-1.072731
H	9.019702	-0.184119	-2.515573
H	8.276669	-1.468307	-1.537084

H	1.491773	1.711904	4.342111
H	-5.092509	-3.817512	-1.237024
H	-4.451417	-4.837585	0.766017
H	-3.845746	-3.624777	1.916837
H	-2.937268	-5.136217	1.644124
H	-1.293396	-2.848325	2.641577
H	4.441351	-4.695968	-1.752053
H	4.525079	-5.622742	-0.233346
H	5.888536	-4.606719	-0.716980
H	-5.908466	-2.636752	-4.384696
H	-7.583130	-3.208865	-4.176243
H	-6.252670	-4.029060	-3.331650
H	7.065663	-2.353584	-3.703713
H	7.837023	-1.047775	-4.630219
H	6.063685	-1.224396	-4.645120
H	-1.679345	0.873378	-2.561969
H	-2.106024	2.592275	-2.416518
H	-3.177319	1.475898	-3.312357
H	-5.055309	2.438449	-1.754934
H	-3.917979	3.308746	-0.705105
H	-4.989977	2.071079	-0.010135
H	-7.500635	-3.613634	-1.048718
H	-8.805339	-2.808057	-1.947004
H	-7.975176	-1.952578	-0.622433

106

[IV]2[AlCl4]2 scf done: -10142.2377503

C	-4.409918	-2.006461	2.880305
C	-4.253970	-3.403296	2.650396
C	-3.622549	-3.573965	1.387885
C	-3.378997	-2.271577	0.831698
C	-3.890004	-1.307823	1.760724
Fe	-2.411883	-2.477643	2.586657
C	-1.139757	-1.513134	3.861260
C	-0.634767	-1.509993	2.504844
C	-0.485615	-2.888025	2.110882
C	-0.872149	-3.703360	3.203123
C	-1.271107	-2.859462	4.276850
P	-0.351877	-0.063136	1.474442
C	-0.568857	1.489967	2.508836
C	-2.052548	1.624649	2.883725
P	-2.368728	-2.080153	-0.665036
C	-3.639163	-1.855920	-2.044019
C	-4.071585	-3.299451	-2.358737
P	-1.631842	0.062075	-0.385817
P	0.352193	-0.064054	-1.474616
P	1.632159	0.062240	0.385546
P	2.369161	-2.079779	0.666052
C	3.639663	-1.854428	2.044776
C	4.072211	-3.297679	2.360604
C	0.634854	-1.511701	-2.503966
C	1.139496	-1.515933	-3.860504
C	1.270791	-2.862592	-4.275031
C	0.872115	-3.705628	-3.200523

C	0.485815	-2.889416	-2.108859
Fe	2.411965	-2.479376	-2.585370
C	4.409960	-2.008373	-2.879586
C	4.254032	-3.405061	-2.648763
C	3.622769	-3.574916	-1.386065
C	3.379307	-2.272172	-0.830660
C	3.890206	-1.309019	-1.760374
C	0.569166	1.488224	-2.510262
C	2.052679	1.622283	-2.886073
C	-0.366150	1.413324	-3.729228
C	0.157431	2.704829	-1.680701
C	2.921749	-1.253064	3.257344
C	4.852245	-1.018118	1.644690
C	-2.921250	-1.255439	-3.257021
C	-4.851807	-1.019360	-1.644655
C	0.365855	1.415923	3.728308
C	-0.156384	2.705717	1.678397
Cl	-2.752011	4.429217	0.043675
Al	-4.419120	3.349531	-0.792644
Cl	-6.076828	4.602112	-1.251456
Cl	-5.013828	1.823426	0.655951
Cl	-3.724277	2.323738	-2.590745
Cl	2.751362	4.430886	-0.044046
Al	4.418511	3.350654	0.791420
Cl	3.724458	2.325247	2.590014
Cl	5.011661	1.824067	-0.657367
Cl	6.077108	4.602500	1.249001
H	-1.388388	-0.651235	4.470259
H	-1.643524	-3.184156	5.246075
H	-0.889066	-4.791101	3.201821
H	-0.149634	-3.242060	1.142767
H	-3.894908	-0.226782	1.623705
H	-4.845598	-1.546434	3.765116
H	-4.545551	-4.201697	3.329957
H	-3.327119	-4.522666	0.941546
H	-2.454727	0.770556	3.442933
H	-2.156414	2.521016	3.513173
H	-2.684848	1.796161	1.999763
H	0.136542	2.285136	4.363016
H	0.261810	0.515178	4.342695
H	1.415966	1.522523	3.424579
H	0.904876	2.689191	1.393194
H	-0.799227	2.850813	0.803709
H	-0.296147	3.598113	2.306299
H	-4.810765	-3.279220	-3.175380
H	-3.224948	-3.924894	-2.682943
H	-4.554806	-3.781000	-1.493791
H	-2.010164	-1.816267	-3.522971
H	-3.594688	-1.274066	-4.128225
H	-2.668187	-0.198948	-3.087163
H	-5.538112	-0.939718	-2.503012
H	-5.403816	-1.480261	-0.812536
H	-4.581161	0.003022	-1.352845
H	1.387864	-0.654525	-4.470301

H	0.150091	-3.242683	-1.140378
H	2.454340	0.767683	-3.444875
H	2.156345	2.518127	-3.516302
H	2.685612	1.794290	-2.002660
H	-0.136785	2.281786	-4.364944
H	-0.262748	0.511898	-4.342735
H	-1.416074	1.520649	-3.425107
H	-0.903625	2.688712	-1.394677
H	0.800995	2.850830	-0.806673
H	0.296755	3.596546	-2.309669
H	3.895146	-0.227897	-1.624014
H	3.327389	-4.523332	-0.939088
H	4.811506	-3.276751	3.177126
H	3.225653	-3.922904	2.685436
H	4.555331	-3.779903	1.495977
H	2.010731	-1.813778	3.523766
H	3.595224	-1.270942	4.128534
H	2.668573	-0.196727	3.086685
H	5.538453	-0.937508	2.503032
H	5.404380	-1.479853	0.813112
H	4.581561	0.003930	1.351744
H	1.642977	-3.188063	-5.244084
H	0.889071	-4.793367	-3.198337
H	4.845538	-1.548913	-3.764742
H	4.545522	-4.203899	-3.327850

97
Fc'(TipP)2 scf done: -3501.371906

C	1.501538	1.227537	1.545986
C	0.441557	1.651062	0.703940
C	0.655344	2.760364	-0.149360
C	1.850502	3.473983	-0.063964
C	2.874476	3.111460	0.808915
C	2.682977	1.974788	1.586783
P	-1.120267	0.657926	0.904210
C	-2.694655	1.510262	0.438475
C	-3.310254	1.823845	-0.828176
C	-4.711751	1.961705	-0.632947
C	-4.993310	1.732817	0.744098
C	-3.762245	1.466603	1.401875
Fe	-4.034688	0.088648	-0.089544
C	-3.476630	-1.687906	0.691530
C	-2.758498	-1.417441	-0.530541
C	-3.754492	-1.305875	-1.562757
C	-5.041468	-1.490346	-0.989178
C	-4.867893	-1.735198	0.402822
P	-1.104487	-0.670181	-0.889106
C	0.373253	-1.760952	-0.586885
C	1.514717	-1.405751	-1.354646
C	2.644884	-2.224253	-1.318474
C	2.708194	-3.375349	-0.536205
C	1.607587	-3.671812	0.260930
C	0.456429	-2.880031	0.272364

C	1.580109	-0.156555	-2.231579
C	1.346585	-0.510170	-3.702517
C	-0.664845	-3.255954	1.222422
C	-0.179339	-3.654997	2.618891
C	3.959456	-4.232226	-0.493679
C	4.377281	-4.719113	-1.884084
C	-0.374799	3.204488	-1.170673
C	0.225507	3.573901	-2.530091
C	4.180297	3.882337	0.854928
C	4.971607	3.691836	-0.444551
C	1.429505	-0.019601	2.425006
C	2.683988	-0.889622	2.306537
C	-1.240045	4.343409	-0.622445
C	1.122306	0.352868	3.877618
C	3.965558	5.365597	1.169447
C	-1.561010	-4.341573	0.618675
C	5.102405	-3.490392	0.209623
C	2.876035	0.632976	-2.027930
H	-3.544696	-1.058695	-2.602427
H	-5.660870	-1.908021	1.128123
H	-5.976661	1.750015	1.209938
H	-5.441745	2.184100	-1.409086
H	-2.051452	4.595237	-1.323378
H	2.909561	-1.113922	1.253562
H	-1.012293	2.334512	-1.351858
H	1.655690	-4.547733	0.912833
H	0.765944	0.515024	-1.929603
H	3.512742	-1.944834	-1.920112
H	-1.257718	-2.347307	1.360347
H	0.596055	-0.639155	2.069148
H	3.718890	-5.122364	0.111779
H	-3.049209	-1.785739	1.686199
H	3.491719	1.652516	2.247371
H	-5.990585	-1.443814	-1.519547
H	0.815770	4.502540	-2.492817
H	-0.580691	3.734886	-3.262430
H	0.874752	2.772845	-2.913167
H	2.121284	-1.201596	-4.071475
H	1.370527	0.394664	-4.330377
H	0.367218	-0.993961	-3.835910
H	0.356178	-4.616912	2.620768
H	-1.040591	-3.764580	3.295993
H	0.489461	-2.892155	3.044090
H	-3.638716	1.209188	2.452838
H	4.779333	3.451641	1.674920
H	-1.937587	-4.045789	-0.370580
H	-2.430216	-4.540178	1.265325
H	-0.998533	-5.282108	0.501839
H	3.556020	-5.256661	-2.380781
H	5.240904	-5.398455	-1.814783
H	4.669597	-3.878610	-2.533209
H	1.992794	4.336288	-0.719422
H	-0.629116	5.247216	-0.465634
H	-1.697823	4.071919	0.339115

H	-2.812038	1.890739	-1.791966
H	3.042708	0.846166	-0.961769
H	2.818685	1.597723	-2.554886
H	3.758309	0.102769	-2.419582
H	5.149529	2.625287	-0.647434
H	5.946871	4.200270	-0.389388
H	4.423293	4.107827	-1.304998
H	5.389113	-2.586351	-0.351254
H	5.993991	-4.131138	0.294759
H	4.806402	-3.174906	1.221181
H	2.529487	-1.847890	2.825979
H	3.569242	-0.415135	2.758156
H	1.912556	0.995851	4.297581
H	1.048587	-0.549702	4.504865
H	0.168232	0.896925	3.946540
H	3.399874	5.867869	0.368747
H	4.930754	5.885946	1.268638
H	3.406464	5.496381	2.107755

109

$[4^{2+}]_2$ [AlCl4]-scf done: -8349.544325

C	0.001360	4.875559	2.080575
C	-0.226502	3.489524	2.286383
C	0.203606	2.804239	1.097629
C	0.718996	3.779180	0.178420
C	0.579792	5.051623	0.790886
Fe	-1.244417	4.092171	0.630145
C	-2.247515	3.832705	-1.119513
C	-2.687895	2.929243	-0.091164
C	-3.210268	3.706488	0.999584
C	-3.063599	5.074851	0.649190
C	-2.475959	5.151834	-0.646457
P	-2.622919	1.150760	-0.155277
C	-4.131107	0.403185	-0.771122
C	-5.114782	-0.051218	0.136020
C	-6.147678	-0.831055	-0.373501
C	-6.244187	-1.143167	-1.731344
C	-5.317087	-0.581055	-2.608674
C	-4.249720	0.193139	-2.163103
C	-5.143597	0.336560	1.605989
C	-6.288463	1.324770	1.864395
C	-7.296435	-2.107417	-2.237529
C	-8.713864	-1.728409	-1.801353
C	-3.303966	0.802840	-3.186111
C	-4.017031	1.905340	-3.978493
P	0.178854	1.032367	0.885640
P	-1.659884	-0.069832	1.415632
P	-0.860430	-0.043111	-0.737541
C	1.702323	0.214002	1.294383
C	1.903930	-0.378209	2.562805
C	3.052902	-1.140912	2.730311
C	3.983095	-1.328344	1.701561
C	3.776023	-0.678591	0.484995
C	2.657216	0.111340	0.253990

C	0.957638	-0.195546	3.737501
C	0.324182	-1.522441	4.166338
C	2.565608	0.891935	-1.045000
C	3.544048	2.072402	-1.003767
C	5.201163	-2.205682	1.892257
C	4.783722	-3.656095	2.157855
C	-5.203828	-0.874257	2.539932
C	-6.927409	-3.534174	-1.807838
C	-2.662263	-0.245257	-4.100412
C	2.752533	0.007900	-2.280488
C	6.119900	-1.654596	2.986441
C	1.654607	0.515398	4.903024
Cl	0.455526	-2.744527	0.214452
Al	-1.480838	-3.752686	0.240508
Cl	-1.351018	-5.807723	-0.241670
Cl	-2.358325	-3.377794	2.188520
Cl	-2.715965	-2.644182	-1.192036
Cl	6.933698	0.800444	0.154515
Al	7.231840	-0.034011	-1.839336
Cl	6.237980	1.260328	-3.263416
Cl	6.217954	-1.962166	-1.879953
Cl	9.315702	-0.246233	-2.262685
H	4.524880	-0.795845	-0.298367
H	-4.213643	0.881156	1.840656
H	0.140059	0.477385	3.422475
H	3.230831	-1.617417	3.697454
H	1.550737	1.329865	-1.120696
H	-2.480995	1.300714	-2.646208
H	5.761336	-2.180566	0.945425
H	1.132989	3.579945	-0.807820
H	-5.416849	-0.781411	-3.678112
H	-0.241376	5.668193	2.785319
H	-6.157324	-1.413108	2.432111
H	-5.128536	-0.550852	3.588966
H	-4.392805	-1.588905	2.336573
H	-3.337955	5.920752	1.276141
H	2.471786	-0.097636	5.311382
H	0.940890	0.704119	5.719656
H	2.085956	1.476517	4.585170
H	3.768103	-0.410892	-2.328116
H	2.609043	0.599744	-3.196385
H	2.039273	-0.831277	-2.278517
H	-1.802708	3.556112	-2.073883
H	-7.263722	-2.069522	-3.338798
H	-0.656119	3.028408	3.174277
H	3.354928	2.713620	-0.128136
H	3.458900	2.676955	-1.919839
H	4.583972	1.725089	-0.939236
H	-2.221999	6.066211	-1.178723
H	6.446566	-0.634876	2.738161
H	7.018361	-2.283481	3.074585
H	5.622734	-1.641166	3.970805
H	0.855544	6.001881	0.338349
H	-6.899474	-1.219203	0.316769

H	-7.265680	0.854030	1.677969
H	-6.212518	2.207388	1.210887
H	-6.274819	1.663377	2.911716
H	-3.623481	3.316506	1.928226
H	-0.164750	-2.037935	3.326954
H	-0.424758	-1.360669	4.956568
H	1.088919	-2.205840	4.565479
H	-5.904812	-3.792903	-2.117902
H	-7.623010	-4.262916	-2.250170
H	-6.976894	-3.639537	-0.712523
H	4.253464	-3.757885	3.119203
H	5.671587	-4.304644	2.195395
H	4.120639	-4.030038	1.363925
H	-2.184701	-1.048240	-3.520462
H	-1.905466	0.223448	-4.746719
H	-3.411322	-0.713031	-4.756853
H	-4.849770	1.492574	-4.567879
H	-3.318602	2.387965	-4.678984
H	-4.428557	2.676627	-3.309575
H	-8.838532	-1.806105	-0.709871
H	-9.446591	-2.408480	-2.260337
H	-8.967556	-0.700652	-2.101268

196

starting komplex of the formation of 4^{2+} SCF done: -7684.757027

C	-4.442513	0.382581	2.834317
C	-3.312162	-0.354489	2.414113
C	-2.072904	-0.242282	3.087185
C	-1.966594	0.692890	4.113443
C	-3.047115	1.483137	4.514897
C	-4.272908	1.290486	3.880079
P	-3.407362	-1.461213	0.987654
C	-4.182550	-3.044586	1.356722
C	-5.457760	-3.279969	1.979116
C	-5.833916	-4.618451	1.695760
C	-4.808893	-5.220183	0.912351
C	-3.785110	-4.262150	0.698164
Fe	-5.524975	-3.524843	-0.050701
C	-5.338496	-1.995249	-1.331000
C	-5.149445	-3.230589	-2.034496
C	-6.344029	-3.988926	-1.898214
C	-7.272387	-3.233807	-1.126442
C	-6.663010	-1.998559	-0.771664
P	-4.043256	-0.773584	-0.978595
P	-2.071517	-1.557234	-0.701535
C	-0.869137	-1.110252	2.750003
C	-0.266183	-1.765966	3.996646
C	-2.907311	2.489836	5.639216
C	-2.718784	1.781710	6.986226
C	-5.837919	0.205080	2.254379
C	-6.824112	-0.248116	3.340970
C	-4.446272	0.898548	-1.589969
C	-3.878165	2.041775	-0.970469

C	-4.334065	3.303516	-1.353541
C	-5.301907	3.481407	-2.334537
C	-5.759508	2.345939	-3.006576
C	-5.344357	1.054102	-2.693839
C	-2.713842	1.995726	0.005688
C	-2.800497	3.000995	1.154716
C	-5.756146	-0.084975	-3.628517
C	-6.822375	0.285668	-4.659869
C	-5.814294	4.860710	-2.694024
C	-5.486532	5.223781	-4.146723
C	-1.409967	2.170596	-0.781007
C	-7.314889	4.981691	-2.404485
C	-4.517857	-0.648853	-4.342211
C	0.175325	-0.314931	1.964526
C	-6.359739	1.460227	1.548266
C	-1.784743	3.496406	5.369774
C	1.229518	0.590905	-2.210995
C	1.084932	0.839164	-3.602443
C	2.027636	1.840589	-3.974425
C	2.760498	2.215301	-2.816807
C	2.277983	1.438515	-1.713932
Fe	2.986617	0.200713	-3.167947
C	3.217862	-1.822297	-3.090637
C	4.191225	-1.134486	-2.287340
C	4.971753	-0.290212	-3.156257
C	4.461454	-0.448523	-4.469243
C	3.390917	-1.386838	-4.429771
P	4.417521	-1.284110	-0.532664
C	5.033984	-2.878527	0.009106
C	6.426117	-3.057078	0.136557
C	6.881227	-4.304226	0.564358
C	6.007465	-5.354714	0.850532
C	4.633223	-5.143404	0.689822
C	4.118007	-3.921875	0.267653
P	2.639734	1.582023	0.055186
P	3.946591	0.061783	0.878125
C	7.410608	-1.933941	-0.141956
C	8.556560	-2.360156	-1.061499
C	6.534653	-6.698441	1.314448
C	6.214365	-7.801452	0.299246
C	2.615703	-3.744384	0.112560
C	1.994125	-4.793895	-0.812512
C	3.498299	3.167354	0.393178
C	4.799700	3.490848	-0.068166
C	5.354401	4.712058	0.309417
C	4.675563	5.628039	1.114126
C	3.390696	5.295958	1.538704
C	2.779906	4.087781	1.199075
C	5.647049	2.569908	-0.936031
C	6.207110	3.276417	-2.176733
C	1.390425	3.788448	1.751768
C	0.468882	5.008157	1.803888
C	5.314328	6.945606	1.506908
C	5.579670	7.823568	0.278738

C	1.498154	3.116596	3.125087
C	6.587578	6.726395	2.331337
C	6.787874	1.942232	-0.125635
C	7.916906	-1.324603	1.169323
C	6.018861	-7.048541	2.714586
C	1.926908	-3.703103	1.479209
H	0.649932	-0.117102	-1.618828
H	0.391342	0.335069	-4.272770
H	3.547278	2.962688	-2.772456
H	2.176264	2.240040	-4.975744
H	4.820331	0.072660	-5.354426
H	2.789309	-1.702175	-5.279818
H	2.473860	-2.531908	-2.734515
H	5.799737	0.348027	-2.856488
H	-7.120330	-1.198526	-0.192306
H	-8.272528	-3.552492	-0.839427
H	-4.239656	-3.537799	-2.547016
H	-6.510790	-4.987010	-2.298332
H	-4.814315	-6.238422	0.528752
H	-6.757997	-5.095837	2.015648
H	-6.042181	-2.566513	2.551198
H	-2.880274	-4.400132	0.109645
H	-1.010993	0.798525	4.631920
H	-5.132439	1.876112	4.215955
H	-6.460268	2.484059	-3.828331
H	-3.913272	4.185272	-0.867049
H	7.955214	-4.468241	0.680503
H	3.943446	-5.964322	0.900939
H	2.850289	6.010336	2.162622
H	6.360396	4.961577	-0.035719
H	-1.211925	-1.940173	2.107436
H	-3.854598	3.051128	5.685939
H	-5.793163	-0.597559	1.502541
H	0.511226	-2.486662	3.706022
H	0.207567	-1.026052	4.658651
H	-1.030150	-2.302122	4.577653
H	1.026069	-0.956277	1.688654
H	-0.247206	0.106745	1.040824
H	0.564643	0.520290	2.564867
H	-1.785394	4.283570	6.137617
H	-0.795154	3.013987	5.400044
H	-1.901058	3.979792	4.387770
H	-2.679529	2.515698	7.804345
H	-3.544690	1.085526	7.192214
H	-1.778803	1.207670	7.005934
H	-7.772672	-0.572961	2.887185
H	-6.424266	-1.074540	3.946946
H	-7.058971	0.574130	4.032883
H	-7.366423	1.278417	1.141972
H	-6.438495	2.305395	2.249118
H	-5.712185	1.767262	0.717940
H	-2.676177	1.004771	0.465109
H	-6.197420	-0.888583	-3.030204
H	-7.109638	-0.615164	-5.220541

H	-7.732581	0.683258	-4.188489
H	-6.458620	1.021308	-5.393230
H	-4.795958	-1.499301	-4.981835
H	-4.054709	0.122172	-4.977241
H	-3.741640	-1.004197	-3.644384
H	-5.288447	5.578523	-2.043280
H	-7.661537	6.006760	-2.600851
H	-7.902449	4.304630	-3.044370
H	-7.542279	4.740081	-1.355682
H	-5.798161	6.256345	-4.361495
H	-4.407913	5.144376	-4.347228
H	-6.013868	4.566540	-4.855797
H	-2.043834	2.750657	1.914136
H	-2.602944	4.030578	0.820535
H	-3.782890	2.981950	1.644998
H	-0.539205	2.043439	-0.119536
H	-1.330763	1.432537	-1.590760
H	-1.353384	3.173867	-1.231026
H	0.911978	3.064001	1.073903
H	4.999779	1.757864	-1.294291
H	-0.550452	4.690595	2.066756
H	0.782395	5.736845	2.566236
H	0.427132	5.525453	0.834098
H	0.500422	2.835057	3.495526
H	2.120367	2.209119	3.081191
H	1.955445	3.797551	3.859662
H	4.589834	7.476155	2.146193
H	6.995616	7.689964	2.670036
H	6.389491	6.108816	3.219701
H	7.369918	6.227060	1.738035
H	5.976193	8.802592	0.585364
H	6.321189	7.361539	-0.392188
H	4.659451	7.994093	-0.299164
H	7.631324	-6.609641	1.375534
H	6.666271	-8.753412	0.613949
H	5.128297	-7.963519	0.211029
H	6.603003	-7.553650	-0.699331
H	6.471919	-7.987544	3.064517
H	6.263676	-6.260659	3.441941
H	4.926084	-7.187925	2.718171
H	2.439346	-2.759789	-0.358864
H	0.932659	-4.563764	-0.992131
H	2.510945	-4.831644	-1.783075
H	2.042548	-5.801510	-0.373169
H	0.848195	-3.511466	1.365893
H	2.046298	-4.659004	2.011935
H	2.356957	-2.913349	2.114153
H	6.857977	-1.136519	-0.675114
H	6.672086	2.548238	-2.859281
H	5.430821	3.819109	-2.735427
H	6.986299	4.006193	-1.912458
H	7.357397	1.226499	-0.740231
H	7.491523	2.712837	0.224029
H	6.420255	1.410900	0.765138

H	8.594566	-0.480963	0.971409
H	7.083624	-0.960399	1.790045
H	8.469991	-2.071530	1.759158
H	9.174659	-1.489021	-1.324125
H	9.219281	-3.091337	-0.574964
H	8.179808	-2.808810	-1.992142

196

intermedier during the formation of 4^{2+} scf done: -7684.794477

C	-2.015880	-2.360779	2.290461
C	-1.774295	-1.101818	1.648228
C	-1.567456	0.065841	2.443946
C	-1.611579	-0.064934	3.834688
C	-1.854472	-1.268182	4.479953
C	-2.050947	-2.393305	3.679429
P	-1.760876	-0.966309	-0.180284
P	-2.472202	0.952002	-1.207319
C	-3.966476	1.737053	-0.493732
C	-3.958429	3.148755	-0.681276
C	-5.077637	3.880616	-0.281235
C	-6.181434	3.292064	0.329146
C	-6.142573	1.915397	0.544853
C	-5.069444	1.119045	0.149500
C	-2.796413	3.903455	-1.327356
C	-2.636889	5.336473	-0.816013
C	-7.371548	4.120346	0.769353
C	-7.526758	4.098434	2.294331
C	-5.143064	-0.350189	0.533733
C	-5.089054	-0.543322	2.055253
C	-1.333656	1.502682	1.971257
C	-0.076164	2.131670	2.602544
C	-1.871524	-1.405411	5.989216
C	-2.236977	-0.117733	6.725671
C	-2.197816	-3.685884	1.557442
C	-2.872395	-4.788862	2.375263
P	0.222594	-0.528819	-1.194831
P	1.514065	0.290958	0.388931
P	1.915903	2.226821	-0.612343
C	3.087454	3.224389	0.379982
C	4.378947	2.830352	0.803294
C	5.173342	3.751986	1.487446
C	4.746106	5.047389	1.767035
C	3.467884	5.414556	1.346596
C	2.623373	4.541527	0.663223
C	4.928723	1.422068	0.640003
C	6.356135	1.371094	0.083909
C	5.638933	6.026288	2.503548
C	5.987868	7.231576	1.623066
C	1.280527	5.076985	0.168710
C	0.651832	6.116814	1.098615
P	2.106646	-1.583175	-0.637581
C	3.131568	-1.369085	-2.088647
C	4.539136	-1.080068	-2.051545
C	5.009263	-1.066497	-3.388859

C	3.914923	-1.344446	-4.256596
C	2.750330	-1.522693	-3.468233
Fe	3.504052	0.333124	-3.138450
C	2.890712	1.729646	-4.517531
C	1.978913	1.674194	-3.431403
C	2.700528	1.978729	-2.227407
C	4.056142	2.245488	-2.599177
C	4.170762	2.082494	-4.004105
C	2.313319	-3.175189	0.221654
C	2.603343	-3.256027	1.610523
C	2.671263	-4.520426	2.192091
C	2.489011	-5.698868	1.472131
C	2.241136	-5.588927	0.105979
C	2.133737	-4.361777	-0.542603
C	2.811682	-2.065009	2.529022
C	1.522555	-1.741951	3.289179
C	1.867952	-4.386344	-2.038644
C	0.786597	-5.392573	-2.446879
C	2.505330	-7.049386	2.156462
C	1.224022	-7.229759	2.982232
C	-2.358048	-2.355308	-1.205704
C	-3.706828	-2.841500	-1.361438
C	-3.731957	-3.710416	-2.483321
C	-2.423972	-3.777030	-3.033698
C	-1.569406	-2.960600	-2.248412
Fe	-3.077569	-1.806558	-2.975960
C	-2.972786	-0.866750	-4.817961
C	-2.187358	-0.191831	-3.844886
C	-3.051361	0.178151	-2.755349
C	-4.378227	-0.237135	-3.101426
C	-4.321533	-0.891244	-4.361057
C	-2.914529	3.891647	-2.858133
C	-8.656409	3.675553	0.062403
C	-6.381703	-1.055369	-0.031446
C	3.171547	-4.648355	-2.804914
C	3.761891	-7.264623	3.004297
C	3.985715	-2.249634	3.495062
C	1.436506	5.627242	-1.256181
C	5.014939	6.460110	3.834485
C	-2.529078	2.407317	2.314998
C	-0.525087	-1.961613	6.476023
C	-0.858498	-4.183113	1.011803
C	4.871231	0.696128	1.987407
H	0.916016	1.448472	-3.493480
H	2.656409	1.520826	-5.559702
H	4.852517	2.536351	-1.921023
H	5.084431	2.193971	-4.584813
H	6.030982	-0.854610	-3.697515
H	3.955970	-1.379687	-5.343384
H	1.750172	-1.735747	-3.840947
H	5.139071	-0.913878	-1.159965
H	-5.265074	-0.080564	-2.493237
H	-5.164854	-1.337621	-4.884316
H	-1.115196	-0.012338	-3.898486

H	-2.607935	-1.292029	-5.750746
H	-2.124686	-4.347241	-3.910357
H	-4.614716	-4.220427	-2.863322
H	-4.564280	-2.603859	-0.742733
H	-0.508095	-2.812688	-2.425422
H	-5.090446	4.959899	-0.438129
H	-6.984628	1.442437	1.055349
H	3.121128	6.427453	1.554705
H	6.167227	3.451072	1.827093
H	2.111609	-6.504194	-0.474421
H	2.874761	-4.585988	3.261453
H	-2.242182	-3.343242	4.177859
H	-1.458776	0.830342	4.436168
H	-4.256332	-0.837419	0.106713
H	-1.867197	3.373314	-1.061062
H	-7.167714	5.161087	0.469282
H	-9.492724	4.334252	0.338242
H	-8.544762	3.707298	-1.031285
H	-8.937679	2.648996	0.346279
H	-8.348053	4.760165	2.605938
H	-7.761263	3.085823	2.659451
H	-6.607855	4.436299	2.795992
H	-2.055577	4.405212	-3.315689
H	-2.955173	2.871357	-3.269130
H	-3.831564	4.409844	-3.178182
H	-1.687351	5.751472	-1.182467
H	-3.433175	6.003034	-1.178554
H	-2.628026	5.381392	0.283387
H	-6.395328	-2.114186	0.271596
H	-7.307445	-0.608582	0.357911
H	-6.431590	-1.014638	-1.128183
H	-5.020198	-1.613651	2.303598
H	-4.226728	-0.038803	2.508577
H	-5.999238	-0.148783	2.531593
H	-2.863087	-3.508851	0.709629
H	-3.101308	-5.636640	1.713549
H	-2.225166	-5.175798	3.176809
H	-3.817862	-4.452322	2.825108
H	-0.995698	-5.094756	0.411731
H	-0.366420	-3.438941	0.371435
H	-0.162376	-4.413630	1.832608
H	-2.643879	-2.157734	6.222496
H	-0.544836	-2.118022	7.564290
H	-0.288325	-2.925063	6.000069
H	0.291492	-1.258649	6.247952
H	-2.369177	-0.322548	7.797333
H	-1.444156	0.642119	6.640177
H	-3.174520	0.316592	6.347918
H	-1.192590	1.518054	0.878445
H	0.215487	3.033299	2.043484
H	-0.269722	2.449916	3.637235
H	0.779496	1.445671	2.626785
H	-3.484757	2.011984	1.958655
H	-2.604988	2.544120	3.404000

H	-2.387749	3.400742	1.864048
H	0.575045	4.232178	0.115334
H	6.578458	5.496598	2.731828
H	-0.377145	6.329548	0.777012
H	0.618105	5.762907	2.140003
H	1.192144	7.074911	1.082343
H	0.477175	6.006215	-1.639339
H	2.159020	6.457596	-1.273883
H	1.799432	4.857985	-1.956126
H	6.693366	7.895454	2.143796
H	6.451387	6.916759	0.676594
H	5.091841	7.824766	1.381376
H	5.706435	7.112645	4.387351
H	4.082828	7.025086	3.675853
H	4.782194	5.593273	4.470493
H	1.495206	-3.394444	-2.332914
H	2.501055	-7.813460	1.362228
H	3.061439	-1.199738	1.905206
H	3.777812	-8.288706	3.404015
H	4.676951	-7.117194	2.412542
H	3.797058	-6.578632	3.864991
H	1.195387	-8.231607	3.434870
H	1.173257	-6.491442	3.798831
H	0.324617	-7.111679	2.359182
H	4.188794	-1.303632	4.017366
H	3.772457	-3.000066	4.270077
H	4.902901	-2.555755	2.970822
H	1.647353	-0.834108	3.899141
H	0.676504	-1.576626	2.608471
H	1.250425	-2.567689	3.964256
H	0.508694	-5.232454	-3.499454
H	1.141553	-6.430101	-2.369077
H	-0.118990	-5.294458	-1.832305
H	3.004682	-4.578536	-3.889983
H	3.960718	-3.934405	-2.532232
H	3.543375	-5.660254	-2.583593
H	4.281210	0.886055	-0.071223
H	5.191400	-0.350921	1.883429
H	5.540999	1.176415	2.716628
H	3.857164	0.718517	2.415275
H	6.670937	0.327070	-0.068820
H	6.458107	1.896494	-0.875670
H	7.080671	1.815248	0.781316

79

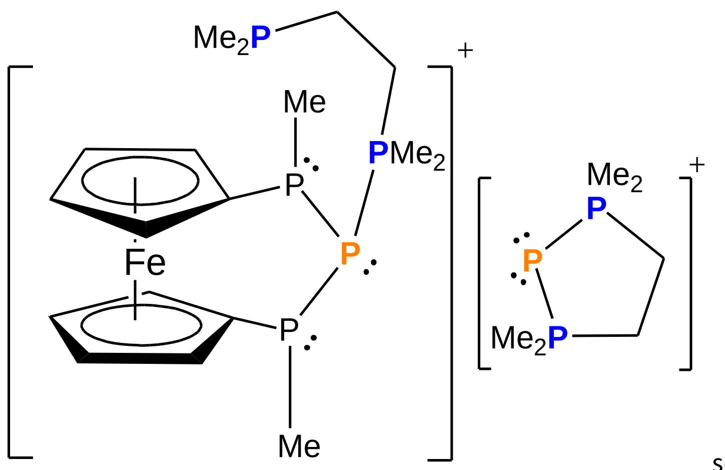

scf done: -4934.492702

C	-2.007727	-3.112357	-2.698858
C	-2.119275	-1.756079	-2.292871
C	-0.869852	-1.359465	-1.703542
C	0.009678	-2.496222	-1.772676
C	-0.697384	-3.567862	-2.377991
Fe	-1.655713	-2.924144	-0.670423
C	-2.961086	-2.568505	0.860407
C	-3.155877	-3.889388	0.371652
C	-1.930290	-4.597242	0.509198
C	-0.970467	-3.717689	1.075527
C	-1.598170	-2.442772	1.293841
P	-0.629281	-1.045098	1.921551
C	-1.293764	-0.765845	3.622128
P	-0.278623	0.259028	-1.135408
C	-1.072476	1.386000	-2.359103
P	-1.146564	0.816129	0.847486
P	-3.335177	1.251192	0.746736
C	-4.322976	0.596310	-0.626485
C	-3.569115	3.066980	0.766575
C	-2.964665	3.925434	-0.347789
P	-1.129841	4.292419	-0.220555
C	-1.067259	5.696618	-1.414382
C	-4.142505	0.726101	2.284966
C	-1.079506	5.195967	1.385885
P	3.105004	-0.608368	0.265547
P	5.157726	-0.739617	0.898028
C	6.171902	-1.949341	0.000736
P	3.565922	1.176548	-0.839518
C	5.378035	1.480103	-0.686388
C	5.900169	0.916960	0.634232
C	3.207880	1.089367	-2.614440
C	2.703700	2.645847	-0.213292
C	5.294579	-1.165333	2.652321
H	-0.860168	-1.554317	4.255480
H	-0.815692	2.426131	-2.119321
H	-2.384768	-0.834793	3.706519
H	-0.943912	0.202379	4.008740
H	-2.161709	1.277624	-2.441205
H	-0.642443	1.129398	-3.339354
H	0.072435	-3.953139	1.281913

H	-1.751815	-5.627341	0.207055
H	-4.078904	-4.286074	-0.046929
H	-3.729284	-1.802006	0.896135
H	1.034555	-2.521223	-1.403084
H	-0.313172	-4.571894	-2.547158
H	-2.793990	-3.704284	-3.163579
H	-2.996536	-1.130729	-2.438290
H	-4.666577	3.186308	0.776256
H	-3.220382	3.399751	1.757941
H	-5.386198	0.788991	-0.419138
H	-4.050522	1.097561	-1.564150
H	-4.154571	-0.482969	-0.731326
H	-5.173011	1.111515	2.286055
H	-4.167397	-0.367087	2.371702
H	-3.603995	1.145457	3.145609
H	-3.504816	4.886451	-0.343604
H	-3.154648	3.481783	-1.339453
H	-0.058743	6.135411	-1.405927
H	-1.262791	5.331962	-2.434022
H	-1.794966	6.488104	-1.176298
H	-0.115625	5.720934	1.461484
H	-1.885642	5.940405	1.482984
H	-1.128066	4.491166	2.229232
H	3.511512	2.021417	-3.113689
H	3.743590	0.235560	-3.050741
H	2.128855	0.934177	-2.747989
H	2.969766	3.530232	-0.811087
H	1.615827	2.486585	-0.264661
H	2.981948	2.804410	0.837553
H	5.578394	2.558530	-0.778060
H	5.861054	0.988116	-1.545246
H	6.998879	0.848477	0.642270
H	5.599508	1.545182	1.486968
H	5.715537	-2.944185	0.105985
H	6.193780	-1.694819	-1.067780
H	7.196842	-1.976312	0.399595
H	4.854971	-2.159101	2.819649
H	6.350785	-1.186775	2.958257
H	4.741850	-0.428220	3.249810

79

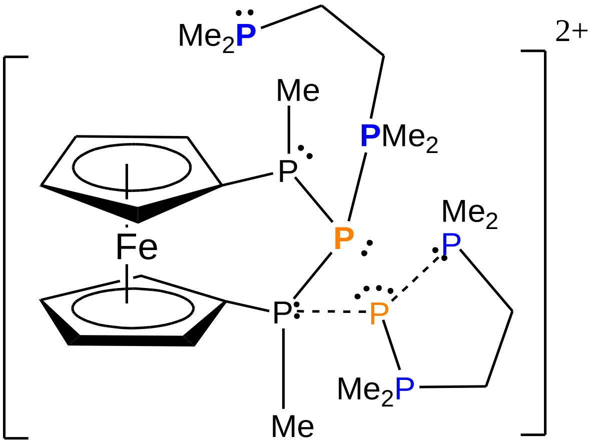

scf done: -4934.457916

C	-3.245973	-0.772432	1.250243
C	-3.897093	-0.359978	0.036174
C	-4.813275	-1.376724	-0.349702
C	-4.748104	-2.418318	0.615587
C	-3.789456	-2.056267	1.598416
Fe	-2.877615	-2.088168	-0.228712
C	-1.812704	-3.837289	-0.520632
C	-2.373333	-3.397462	-1.754039
C	-1.898519	-2.086453	-2.019780
C	-1.027855	-1.702193	-0.936245
C	-0.979425	-2.805712	-0.013827
P	-0.009671	-0.216451	-0.815903
P	2.125015	-0.322104	0.261984
P	3.004758	-1.768046	-1.106920
C	4.054915	-0.974769	-2.363154
P	-1.775228	-0.143819	2.113753
P	-0.694012	1.237022	0.782281
P	-1.977793	2.908996	-0.009605
C	-3.510891	3.054625	0.952256
C	-2.403252	0.949922	3.461964
C	-0.191953	0.546198	-2.465944
C	-2.440600	2.968444	-1.761811
C	-1.084983	4.462325	0.390805
C	0.024456	4.901369	-0.564637
P	1.298356	3.590155	-0.912182
C	2.293783	3.607721	0.633646
C	2.392487	4.510139	-2.070077
C	4.089096	-2.948304	-0.205913
C	5.202907	-2.239633	0.567516
P	4.490094	-0.794910	1.477667
C	5.913844	0.310787	1.783384
C	1.869738	-2.837564	-2.040563
C	4.055017	-1.449313	3.126986
H	-2.712147	0.279181	4.277800
H	0.333823	1.509856	-2.460019
H	-3.265710	1.569873	3.193593
H	-1.584645	1.575811	3.846625
H	-1.240952	0.667122	-2.763585
H	0.294799	-0.125699	-3.188430
H	-3.488237	-2.667052	2.448704
H	-5.315720	-3.346556	0.592521
H	-5.439300	-1.369121	-1.239953
H	-3.688742	0.540054	-0.536517
H	-0.417841	-2.821419	0.919851
H	-2.011572	-4.790633	-0.034570
H	-3.067161	-3.958688	-2.377085
H	-2.158573	-1.488693	-2.891041
H	-1.868610	5.236476	0.451649
H	-0.704196	4.323645	1.416624
H	-3.033096	3.882905	-1.918591
H	-1.542088	3.009711	-2.389180
H	-3.049675	2.098258	-2.041350
H	-4.124229	3.845550	0.495205
H	-4.079792	2.116980	0.970349

H	-3.265051	3.355000	1.979472
H	0.493697	5.810852	-0.154788
H	-0.410418	5.192933	-1.535054
H	3.283396	3.901877	-2.283816
H	1.872403	4.688670	-3.022490
H	2.718907	5.476090	-1.655047
H	3.161124	2.945896	0.492980
H	2.645735	4.618258	0.892066
H	1.706183	3.194789	1.466484
H	2.471554	-3.517971	-2.660914
H	1.245106	-3.423471	-1.353999
H	1.215384	-2.252512	-2.699401
H	4.562726	-1.723000	-2.990033
H	3.426707	-0.329675	-2.993494
H	4.800676	-0.338170	-1.867504
H	4.494433	-3.665567	-0.937920
H	3.425097	-3.511117	0.469926
H	5.717260	-2.940589	1.243246
H	5.966108	-1.852479	-0.126475
H	3.620174	-0.641412	3.733233
H	3.293237	-2.234038	3.018551
H	4.933407	-1.856463	3.649614
H	5.588652	1.157429	2.404902
H	6.727723	-0.217124	2.303183
H	6.291708	0.709240	0.831149

55

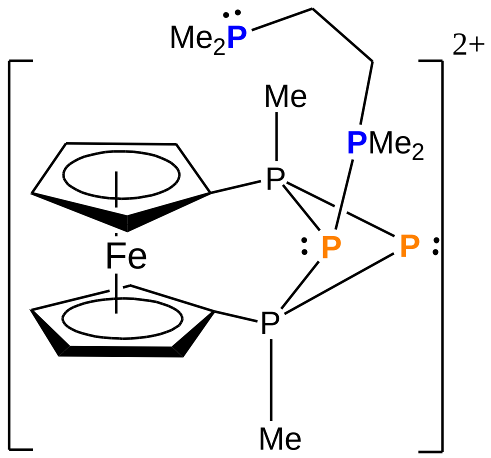

scf done: -4013.863614

C	-2.150950	1.558790	-1.027159
C	-2.230995	1.186193	0.359877
C	-3.526571	0.598675	0.576870
C	-4.217658	0.609671	-0.661458
C	-3.375780	1.194591	-1.646446
Fe	-2.443642	-0.453719	-0.795937
C	-0.870184	-1.511035	-1.591016
C	-1.035788	-1.754998	-0.186778
C	-2.345512	-2.318420	0.014625
C	-2.969120	-2.404314	-1.256503
C	-2.070600	-1.903424	-2.239173
P	0.050771	-1.331524	1.166647
C	1.144256	-2.719759	1.533385
P	-0.966611	1.224697	1.633303

C	-1.031706	2.792624	2.534409
P	-0.907480	-0.514432	2.911621
P	1.088930	0.622370	0.973436
P	1.706666	1.703457	-0.933180
C	3.533959	1.814815	-0.884876
C	4.244680	0.502784	-1.219648
P	3.595917	-0.891593	-0.183067
C	4.642443	-0.798551	1.321190
C	1.237031	1.150510	-2.591512
C	1.121534	3.403669	-0.678504
C	4.240512	-2.371798	-1.052300
H	-1.999665	2.847959	3.055627
H	1.891689	-2.425787	2.281859
H	-0.966192	3.638597	1.836139
H	-0.218471	2.833265	3.271591
H	1.621240	-3.078061	0.611960
H	0.513647	-3.524793	1.943501
H	-3.897572	0.192084	1.516777
H	-5.217045	0.212936	-0.830505
H	-3.622982	1.334025	-2.697199
H	-1.325268	2.055559	-1.527055
H	-2.786195	-2.601106	0.969657
H	-3.978060	-2.768584	-1.441332
H	-2.271231	-1.829137	-3.306404
H	0.021771	-1.126027	-2.074224
H	3.810998	2.617844	-1.588938
H	3.796075	2.175948	0.123640
H	1.616755	1.887637	-3.316281
H	1.700926	0.177012	-2.800477
H	0.148285	1.062136	-2.692278
H	1.516866	4.044883	-1.480955
H	0.025284	3.465850	-0.686192
H	1.500431	3.777570	0.284328
H	5.330848	0.629831	-1.088142
H	4.093186	0.241394	-2.279366
H	4.053546	-3.268542	-0.443605
H	3.731927	-2.502557	-2.018231
H	5.324667	-2.293362	-1.225006
H	4.407282	-1.641149	1.988002
H	5.713402	-0.843531	1.072328
H	4.438346	0.130018	1.873822

24

Me2P(CH2)2PMe2 scf done: -920.534643

P	0.028256	-0.236320	0.230358
C	0.430323	0.289364	1.959881
C	1.734019	-0.064922	-0.509641
C	-0.751630	1.317932	-0.398441
H	-0.986350	1.198745	-1.466782
H	-1.700267	1.489021	0.132451
H	-0.106313	2.201920	-0.264813
C	1.778524	-0.033727	-2.040401
H	2.223423	0.840643	-0.109645
H	2.316899	-0.924287	-0.136400

H	-0.497003	0.357023	2.548452
H	1.070052	-0.470114	2.434674
H	0.947510	1.262202	1.999369
H	2.824668	-0.134465	-2.379474
P	0.716439	-1.313125	-2.890752
H	1.426361	0.946538	-2.404583
C	1.483156	-1.224550	-4.574065
C	1.512968	-2.859665	-2.262534
H	1.049845	-2.005604	-5.216818
H	1.250805	-0.251094	-5.032191
H	2.578000	-1.351771	-4.549997
H	1.147305	-3.718978	-2.844676
H	2.613095	-2.828635	-2.329296
H	1.217707	-3.019175	-1.214675

79

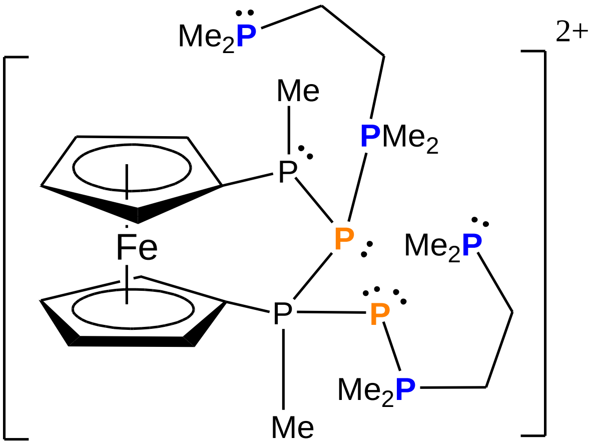

scf done: -4934.466337

C	-2.307967	2.722969	-1.196773
C	-1.808119	1.630181	-0.444940
C	-0.739289	1.033210	-1.200408
C	-0.594341	1.785760	-2.425297
C	-1.568035	2.817389	-2.410580
Fe	-0.293485	2.956193	-0.781159
C	-0.020337	4.075047	0.905523
C	0.922262	2.994101	0.824642
C	1.679059	3.190519	-0.382548
C	1.200100	4.364223	-1.026182
C	0.156904	4.909172	-0.229632
P	0.769661	1.577468	1.952367
C	1.931171	1.902706	3.349319
P	0.166007	-0.443812	-0.727114
P	-0.947677	-1.910414	0.338974
P	-2.628802	-2.230850	-0.998429
C	-3.944585	-2.954844	0.055279
C	-4.218721	-2.276188	1.399962
P	-4.708565	-0.476670	1.321281
C	-4.974269	-0.173814	3.117146
P	1.633105	-0.205938	0.979853
P	3.757503	0.066401	0.269379
C	4.127970	0.150946	-1.502869
C	4.715444	-1.321402	0.993126

C	4.693348	-2.653176	0.244197
P	2.983314	-3.279136	-0.135164
C	2.437320	-3.910515	1.502181
C	4.493302	1.531756	1.047721
C	3.396569	-4.834976	-1.023128
C	1.003677	-0.946234	-2.260819
C	-3.331496	-0.854503	-1.952452
C	-2.259168	-3.515576	-2.230867
C	-6.447788	-0.632919	0.723081
H	1.410168	2.609046	4.013280
H	1.616407	-1.832122	-2.047896
H	2.888269	2.353673	3.065034
H	2.094338	0.978690	3.923392
H	1.607360	-0.131291	-2.680000
H	0.214824	-1.203356	-2.983168
H	-0.772464	4.208976	1.682196
H	-0.426738	5.797645	-0.462854
H	1.555549	4.764133	-1.973968
H	2.449326	2.534223	-0.779277
H	-2.179490	1.281304	0.518724
H	-3.107081	3.392159	-0.883790
H	-1.704522	3.568449	-3.186473
H	0.120438	1.606303	-3.225848
H	5.747772	-0.940939	1.075753
H	4.340951	-1.430012	2.025036
H	5.218638	0.258469	-1.608071
H	3.808857	-0.773264	-1.999824
H	3.640201	1.016611	-1.971057
H	5.506855	1.658970	0.638538
H	3.908367	2.440727	0.861638
H	4.574702	1.361949	2.129727
H	5.263644	-3.390131	0.833137
H	5.224293	-2.552775	-0.716909
H	2.471270	-5.397963	-1.213144
H	3.857488	-4.607558	-1.995330
H	4.080975	-5.472726	-0.443037
H	1.473482	-4.425224	1.376369
H	3.162935	-4.613410	1.939219
H	2.268082	-3.075752	2.197841
H	-4.189869	-1.246818	-2.518436
H	-3.665682	-0.062914	-1.269591
H	-2.593296	-0.448514	-2.657403
H	-3.176379	-3.803236	-2.766800
H	-1.524126	-3.139672	-2.956067
H	-1.837563	-4.395064	-1.725603
H	-3.647084	-4.004017	0.217047
H	-4.847087	-2.987064	-0.579035
H	-5.001667	-2.855504	1.917115
H	-3.318708	-2.331098	2.033760
H	-6.938285	0.350316	0.776870
H	-6.467984	-0.946523	-0.331871
H	-7.035097	-1.349282	1.318640
H	-5.419821	0.822259	3.254262
H	-5.640018	-0.922005	3.574549

H	-4.007852	-0.185418	3.642175

31

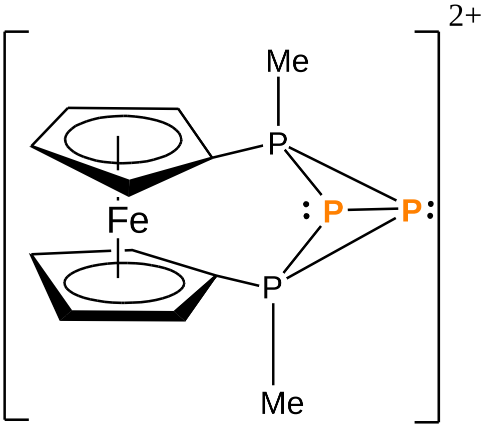

scf done: -3093.225177

C	0.234582	0.240678	-0.133904
C	-0.026352	0.000224	1.237624
C	1.211211	-0.114301	1.932272
C	2.274665	0.051833	1.011189
C	1.670851	0.277259	-0.281330
Fe	1.105957	1.722976	0.963524
C	0.280964	3.472794	0.316941
C	0.020676	3.335749	1.702721
C	1.257839	3.221127	2.398052
C	2.320369	3.283912	1.463164
C	1.716351	3.437641	0.160273
P	2.583867	3.531352	-1.368645
C	3.033768	5.198311	-1.893207
P	2.541240	0.582893	-1.780748
C	2.945923	-0.888260	-2.744293
P	4.136293	2.085045	-1.937549
H	2.006004	-1.405106	-2.992581
H	3.569522	5.185232	-2.852991
H	3.562929	-1.550119	-2.117008
H	3.487783	-0.627919	-3.664603
H	3.668859	5.644266	-1.112171
H	2.110032	5.790469	-1.984210
H	-0.509592	0.364055	-0.919662
H	-1.016254	-0.065391	1.686545
H	1.327114	-0.282306	3.001890
H	3.338644	0.007795	1.240199
H	-0.462835	3.590297	-0.470077
H	-0.969020	3.303418	2.155710
H	1.373648	3.086376	3.472378
H	3.384098	3.233311	1.691976
P	2.071336	2.277083	-3.097519
